# Kaxiras’s Porphyrin: DFT Modeling of Redox-Tuned Optical and Electronic Properties in a Theoretically Designed Catechol-Based Bioinspired Platform

**DOI:** 10.3390/biomimetics2040021

**Published:** 2017-11-07

**Authors:** Orlando Crescenzi, Marco d’Ischia, Alessandra Napolitano

**Affiliations:** Department of Chemical Sciences, University of Naples Federico II, I-80126 Naples, Italy; dischia@unina.it (M.d.I.); alesnapo@unina.it (A.N.)

**Keywords:** porphyrin, eumelanin, DFT, *ab initio* calculation

## Abstract

A detailed computational investigation of the 5,6-dihydroxyindole (DHI)-based porphyrin-type tetramer first described by Kaxiras as a theoretical structural model for eumelanin biopolymers is reported herein, with a view to predicting the technological potential of this unique bioinspired tetracatechol system. All possible tautomers/conformers, as well as alternative protonation states, were explored for the species at various degrees of oxidation and all structures were geometry optimized at the density functional theory (DFT) level. Comparison of energy levels for each oxidized species indicated a marked instability of most oxidation states except the six-electron level, and an unexpected resilience to disproportionation of the one-electron oxidation free radical species. Changes in the highest energy occupied molecular orbital (HOMO)–lowest energy unoccupied molecular orbital (LUMO) gaps with oxidation state and tautomerism were determined along with the main electronic transitions: more or less intense absorption in the visible region is predicted for most oxidized species. Data indicated that the peculiar symmetry of the oxygenation pattern pertaining to the four catechol/quinone/quinone methide moieties, in concert with the NH centers, fine-tunes the optical and electronic properties of the porphyrin system. For several oxidation levels, conjugated systems extending over two or more indole units play a major role in determining the preferred tautomeric state: thus, the highest stability of the six-electron oxidation state reflects porphyrin-type aromaticity. These results provide new clues for the design of innovative bioinspired optoelectronic materials.

## 1. Introduction

**Scheme 1:**
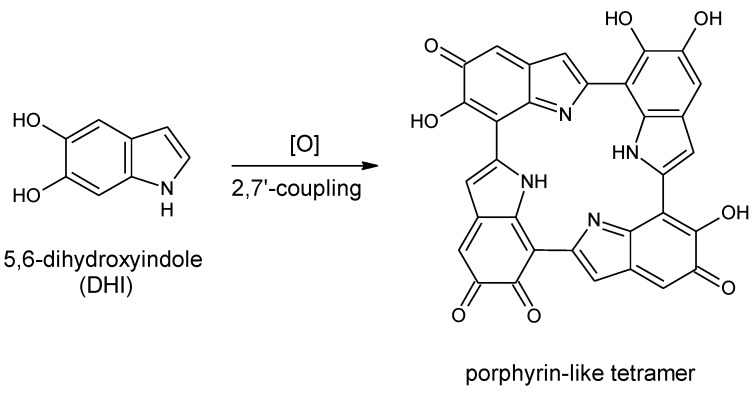
Formation of a porphyrin-like tetramer (Kaxiras’s porphyrin, KP) by oxidation of 5,6-dihydroxyindole (DHI).

Eumelanins, according to a recent consensus definition, are “a black-to-brown insoluble subgroup of melanin pigments derived at least in part from the oxidative polymerization of l-dopa via 5,6-dihydroxyindole (DHI) intermediates” [1]. Among organic polymers eumelanins occupy a unique position because of (i) their widespread occurrence in nature, from man and mammals to fish, birds and molluscs; (ii) the variety of biological roles, from photoprotection to scavenging of reactive oxygen species and metal chelation; and (iii) a unique set of physical and chemical properties, including broadband absorption throughout the visible range, water-dependent ionic-electronic semiconductor-like behavior, stable free radical character and efficient nonradiative energy dissipation, making them attractive candidates for biomedical and technological applications. Despite growing interest in eumelanin-type functional materials and systems, a major gap hindering progress toward melanin-based technology relates to the highly insoluble and heterogeneous character of these polymers, which has so far hindered detailed insights into the structural basis of their physicochemical properties. In 2006 Kaxiras et al. [2] proposed on a theoretical basis that eumelanin properties could be interpreted in terms of porphyrin-type building blocks derived from the oxidative cyclization of DHI tetramers built via 2,7′-coupling (Scheme 1).

Density functional theory (DFT) calculations indicated broadband absorption properties compatible with the optical properties of eumelanins. In a subsequent study, the authors suggested that such tetramers could bind in a covalent manner through interlayer C–C bonds [3].

Inspired by these theoretical studies, several authors adopted Kaxiras’s porphyrin (KP) model to explain eumelanin properties [4,5,6,7], despite the lack of more than circumstantial or indirect evidence for porphyrin-type structures. Yet, regardless of its actual relevance to eumelanin structure, KP is a noticeable example of polycyclic aromatic system built upon a key eumelanin building block and displaying catechol-controlled electron properties, including aromaticity. This system, which is conceptually related to the basic polycyclic scaffold of electroluminescent DHI-based triazatruxenes [8], displays potential optoelectronic properties that seem worthy of being investigated at a theoretical level in the quest for novel bioinspired functional systems. Of special interest, to this aim, is the impact of the oxidation state on the thermodynamic stability of the porphyrin scaffold and the structural and redox dependence of the highest energy occupied molecular orbital (HOMO)– lowest energy unoccupied molecular orbital (LUMO) gap, which is critical for material design in organic electronics.

The aim of this paper is to integrate and expand previous computational studies on KPs in order to draw a first set of fundamental structure–property relationships. Reported herein is a detailed investigation of the structure and properties of this unique molecular platform as a function of the oxidation state, with special reference to:

(a) The relative stability of the various oxidation states;
(b) The position of tautomeric and acid-base dissociation equilibriums in the various oxidation states;
(c) The spectroscopic (ultraviolet–visible (UV–Vis) and infrared (IR)) properties of the most stable tautomers for each of the possible oxidation states;
(d) The nature of main electronic transitions;
(e) The aromatic character of each oxidation state.

It is expected that elucidation of KPs properties may guide the design of innovative catechol-based redox tunable systems for bioinspired applications.

## 2. Methods

All calculations were performed with the Gaussian program package [9]. All structures were geometry optimized at the DFT level, with a hybrid functional (PBE0) [10] and a reasonably large basis set (6-31+G(d,p)). For each species, different tautomers/conformers, as well as different protonation states were explored. In those cases where conformational enantiomers exist, a single enantiomeric series has been explored.

Computations were performed either in vacuo, or by adoption of a polarizable continuum model (PCM) [11,12,13,14] to account for the influence of the solution environment. In view of the faster convergence, a scaled van der Waals cavity based on universal force field (UFF) radii [15] was used, and polarization charges were modeled by spherical Gaussian functions [16,17]; nonelectrostatic contributions to the solvation free energy were disregarded at this stage; these terms were accounted for in single-point PCM calculations (at the PCM geometries) employing radii and nonelectrostatic terms of the SMD solvation model [18]. Vibrational-rotational contributions to the free energy were also computed. For the preparation of IR spectra, harmonic frequencies were scaled by a factor of 0.9547 [19] and Gaussian line width of 20 cm^−1^ was used.

Ultraviolet–visible spectra of the main species were computed in vacuo or in solution using the time-dependent density functional theory (TD-DFT) approach [20,21,22,23,24], with the PBE0 functional and the 6-311++G(2d,2p) basis set. To produce graphs, transitions below 5.6 eV were selected, and an arbitrary Gaussian line width of 0.25 eV was imposed; the spectra were finally converted to a wavelength scale.

## 3. Results and Discussion

For the purposes of this study, several different oxidation states were considered, namely: fully reduced (KP-Red), one-electron oxidation (KP-1e, as representative open shell state) and KP-2e, KP-4e, KP-6e and KP-8e as closed shell states.

The generation of the relevant starting structures is straightforward for the KP-Red and KP-1e states. However, for the higher oxidation states explored, the number of possible tautomeric species can become quite significant. A simple procedure to generate them would consist in defining the number of heteroatom-bound hydrogens that corresponds to the target oxidation state (e.g., 10 hydrogens for KP-2e, 8 hydrogens for KP-4e, etc.), and placing them in all possible different ways on the 12 available positions of the porphyrin skeleton (i.e., N1, O5, and O6 for each of the four rings). However, this procedure would not distinguish between closed-shell and open-shell species: these latter are predictably characterized by lower stabilities, and were therefore disregarded in the present exploration. Therefore, a systematic procedure for the generation of relevant closed-shell tautomers was devised, based on combining individual DHI units at the following oxidation stages: (i) fully reduced units, with dangling single bonds at the 2- and 7-position (one type); (ii) one-electron oxidized units, with a dangling double bond at the 2-position and a dangling single bonds at the 7-position (two types), or vice versa (two types); (iii) units at the two-electron oxidation level, with dangling single bonds at the 2- and 7-position (three types, i.e., *o*-quinone, quinone methide, or quinone-imine forms), or else with dangling double bonds at both 2- and 7-position (one type). To build up staring structures for the tetrameric porphyrin system, four building blocks were selected in such a way as to match the desired overall oxidation stage of the porphyrin system, and were combined in all possible ways compatible with the individual connectivity requirements; the resulting pool of structures was examined, and duplicates were discarded. For each tautomer, at least two conformers were generated.

To facilitate discussion and comparison among structures at different oxidation stages, an ad hoc naming scheme was devised, whereby the four individual DHI units of the porphyrin system are labeled as “a”, “b”, “c”, and “d” in the sequence of the 2,7′-connectivities; moreover, mobile hydrogens that have been removed on each unit are explicitly indicated: thus, for example, a four-electron oxidized system featuring an *o*-quinone type unit bound through a 2,7′-single bond to a quinone methide unit, would be dubbed “a56_b16_c0_d0”.

In a preliminary screening, presented in a separate paper, it was found that of the various oxidation states accessible to KP, the six-electron state was relatively more stable [25]. Herein, we provide a detailed account of the structural and spectral properties of KP at the aforementioned oxidation states in order to draw possible structure–property relationships that may allow prediction of functionality and prompt the design of KP functional systems for specific applications.

The general computational frame adopted in this study to investigate energies, structures and spectroscopic properties of the various species has proved reliable in several previous investigations of DHI-related oligomers [26,27,28,29]. However, in order to confirm the reliability in the specific case of KP, we carried out some ad hoc validation tests, which are reported as Supplementary Materials. First, we compared the relative energies of the main tautomers/conformers of KP-4e (neutral form in vacuo) at the PBE0 and B3LYP level [30], using different basis sets, and we fund quite good reproducibility. Analogous calculations were carried out on the main neutral species in all different oxidation states in vacuo; using this set of data, we then recomputed and compared electronic energy changes for disproportionation processes involving KP (see below, Section 3.7). Again, the results appeared rather robust with respect to functional/basis set choice.

A validation of the computed UV–Vis data was sought by comparing TD-PBE0 vs. TD-B3LYP for two related nonoxygenated porphyrin systems that have been synthesized and characterized by Nakamura and colleagues [31,32]. A similar comparison was extended to the high-wavelength electric dipole-allowed transitions of porphin freebase, and of magnesium porphin. TD-B3LYP calculations were also performed on the most significant tautomers/conformers (neutral forms) of KP in all different oxidation states, and the resulting computed spectra were found to resemble closely their TD-PBE0 counterparts. In this connection it is also worth noting that a recent study employing a post-Hartree–Fock approach (CC2) [33] to investigate the onset of electronic absorption spectra of DHI oligomers reported an overall fairly good agreement with literature TD-DFT results.

### 3.1. The Fully Reduced State (KP-Red)

Table 1, Table 2 and Table 3 report structures, energies and relative stabilities of tautomers/conformers in the fully reduced state.

The neutral form has *S*_4_ symmetry both in vacuo and in water; alternative symmetries, including *C*_4_ and *C*_4h_, correspond to high-order saddle points, and at any rate are much higher in energy. In principle, many conformational possibilities arise in connection with the different orientations that can be adopted by the OH groups. However, studies on DHI [34] have shown that both OH groups are coplanar with the indole ring, and that “closed” geometries (i.e., those featuring an intramolecular H-bond between O5–H and O6–H) are significantly more stable than the corresponding “open” form. Of the two alternative “closed” forms, the one in which both OH bonds point in the opposite direction with respect to nitrogen is marginally more stable, and has been adopted throughout. Of course, “closed” geometries are invariably preferred when one of the oxygen centers bears a negative charge or a radical.

The preferred site for deprotonation of KP-Red is predicted to be O6. Based on comparison of ∆*G*_SMD,RRHO_ values with those of a number of reference acid/base pairs of the AH/A^−^ type, the p*K*_a_ is estimated around 8.2.

**Figure 1:**
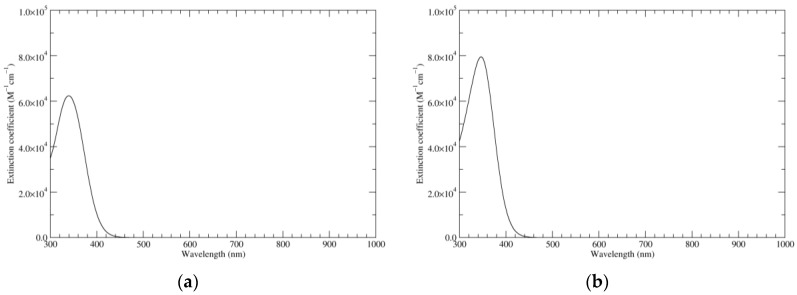
Computed ultraviolet–visible (UV–Vis) spectra of the most significant tautomers/conformers in the reduced state. (**a**) Neutral form in vacuo, a0_b0_c0_d0, *S*_4_. (**b**) Neutral form in water, a0_b0_c0_d0, *S*_4_.

Ultraviolet–visible spectra of the most significant species of KP-Red were computed at the TD-PBE0 level both in vacuo and in water (PCM) (Figure 1). Overall, they show reasonable similarity to the spectrum computed at the same level for the fully reduced form of 2,7′-DHI dimer: a moderate bathochromic shift of ca. 10 nm in the spectrum of KP-Red could reflect the influence of additional interring conjugation, but also of a geometric constrain imposing s-*trans* conformation for N–C2–C7′–C6′ interring dihedrals. The rather large bandwidth reflects convolution of several different individual electronic transitions: the longest-wavelength contribution, at 372 nm (in vacuo), is HOMO–LUMO in character and is characterized by a moderate oscillator strength (*f* = 0.07); more intense transitions connect degenerate HOMO−1/HOMO−2 orbitals to the LUMO (357 nm, *f* = 0.36), and the HOMO to degenerate LUMO+1 and LUMO+2 orbitals (327 nm, *f* = 0.38).

**Figure 2:**
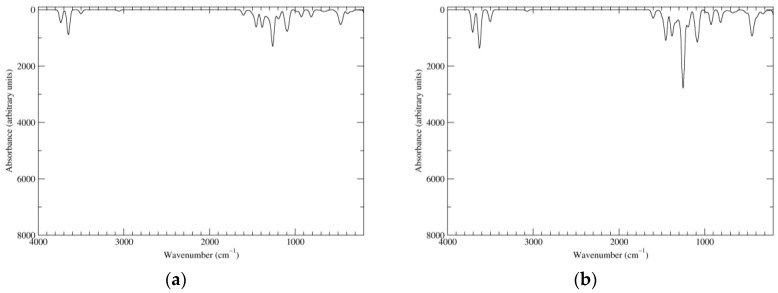
Computed infrared (IR) spectra of the most significant tautomers/conformers in the reduced state. (**a**) Neutral form in vacuo, a0_b0_c0_d0, *S*_4_. (**b**) Neutral form in water, a0_b0_c0_d0, *S*_4_.

Infrared spectra of the main species are displayed in Figure 2 (Raman spectra have been reported in [25] and will not be discussed here). The spectrum of the reduced species will be useful as a reference point for the discussion of the richer spectra of higher oxidation states.

### 3.2. The One-Electron Oxidation State (KP-1e)

Table 4, Table 5 and Table 6 show structures, energies and relative stabilities of the most stable tautomers/conformers in the one-electron oxidation state, i.e., those within 10 kcal mol^−1^ from the corresponding absolute minimum (complete lists are reported as Supplementary Materials).

While the full *S*_4_ symmetry of the reduced state is unavoidably lost, the overall shape of the molecules is not drastically altered. The radical center is preferentially located at an O6 position, again in close parallel with the case of DHI. Deprotonation is predicted to occur on the O6 position of an adjacent ring, with a p*K*_a_ of around 6.2, i.e., more than one p*K* unit lower with respect to value computed at the same level for the deprotonation of an O6-centered DHI radical. In the same vein, the a56_b0_c0_d0 tautomer, in which both proton and hydrogen atom are removed from the same indole unit, is over 11 kcal mol^−1^ higher in energy than the absolute minimum. Clearly, the possibility to distribute charge and spin over more than one ring favors deprotonation; at the same time, electron delocalization is maximized by the involvement of adjacent indole rings.

**Figure 3:**
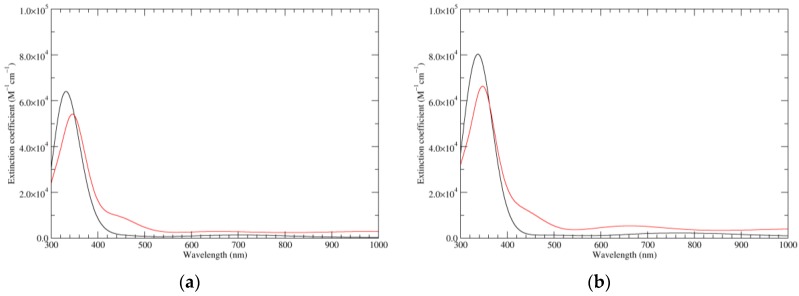
Computed UV–Vis spectra of the most significant tautomers/conformers in the one-electron oxidation state. (**a**) Neutral form in vacuo: black line, a5_b0_c0_d0, *C*_1_, conf1; red line, a6_b0_c0_d0, *C*_1_, conf1. (**b**) Neutral form in water: black line, a5_b0_c0_d0, *C*_1_, conf1; red line, a6_b0_c0_d0 *C*_1_, conf1.

Computed UV–Vis spectra of the most significant neutral tautomers of KP-1e are shown in Figure 3. Detailed analysis of the UV–Vis spectra of such open-shell species is difficult, and can be complicated by intrinsic limitations of the TD-DFT model. However, in the case of a6_b0_c0_d0, the predominant tautomer both in vacuo and in water, one can observe that the singly occupied molecular orbital (SOMO) is mostly localized on the “a” ring, with some delocalization on the “d” ring, and to a smaller extent also on the “b” ring. Such limited delocalization is reflected in the appearance of the spectra, in which high-wavelength, low-intensity bands corresponding mostly to transitions into the SOMO are superimposed on a trace resembling the spectrum of KP-Red.

### 3.3. The Two-Electron Oxidation State (KP-2e)

The most significant structures corresponding to two-electron oxidation state are listed in Table 7, Table 8 and Table 9.

**Figure 4:**
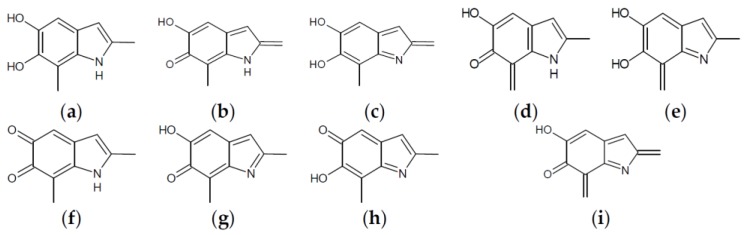
Building blocks for generation of staring structures of closed-shell tautomers of Kaxiras’s porphyrin. (**a**) Fully reduced units. (**b**–**e**) One-electron oxidized units. (**f**–**i**) Two-electron oxidized units.

Already at this oxidation stage, and even restricting the attention to closed-shall forms, the number of alternative tautomers starts to increase significantly. There are three ways to localize the oxidation on a single ring (Figure 4, structures f–h), giving rise to a15_b0_c0_d0, a16_b0_c0_d0, or a56_b0_c0_d0 tautomers; moreover, four tautomers featuring an inter-ring double bond (a1_b1_c0_d0, a1_b6_c0_d0, a6_b1_c0_d0, a6_b6_c0_d0) can be generated by combined two one-electron oxidized units (Figure 4, structures b–e). However, most of these structures are energetically unviable. In practice, the a6_b6_c0_d0 tautomer, featuring a quinone methide structure extended over two adjacent indole rings, is predicted to prevail largely both in vacuo and in water. Thus, the tendency to maximize electron delocalization by involving adjacent indole rings is confirmed in the two-electron oxidation state. The double bond character of the C2 (ring a)-C7 (ring b) connectivity is reflected in the bond length of 1.396 Å (in vacuo; to compare with the other interring bonds of 1.542, 1.457 and 1.447 Å).

Deprotonation of the system is predicted to occur from O5–H of the quinone methide ring, with a p*K*_a_ around 6.1, and slightly less probably from the NH (p*K*_a_ ca. 6.4).

**Figure 5:**
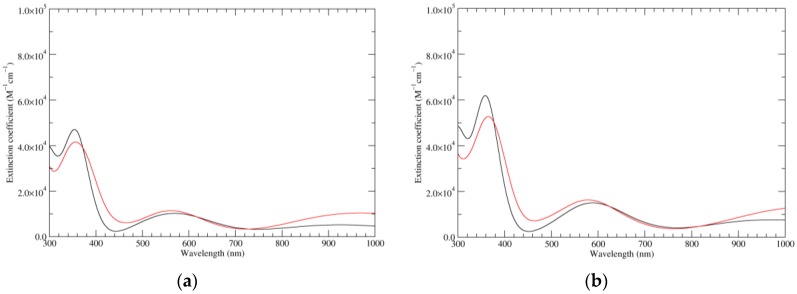
Computed UV–Vis spectra of the most significant tautomers/conformers in the two-electrons oxidation state. (**a**) Neutral form in vacuo: black line, a16_b0_c0_d0, *C*_1_, conf1; red line, a6_b6_c0_d0, *C*_1_, conf1. (**b**) Neutral form in water: black line, a16_b0_c0_d0, *C*_1_, conf1; red line, a6_b6_c0_d0, *C*_1_, conf1.

The two significant tautomeric forms of KP-2e, a16_b0_c0_d0 and a6_b6_c0_d0, display quite similar spectra (Figure 5). Examination of the underlying orbital structure shows that in both cases the high-wavelength region corresponds to an ordered series of several transitions starting from HOMO, HOMO−1, HOMO−2, … and ending in the LUMO. This latter orbital is rather similar in the two species; however, correspondence of the other orbitals involved is only partial, and moreover some inversions in energy ordering occur. The effect of solvation is also predicted to be comparable in the two cases.

**Figure 6:**
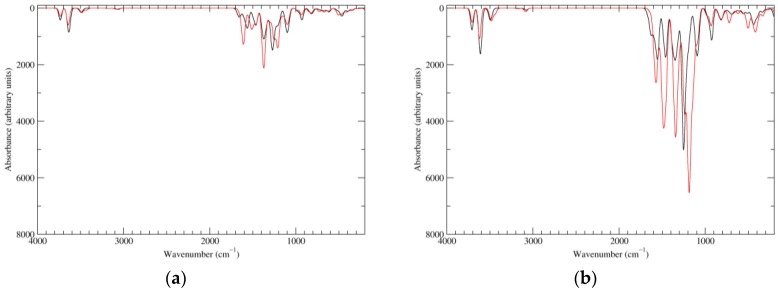
Computed IR spectra of the most significant tautomers/conformers in the two-electrons oxidation state. (**a**) Neutral form in vacuo: black line, a16_b0_c0_d0, *C*_1_, conf1; red line, a6_b6_c0_d0, *C*_1_, conf1. (**b**) Neutral form in water: black line, a16_b0_c0_d0, *C*_1_, conf1; red line, a6_b6_c0_d0, *C*_1_, conf1.

Figure 6 shows the computed IR spectra of the two main tautomers. With respect to the spectrum of KP-Red, the presence of new features in the carbonyl region is apparent; moreover, these are rather different for the two tautomers. Visual examination of the individual normal modes shows that for a16_b0_c0_d0, a quinone methide localized on ring a, the peak at 1655 cm^−1^ (in vacuo) correspond in essence to an antiphase stretching of the C4–C5 double bond and of the C6–O carbonyl, accompanied however by sizeable deformations of the C5–O–H moiety; the peak at 1562 cm^−1^ can be described as a C6–O carbonyl stretching. In the case of a6_b6_c0_d0, a quinone methide extended over rings a and b, the peak at about 1609 cm^−1^ convolutes two main transitions, separated by some 20 cm^−1^. The high-wavenumber component corresponds to in-phase stretching of the C6–O (ring a) and C6–O (ring b) carbonyls, which are conjugated through a system of three double bonds; the low-wavenumber component (of higher intensity) represents the corresponding antiphase stretching.

### 3.4. The Four-Electron Oxidation State (KP-4e)

At this stage, structural complexity is remarkable: 49 closed-shell, neutral tautomers were generated (each in at least two different staring conformation) and fully geometry-optimized. As a rule, each of these structures features many nonequivalent deprotonation sites: an exploration of the monoanionic forms would therefore be extremely challenging, and was not attempted in this case, nor for any of the higher oxidation states.

At this oxidation level, the formation of π-conjugated cyclic systems extending over the four indole units becomes possible, and is obtained in the following tautomers: a1_b1_c1_d1 (28 π-electrons); a1_b1_c1_d6 (26 π-electrons); a1_b1_c6_d6 and a1_b6_c1_d6 (24 π-electrons); a1_b6_c6_d6 (22 π-electrons); a6_b6_c6_d6 (20 π-electrons). The relative energies of these species vary over a large range (see Supplementary Materials); even restricting the attention to species that comply with the 4*n* + 2 rule, a1_b1_c1_d6 is 67.6 kcal mol^−1^ above the absolute minimum (in vacuo). Conversely, a6_b6_c6_d6, an antiaromatic system, has a relative energy of just 3.8 kcal mol^−1^. Overall, it appears that aromatic character by itself is not a major predictor of tautomer stability.

Among all neutral tautomers, six were within 10 kcal mol^−1^ from the absolute minimum in vacuo (and respectively eight in water). These are listed in Table 10 and Table 11.

As a matter of fact, a subset of structures is quite close in energy. Tautomers a16_b6_c6_d0 and a6_b6_c16_d0 combine the extended quinone methide structure described for the two-electron oxidation state, with a single two-electron oxidized ring in a localized quinone methide tautomeric arrangement. A new motif appears in a6_b16_c6_d0, in which a two-electron oxidized ring (Figure 4, structure **i**) is interposed by means of double bonds between two one-electron oxidized building blocks, with formation of an extended system formally delocalized over three indole units. The character of the interring double bonds is confirmed by the lengths of 1.395 Å for C2 (ring a)–C7 (ring b), and 1.400 Å for C2 (ring b)–C7 (ring c) (in vacuo; the interring single bonds have instead lengths of 1.450 and 1.442 Å). In water, the pool of predominant structures is enriched by still another tautomer, a6_b6_c6_d6, formed by a duplication of the two-ring, two-electron building block described above. As a matter of fact, even starting from higher *S*_4_ or *C*_4_ symmetries, the system spontaneously reverts to the *C*_2_ symmetry. Bond lengths reflect the alternation of interring double (1.390 Å, in vacuo) and single bonds (1.448 Å).

**Figure 7:**
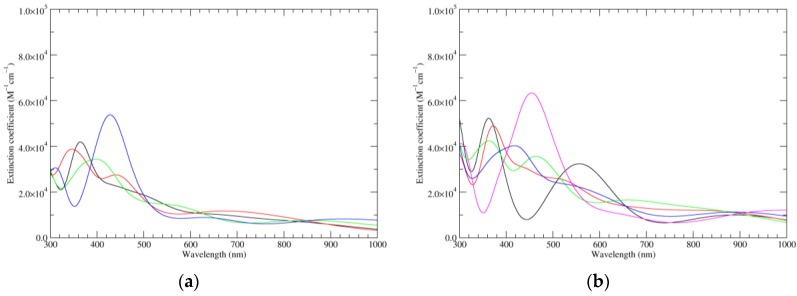
Computed UV–Vis spectra of the most significant tautomers/conformers in the four-electrons oxidation state. (**a**) Neutral form in vacuo: black line, a16_b6_c6_d0, *C*_1_, conf1; red line, a6_b16_c6_d0, *C*_1_, conf1; green line, a6_b6_c16_d0, *C*_1_, conf1; blue line, a6_b6_c6_d6, *C*_2_, conf1. (**b**) Neutral form in water: black line, a16_b0_c16_d0, *C*_2_, conf1; red line, a16_b6_c6_d0, *C*_1_, conf1; green line, a6_b16_c6_d0, *C*_1_, conf1; blue line, a6_b6_c16_d0, *C*_1_, conf1; magenta line, a6_b6_c6_d6, *C*_2_, conf1.

The significant degree of structural variation of the predominant tautomers is reflected in the computed UV–Vis spectra (Figure 7). Concerning the high-wavelength region, however, some general trends can be identified. First, the seeming broadband absorptions can be traced back to the partial overlap of a large number of weakly allowed transitions. Moreover, the highest wavelength transition is invariably HOMO–LUMO in character. The transitions that follow display a rather strong orbital mixing: a reasonably clear series of HOMO−1, HOMO−2, … –LUMO transitions is interrupted by several transitions into the LUMO+1 orbital, the exact ordering depending on the specific tautomer.

**Figure 8:**
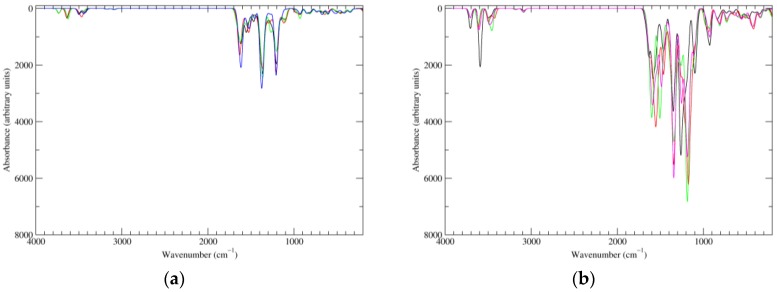
Computed IR spectra of the most significant tautomers/conformers in the four-electrons oxidation state. (**a**) Neutral form in vacuo: black line, a16_b6_c6_d0, *C*_1_, conf1; red line, a6_b16_c6_d0, *C*_1_, conf1; green line, a6_b6_c16_d0, *C*_1_, conf1; blue line, a6_b6_c6_d6, *C*_2_, conf1. (**b**) Neutral form in water: black line, a16_b0_c16_d0, *C*_2_, conf1; red line, a16_b6_c6_d0, *C*_1_, conf1; green line, a6_b16_c6_d0, *C*_1_, conf1; blue line, a6_b6_c16_d0, *C*_1_, conf1; magenta line, a6_b6_c6_d6, *C*_2_, conf1.

Figure 8 reports the computed IR spectra of the main tautomers. Concentrating the analysis on the carbonyl/double bond stretching region, one can observe that a16_b6_c6_d0 and a6_b6_c16_d0 display similar features, with main peaks at about 1620 and 1555 cm^−1^ (in vacuo): this reflects structural similarity, both tautomers consisting of localized quinone methide, and an extended quinone methide involving the two following (a16_b6_c6_d0) or preceding (a6_b6_c16_d0) rings. These features are shifted to ca. 1645 and 1527 cm^−1^ in a6_b16_c6_d0, in which inter-ring double bonds form a conjugated system extended over three rings. Inspection of the underlying normal modes shows that the high-energy band results from overlap of several transitions; however, the two main components correspond to motions centered in ring b. The higher-energy component, an antiphase stretching of the C4–C5 double bond and of the C6–O carbonyl, with concomitant deformations of the C5–O–H moiety, is quite similar to that described for the isolated quinone methide (a16_b0_c0_d0 at the two-electron oxidation stage). The lower-energy, more intense contribution involves antiphase stretching of the C6–O (ring a) and C6–O (ring c) carbonyls. Finally, in a6_b6_c6_d6, in which two equivalent two-ring conjugated systems exist, main peaks are at 1616 and 1523 cm^−1^: although several individual transitions are hidden under the 1616 cm^−1^ peak, the two most intense ones can be described as in-phase (higher energy) and antiphase (lower energy) stretching of the C6–O (ring a) and C6–O (ring b) carbonyls; of course, corresponding motions occur on the symmetry-related rings b and d, with relative phases dictated by the B symmetry species of both modes.

### 3.5. The Six-Electron Oxidation State (KP-6e)

Seventy-four tautomers of KP-6e were examined. Extended conjugated cyclic systems are possible in the following cases: a16_b16_c1_d1 and a16_b1_c16_d1 (22 π-electrons); a16_b16_c1_d6, a16_b16_c6_d1 and a16_b1_c16_d6 (20 π-electrons); a16_b16_c6_d6 and a16_b6_c16_d6 (18 π-electrons). The 22 π-electrons systems, although predictably aromatic, are high in energy (ca. 100 kcal mol^−1^ above the minimum; see Supplementary Materials), even more than the 20 π-electrons tautomers (relative energies of ca. 45 kcal mol^−1^). However, the two 18 π-electrons tautomers are indeed the two most stable isomers identified at this oxidation stage. Of them, a16_b6_c16_d6 is strongly favored both in vacuo and in water; a16_b16_c6_d6 is separated from the absolute minimum by ca. 8–9 kcal mol^−1^ (Table 12 and Table 13).

**Scheme 2:**
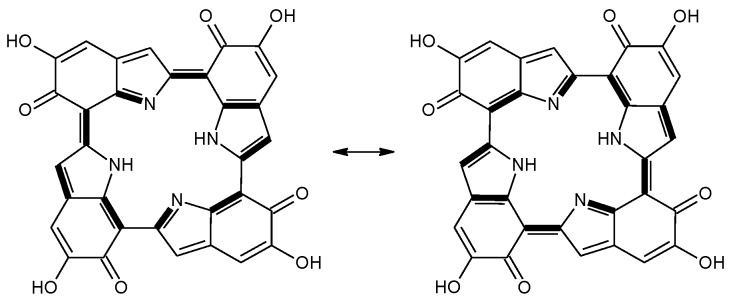
a16_b6_c16_d6 as a hybrid between two equivalent contributing structures. The pattern of conjugated double bonds forming an 18-electron aromatic system is highlighted in bold.

This situation may reflect in part an optimal arrangement of NH···N hydrogen bonds in the core of the porphyrin ring; however, one should emphasize again the aromatic character of a16_b6_c16_d6 (nine conjugated double bonds in an uninterrupted cyclic arrangement; 18 π-electron system). The molecule features *C*_2_ symmetry: this is brought out clearly by a representation in terms of resonance structures (Scheme 2). The partial double bond character of the interring bonds is reflected in short lengths and in a moderate alternation (in vacuo, C2 (ring a))–C7 (ring b) and C2 (ring c))–C7 (ring d) have a length of 1.417 Å, while C2 (ring b))–C7 (ring c) and C2 (ring d))–C7 (ring a) are 1.409 Å long). The structure is much closer to planarity than in the lower oxidation states, reaching an approximate *C*_2h_ symmetry.

Taken together, the two factors hinted above (favorable hydrogen bond pattern and extended aromatic π-electron system) confer a remarkable stability to the structure, both with respect to other tautomeric arrangements at the same oxidation level, and in comparison to other oxidation states (see below).

**Figure 9:**
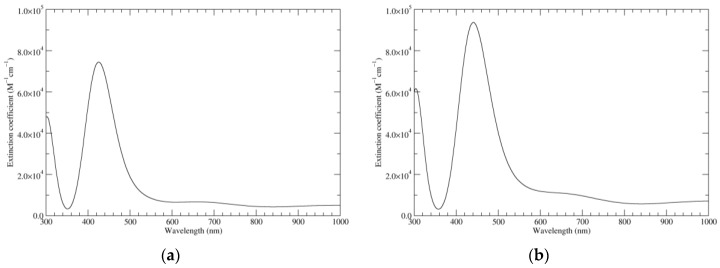
Computed UV–Vis spectra of the most significant tautomers/conformers in the six-electron oxidation state. (**a**) Neutral form in vacuo, a16_b6_c16_d6, *C*_2_, conf1. (**b**) Neutral form in water, a16_b6_c16_d6, *C*_2_, conf1.

Figure 9 displays the computed UV–Vis spectra of a16_b6_c16_d6. A remarkable extinction coefficient is predicted for the band at ca. 420 nm, higher than in any of the other oxidation states. On account of the symmetry of the species, several electronic transitions are forbidden; many more have low intensity, including notably the highest wavelength transition (1080 nm, *f* = 0.03, mostly HOMO–LUMO in character). In the region above 500 nm, the only other transitions with non-null oscillator strength are the following (in vacuo): 988 nm (*f* = 0.06, HOMO–LUMO+1); 729 nm (*f* = 0.02, HOMO−1–LUMO+1); 678 nm (*f* = 0.09, HOMO−1–LUMO); 569 nm (*f* = 0.05, HOMO−4–LUMO); and 520 nm (*f* = 0.04, HOMO−4–LUMO+1). Orbital mixing is significant: in particular, whenever a given excited state contains a LUMO component, the LUMO+1 is invariably strongly admixed, and vice versa. This behavior is strongly reminiscent of the orbital structure of classical porphyrins, in which, depending on the molecular symmetry, LUMO and LUMO+1 are either exactly or very closely degenerate, and enter as a pair in the composition of the spectroscopically relevant excited states. To be sure, the computed spectrum of a16_b6_c16_d6 displays typical signatures of a porphyrin: the intense peak around 420 nm can be recognized as a Soret (B) band, while the low-intensity peaks at higher wavelength can be classified, at least in part, as Q bands [35]. The 420 nm band corresponds to two distinct transitions, at 431 and 418 nm (*f* = 0.56 and 0.79, respectively): this splitting can be traced back to the lower symmetry of a16_b6_c16_d6 with respect to typical metalloporphyrin complexes endowed with more or less exact *D*_4h_ symmetry—as a matter of fact, a similar situation is observed in the less symmetric porphyrin free base (*D*_2h_ in the parent heterocycle).

As it is well known [36], vibronic features contribute significantly to the appearance of experimental UV–Vis spectra of porphyrins. It is, therefore, expedient to recall that the computational model we adopted does not include any such effects, not even a reproduction of the vibrational structure of the allowed electronic transitions. Much progress has been done recently towards automation and optimization of such computations [37], so that they are becoming feasible even for large molecules in condensed phases; however, they remain by no means trivial. Certainly, the possible involvement of such physical phenomena should be kept in mind if experimental data on the individual KPs become available.

In order to better characterize the nature of the underlying electronic transitions, TD-DFT calculations (Table 14) were repeated on a16_b6_c16_d6 in its *C*_2h_-symmetric structure, which corresponds to a first-order saddle point. Use of a more symmetrical structure facilitates classification of the orbitals and of the electronic states in terms of symmetry species; moreover, in the present instance changes in transition wavelengths and intensities are negligible (a more complete version of Table 14, including orbital symmetry labels, is available as Supplementary Materials, alongside corresponding data for the *C*_2_-symmetric structure).

Visual inspection of the molecular orbitals (available as Supplementary Materials) confirms that the LUMO and LUMO+1 b_g_ orbitals correspond rather well to the degenerate pair of unoccupied e_g_ orbitals that are involved in the UV–Vis transitions of typical metalloporphyrins. However, the situation becomes more intricate when the occupied orbitals are examined: in the present case, HOMO, HOMO−1, HOMO−4 and HOMO−5 all belong to the same A_u_ symmetry species, and mix heavily in the transitions leading to the “Soret” band. This complicates a straightforward interpretation in terms of the classical Gouterman “four-orbital” model [38].

**Figure 10:**
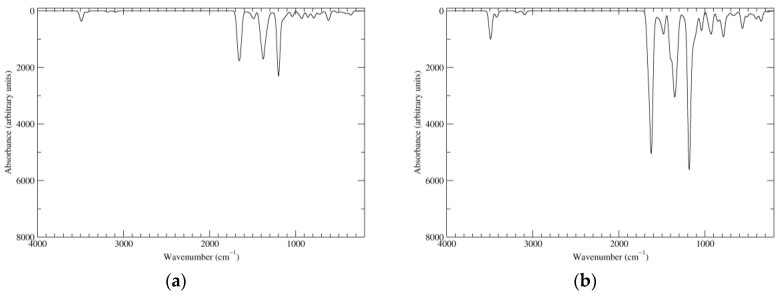
Computed IR spectra of the most significant tautomers/conformers in the six-electrons oxidation state. (**a**) Neutral form in vacuo, a16_b6_c16_d6, *C*_2_, conf1. (**b**) Neutral form in water, a16_b6_c16_d6, *C*_2_, conf1.

Simulated IR spectra of a16_b6_c16_d6 are shown in Figure 10. Main peaks in the carbonyl/double bond stretching region are predicted at about 1655 and 1490 cm^−1^ (in vacuo). Four transitions of comparable intensity underlie the 1655 cm^−1^ peak. The highest energy component, at 1670 cm^−1^, corresponds to the usual antiphase stretching of the C4–C5 double bond and of the C6–O carbonyl (with concomitant deformations of the C5–O–H moiety), centered on the “b” indole unit (and of course on the symmetry-related “d” indole unit: all four modes belong to the B symmetry species). The transition at 1668 cm^−1^ is similar, but centered on ring a. Conversely, the 1644 cm^−1^ transition corresponds to an in-phase stretching of C4–C5 and C6–O of ring a, with a counterpart at 1639 cm^−1^ for the analogous motion on ring b. The peak at ca. 1490 cm^−1^ is composed by two main transitions, which involve notably interring bonds; in particular, stretching contributions of C7 (ring b))–C7a (ring b) and of C2 (ring b)–C7 (ring c) are prominent in the high-energy (1509 cm^−1^) component; C2 (ring b)–C7 (ring c) is also well present in the low-energy (1482 cm^−1^) counterpart, which however involves a substantial contribution from in-plane bending of the N–H. Again, motions in the symmetry-related rings are dictated by the B symmetry species of both modes.

### 3.6. The Eight-Electron Oxidation State (KP-8e)

The highest oxidation state that has been examined in this study corresponds to removing eight electrons and eight protons from the fully reduced tetramer. At such high oxidation states, the number of possible tautomers starts to decrease: 24 tautomers were generated, and several conformers were optimized as usual. A fully π-conjugated cyclic system extending over the four rings is present in a16_b16_c16_d16; however, with 16 π-electrons the system is predicted to be antiaromatic, and at any rate it is high in energy (ca. 36 kcal mol^−1^ above the minimum). Energetically significant species are collected in Table 15 and Table 16.

As it turns out, a single form, namely a16_b56_c16_d56, predominates largely both in vacuo and in water. Here all rings are at the same two-electron oxidation state, and localized quinone methide units alternate with localized *o*-quinones so as to create an optimal pattern of transannular NH···N hydrogen bonds. However, porphyrin-type aromaticity is lost at this stage. The structure is *C*_2_-symmetric, but less close to planarity that the main tautomer of KP-6e; interring bonds are longer (1.419 and 1.434 Å, in vacuo).

**Figure 11:**
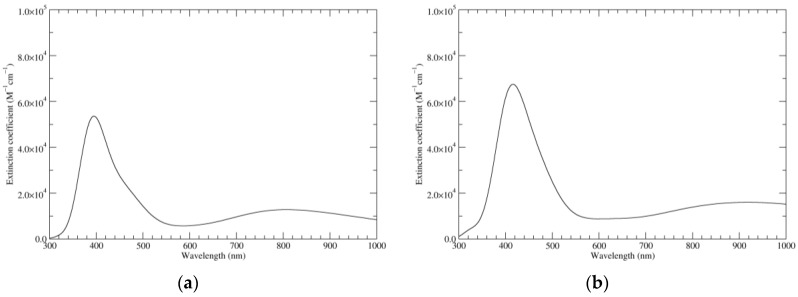
Computed UV–Vis spectra of the most significant tautomers/conformers in the eight-electrons oxidation state. (**a**) Neutral form in vacuo, a16_b56_c16_d56, *C*_2_, conf1. (**b**) Neutral form in water, a16_b56_c16_d56, *C*_2_, conf1.

The electronic spectrum (Figure 11) resembles roughly that of KP-6e; however, the main peak is shifted hypso- and hypochromically. In the low-energy region, transitions of significant intensity (in vacuo) are predicted at 985 (*f* = 0.10; mostly HOMO–LUMO), 788 (*f* = 0.18; main components HOMO–LUMO and HOMO−1–LUMO+1), and 586 nm (*f* = 0.05; HOMO−1–LUMO+2).

**Figure 12:**
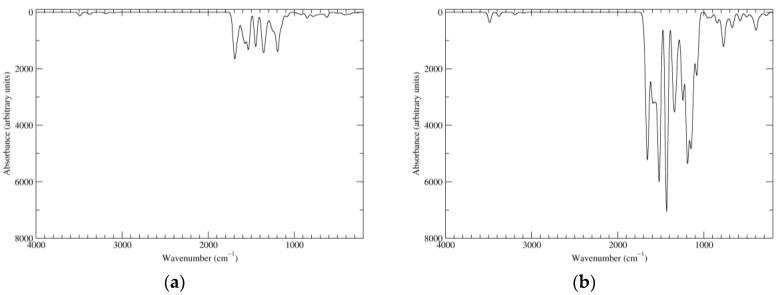
Computed IR spectra of the most significant tautomers/conformers in the eight-electrons oxidation state. (**a**) Neutral form in vacuo, a16_b56_c16_d56, *C*_2_, conf1. (**b**) Neutral form in water, a16_b56_c16_d56, *C*_2_, conf1.

The IR spectrum of a16_b56_c16_d56 (Figure 12) shows in the carbonyl region a major peak (1690 cm^−1^ in vacuo, 1657 cm^−1^ in water). In terms of normal modes, this corresponds to a stretching motion of the *o*-quinone carbonyl groups, which is in phase for what concerns the two carbonyls on the same ring, and antiphase for what concerns the symmetry-related b and d rings (B symmetry species). The mode in which the two carbonyls on a same ring move in antiphase is slightly higher in energy, but has much lower intensity. Modes centered on the quinone methide rings (a and c) account for the low-wavenumber shoulder of the peak. Major contributing transitions can also be identified for the bands at 1575–1535 cm^−1^. The principal contribution to the high-energy band comes from a mode involving in-phase stretching of C7 (ring a)–C7a (ring a) and of C2 (ring a)–C7 (ring b); bending of the NH group is prominent (as usual, motions of the other rings account for a B symmetry species). Analogous stretching motions of C7 (ring a)–C7a (ring a), C7 (ring b)–C7a (ring b), C2 (ring b)–C7 (ring c) is involved in the low-energy transition; however, the NH bending contribution is much smaller in this case.

### 3.7. HOMO–LUMO Gaps

Trends in HOMO and LUMO energies and in the resulting HOMO–LUMO gaps are illustrated in Table 17 for the main neutral species in each oxidation state.

As expected, the oxidation state plays the largest role; however, even within the range of energetically significant species, changes connected to the specific tautomer/conformer are sometimes significant. HOMO energies display a rather regular decrease as a function of oxidation state; by contrast, LUMO energies shows larger and less regular changes, with a marked downward step of ca. 2.0 eV between KP-Red/KP-1e and KP-2e. The combined effect of these two factors is that the HOMO–LUMO gap changes significantly along the series, reaching a minimum for KP-4e (1.108 eV for a6_b6_c6_d6; ca. 1.4 eV for the other tautomers). Values for KP-2e, KP-6e and KP-8e are in the range 1.5–1.8 eV. Solvent effects on the HOMO–LUMO gap are significant in some cases; however, the overall picture is not substantially modified.

### 3.8. Disproportionation Equilibria

Table 18 lists reaction free energies computed in vacuo and in water for several disproportionation processes involving KP at different oxidation levels. For ease of comparison, values referred to the conversion of one mole of reactant are also listed (this would entail use of fractional stoichiometric coefficients for the products).

In vacuo, the predicted behavior is very clear: all forms at oxidation stages intermediate between the fully reduced and the six-electron oxidized level are predicted to undergo spontaneous disproportionation, leading eventually to formation of KP-Red and KP-6e.

KP-8e corresponds to the highest oxidation level computed in this study; therefore, its disproportionation could not be modeled. However, disproportionation of KP-6e to KP-Red and KP-8e is so unfavorable (i.e., the opposite comproportionation process is so favorable) that KP-8e could not coexist with any substantial amounts of forms below the six-electron oxidation level; therefore, it could become significant only under specific oxidizing conditions.

**Figure 13:**
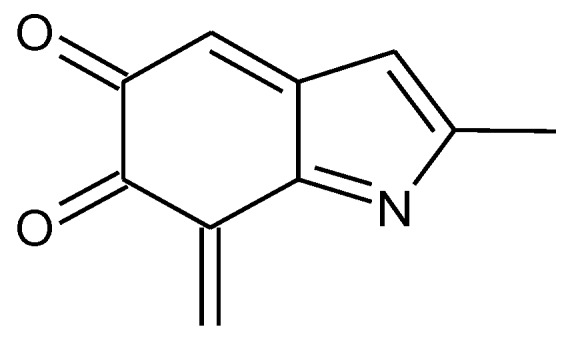
A three-electron oxidized building block, which would be needed for the generation of closed-shell tautomers at oxidation levels above KP-8e.

Moreover, oxidation levels above KP-8e can be expected to be even less stable. For example, a closed-shell structure for KP-10e requires that at least two indole units reach the three-electron oxidation state. The remaining two units can then contribute a two-electron oxidation level each; in alternative, one of them can be at the one-electron oxidation level, and the other one again at the three-electron level. There is just one way to write a three-electron oxidized building block (Figure 13). Intrinsically, this would be a high-energy site: as a matter of fact, this type of building block was not employed in the generation of starting structures of lower oxidation levels. Moreover, there is no way to combine two such units with two two-electron oxidized building blocks (see Figure 4), nor any way to arrange three three-electron units with a one-electron building block. Analogous considerations hold for KP-12e.

The one-electron oxidation free radical species displays a remarkably low tendency to disproportionation: however, the process is still predicted to be favorable in vacuo.

In water, disproportionations of KP-6e remain highly endoergonic, but less so than in vacuo. All other disproportionation processes are slightly less favorable in water than in vacuo. In particular, the conversion of KP-2e to KP-Red and KP-4e becomes unfavorable, however, this is compensated by subsequent disproportionation of KP-4e, so that the overall conversion of KP-2e to KP-Red and KP-6e remains spontaneous. By contrast, disproportionation of KP-1e (either to KP-Red and KP-2e, or all the way to KP-Red and KP-6e) is predicted to be (slightly) endoergonic: this would imply that the free radical should be present in appreciable concentrations in partially oxidized mixtures of KP tetramers in aqueous conditions. This could be taken as an indication of a high reactivity of KP under such conditions [39].

More in general, the comparatively small values of reaction free energies in water would allow for co-existence of species at different oxidation stages. This is particularly relevant in view of the predicted variations of UV–Vis absorption spectra as a function of oxidation level, which is characterized by significant shifts in the absorption properties, especially in the low-energy visible end of the spectrum.

## 4. Conclusions

The basic variations in the structural features, chemical stability and optical properties of the DHI-based porphyrin system reported by Kaxiras in 2006 have been investigated in a systematic manner by a DFT approach as a function of main accessible oxidation states. Main results can be summarized as follows:

- As a rule, oxidation of the tetracatechol system results in a loss of stability, with the noticeable exception of the six-electron level and the unexpectedly low tendency of the one-electron oxidation free radical species to disproportionate.
- Oxidation is associated with variable changes in the HOMO–LUMO gaps leading to the development of more or less intense absorption bands in the visible region.
- Tautomerism becomes significant with increasing oxidation states, whereby conjugated systems extending over two or more indole units usually play a major role in determining the preferred tautomeric state.
- The highest stability predicted for the six-electron oxidation state would at least in part reflect porphyrin-type aromaticity imparted to the core tetrapyrrole ring system by oxidation at the catechol centers.

Overall, these results allow us to conclude that the co-existence in the planar cyclic platform of a symmetric tetracatechol oxygenation pattern that can interact with the four central NH centers endows this intriguing tetraindole system with highly tunable optical, redox and electronic properties that are governed by reversible and dynamic interconversion of catechol/semiquinone/ quinone/quinone methide moieties. These results may guide the design of innovative bioinspired optoelectronic materials of potential technological interest.

## Figures and Tables

**Table 1:** KP-Red, neutral form in vacuo.

Tautomer	Conformer ^a^	*E* (Ha) ^b^	*H*_RRHO_ (Ha) ^c^	*G*_RRHO_ (Ha) ^d^
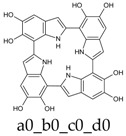	*S*_4_	−2050.155247 (0.0)	−2049.641430 (0.0)	−2049.739870 (0.0)

In parentheses relative energies (kcal mol^−1^) refer to the most stable form identified at the specified level. ^a^ For chiral structures, only one enantiomer is listed. ^b^ Electronic energy. ^c^ Enthalpy computed at 298.15 K within the rigid-rotor/harmonic-oscillator (RRHO) approximation. ^d^ Gibbs free energy computed at 298.15 K within the RRHO approximation.

**Table 2:** KP-Red, neutral form in water.

Tautomer	Conformer ^a^	*G*_PCM_ (Ha) ^b^	*H*_PCM,RRHO_ (Ha) ^c^	*G*_PCM,RRHO_ (Ha) ^d^	*G*_SMD_ (Ha) ^e^	*G*_SMD,RRHO_ (Ha) ^f^
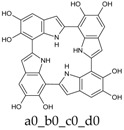	*S*_4_	−2050.191047 (0.0)	−2049.678884 (0.0)	−2049.779144 (0.0)	−2050.220499 (0.0）	−2049.808596 (0.0)

In parentheses relative energies (kcal mol^−1^) refer to the most stable form identified at the specified level. ^a^ For chiral structures, only one enantiomer is listed. ^b^ Electronic energy including electrostatic contributions at the polarizable continuum model (PCM) level. ^c^ Enthalpy computed at 298.15 K within the rigid-rotor/harmonic-oscillator (RRHO) approximation. ^d^ Gibbs free energy computed at 298.15 K within the RRHO approximation. ^e^ Electronic energy including nonelectrostatic terms according to the SMD solvation model. ^f^ *G*_SMD,RRHO_ = *G*_PCM,RRHO_ + *G*_SMD_ − *G*_PCM_.

**Table 3:** KP-Red, monoanionic form in water.

Tautomer	Conformer ^a^	*G*_PCM_ (Ha) ^b^	*H*_PCM,RRHO_ (Ha) ^c^	*G*_PCM,RRHO_ (Ha) ^d^	*G*_SMD_ (Ha) ^e^	*G*_SMD,RRHO_ (Ha) ^f^
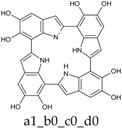	*C*_1_, conf1	−2049.709473 (8.6)	-	-	-	-
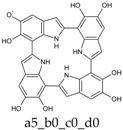	*C*_1_, conf1	−2049.719292 (2.4)	−2049.220534 (2.6)	−2049.319341 (2.4)	−2049.749637 (1.7)	−2049.349686 (1.7)
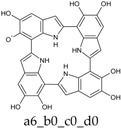	*C*_1_, conf1	−2049.723100 (0.0)	−2049.224673 (0.0)	−2049.323215 (0.0)	−2049.752271 (0.0)	−2049.352386 (0.0)

In parentheses relative energies (kcal mol^−1^) refer to the most stable form identified at the specified level. ^a^ For chiral structures, only one enantiomer is listed. ^b^ Electronic energy including electrostatic contributions at the polarizable continuum model (PCM) level. ^c^ Enthalpy computed at 298.15 K within the rigid-rotor/harmonic-oscillator (RRHO) approximation. ^d^ Gibbs free energy computed at 298.15 K within the RRHO approximation. ^e^ Electronic energy including nonelectrostatic terms according to the SMD solvation model. ^f^ *G*_SMD,RRHO_ = *G*_PCM,RRHO_ + *G*_SMD_ − *G*_PCM_.

**Table 4:** KP-1e, neutral form in vacuo.

Tautomer	Conformer ^a^	*E* (Ha) ^b^	*H*_RRHO_ (Ha) ^c^	*G*_RRHO_ (Ha) ^d^
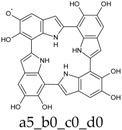	*C*_1_, conf1	−2049.532339 (5.5)	−2049.031222 (5.4)	−2049.129769 (5.2)
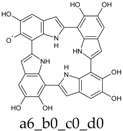	*C*_1_, conf1	−2049.541050 (0.0)	−2049.039878 (0.0)	−2049.138067 (0.0)

In parentheses relative energies (kcal mol^−1^) refer to the most stable form identified at the specified level. ^a^ For chiral structures, only one enantiomer is listed. ^b^ Electronic energy. ^c^ Enthalpy computed at 298.15 K within the rigid-rotor/harmonic-oscillator (RRHO) approximation. ^d^ Gibbs free energy computed at 298.15 K within the RRHO approximation.

**Table 5:** KP-1e, neutral form in water.

Tautomer	Conformer ^a^	*G*_PCM_ (Ha) ^b^	*H*_PCM,RRHO_ (Ha) ^c^	*G*_PCM,RRHO_ (Ha) ^d^	*G*_SMD_ (Ha) ^e^	*G*_SMD,RRHO_ (Ha) ^f^
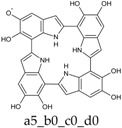	*C*_1_, conf1	−2049.567367 (4.9)	−2049.067779 (4.8)	−2049.167187 (5.1)	−2049.594711 (4.7)	−2049.194531 (4.9)
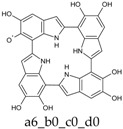	*C*_1_, conf1	−2049.575106 (0.0)	−2049.075489 (0.0)	−2049.175302 (0.0)	−2049.602176 (0.0)	−2049.202372 (0.0)

In parentheses relative energies (kcal mol^−1^) refer to the most stable form identified at the specified level. ^a^ For chiral structures, only one enantiomer is listed. ^b^ Electronic energy including electrostatic contributions at the polarizable continuum model (PCM) level. ^c^ Enthalpy computed at 298.15 K within the rigid-rotor/harmonic-oscillator (RRHO) approximation. ^d^ Gibbs free energy computed at 298.15 K within the RRHO approximation. ^e^ Electronic energy including nonelectrostatic terms according to the SMD solvation model. ^f^ *G*_SMD,RRHO_ = *G*_PCM,RRHO_ + *G*_SMD_ − *G*_PCM_.

**Table 6:** KP-1e, monoanionic form in water.

Tautomer	Conformer ^a^	*G*_PCM_ (Ha) ^b^	*H*_PCM,RRHO_ (Ha)	*G*_PCM,RRHO_ (Ha)	*G*_SMD_ (Ha) ^c^	*G*_SMD,RRHO_ (Ha) ^d^
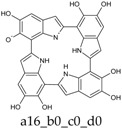	*C*_1_, conf1	−2049.105711 (7.3)	-	-	-	-
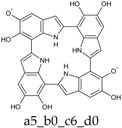	*C*_1_, conf1	−2049.103783 (8.5)	-	-	-	-
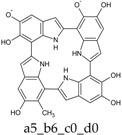	*C*_1_, conf1	−2049.108387 (5.6)	-	-	-	-
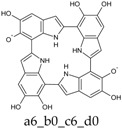	*C*_1_, conf1	−2049.104303 (8.2)	-	-	-	-
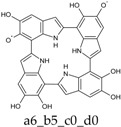	*C*_1_, conf1	−2049.106920 (6.5)	-	-	-	-
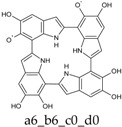	*C*_1_, conf1	−2049.117326 (0.0)	−2048.630807 (0.0)	−2048.728019 (0.0)	−2049.141803 (0.0)	−2048.752496 (0.0)

In parentheses relative energies (kcal mol^−1^) refer to the most stable form identified at the specified level. ^a^ For chiral structures, only one enantiomer is listed. ^b^ Electronic energy including electrostatic contributions at the polarizable continuum model (PCM) level. ^c^ Enthalpy computed at 298.15 K within the rigid-rotor/harmonic-oscillator (RRHO) approximation. ^d^ Gibbs free energy computed at 298.15 K within the RRHO approximation. ^e^ Electronic energy including nonelectrostatic terms according to the SMD solvation model. ^f^ *G*_SMD,RRHO_ = *G*_PCM,RRHO_ + *G*_SMD_ − *G*_PCM_.

**Table 7:** KP-2e, neutral form in vacuo.

Tautomer	Conformer ^a^	*E* (Ha) ^b^	*H*_RRHO_ (Ha) ^c^	*G*_RRHO_ (Ha) ^d^
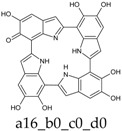	*C*_1_, conf1	−2048.922889 (4.2)	−2048.433678 (4.0)	−2048.531488 (3.1)
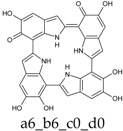	*C*_1_, conf1	−2048.929568 (0.0)	−2048.440034 (0.0)	−2048.536382 (0.0)

In parentheses relative energies (kcal mol^−1^) refer to the most stable form identified at the specified level. ^a^ For chiral structures, only one enantiomer is listed. ^b^ Electronic energy. ^c^ Enthalpy computed at 298.15 K within the rigid-rotor/harmonic-oscillator (RRHO) approximation. ^d^ Gibbs free energy computed at 298.15 K within the RRHO approximation.

**Table 8:** KP-2e, neutral form in water.

Tautomer	Conformer ^a^	*G*_PCM_ (Ha) ^b^	*H*_PCM,RRHO_ (Ha) ^c^	*G*_PCM,RRHO_ (Ha) ^d^	*G*_SMD_ (Ha) ^e^	*G*_SMD,RRHO_ (Ha) ^f^
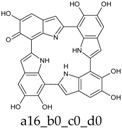	*C*_1_, conf1	−2048.952050 (5.1)	-	-	-	-
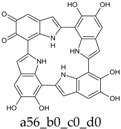	*C*_1_, conf1	−2048.947596 (7.9)	-	-	-	-
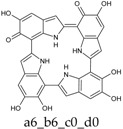	*C*_1_, conf1	−2048.960200 (0.0)	−2048.472201 (0.0)	−2048.569202 (0.0)	−2048.984556 (0.0)	−2048.593558 (0.0)

In parentheses relative energies (kcal mol^−1^) refer to the most stable form identified at the specified level. ^a^ For chiral structures, only one enantiomer is listed. ^b^ Electronic energy including electrostatic contributions at the polarizable continuum model (PCM) level. ^c^ Enthalpy computed at 298.15 K within the rigid-rotor/harmonic-oscillator (RRHO) approximation. ^d^ Gibbs free energy computed at 298.15 K within the RRHO approximation. ^e^ Electronic energy including nonelectrostatic terms according to the SMD solvation model. ^f^ *G*_SMD,RRHO_ = *G*_PCM,RRHO_ + *G*_SMD_ − *G*_PCM_.

**Table 9:** KP-2e, monoanionic form in water.

Tautomer	Conformer ^a^	*G*_PCM_ (Ha) ^b^	*H*_PCM,RRHO_ (Ha) ^c^	*G*_PCM,RRHO_ (Ha) ^d^	*G*_SMD_ (Ha) ^e^	*G*_SMD,RRHO_ (Ha) ^f^
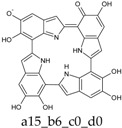	*C*_1_, conf1	−2048.487906 (8.3)	-	-	-	-
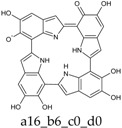	*C*_1_, conf1	−2048.501057 (0.0)	−2048.026967 (0.0)	−2048.123602 (0.0)	−2048.520660 (0.0)	−2048.143205 (0.5)
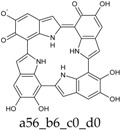	*C*_1_, conf1	−2048.494742 (4.0)	−2048.020572 (4.0)	−2048.118108 (3.4)	−2048.520680 (0.0)	−2048.144046 (0.0)
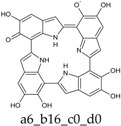	*C*_1_, conf1	−2048.497643 (2.1)	−2048.023707 (2.0)	−2048.120613 (1.9)	−2048.517642 (1.9)	−2048.140612 (2.2)
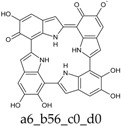	*C*_1_, conf1	−2048.487298 (8.6)	-	-	-	-

In parentheses relative energies (kcal mol^−1^) refer to the most stable form identified at the specified level. ^a^ For chiral structures, only one enantiomer is listed. ^b^ Electronic energy including electrostatic contributions at the polarizable continuum model (PCM) level. ^c^ Enthalpy computed at 298.15 K within the rigid-rotor/harmonic-oscillator (RRHO) approximation. ^d^ Gibbs free energy computed at 298.15 K within the RRHO approximation. ^e^ Electronic energy including nonelectrostatic terms according to the SMD solvation model. ^f^ *G*_SMD,RRHO_ = *G*_PCM,RRHO_ + *G*_SMD_ − *G*_PCM_.

**Table 10:** KP-4e, neutral form in vacuo.

Tautomer	Conformer ^a^	*E* (Ha) ^b^	*H*_RRHO_ (Ha) ^c^	*G*_RRHO_ (Ha) ^d^
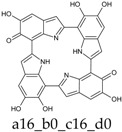	*C*_1_, conf1	−2047.692169 (6.3)		
	*C*_2_, conf1	−2047.692054 (6.3)	-	-
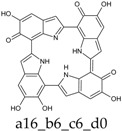	*C*_1_, conf1	−2047.700139 (1.3)	−2047.235705 (1.2)	−2047.331297 (1.1)
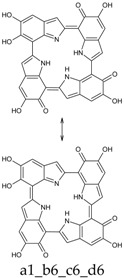	*C*_1_, conf1	−2047.691396 (6.8)	-	-
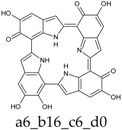	*C*_1_, conf1	−2047.702153 (0.0)	−2047.237674 (0.0)	−2047.333050 (0.0)
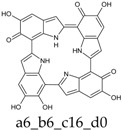	*C*_1_, conf1	−2047.699471 (1.7)	−2047.234863 (1.8)	−2047.330241 (1.8)
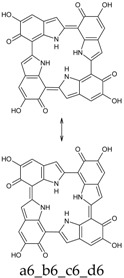	*C*_1_, conf1	−2047.696161 (3.8)	−2047.231453 (3.9)	−2047.325734 (4.6)
	*C*_2_, conf1	−2047.696144 (3.8)	−2047.231368 (4.0)	−2047.324833 (5.2)

In parentheses relative energies (kcal mol^−1^) refer to the most stable form identified at the specified level. ^a^ For chiral structures, only one enantiomer is listed. ^b^ Electronic energy. ^c^ Enthalpy computed at 298.15 K within the rigid-rotor/harmonic-oscillator (RRHO) approximation. ^d^ Gibbs free energy computed at 298.15 K within the RRHO approximation.

**Table 11:** KP-4e, neutral form in water.

Tautomer	Conformer ^a^	*G*_PCM_ (Ha) ^b^	*H*_PCM,RRHO_ (Ha) ^c^	*G*_PCM,RRHO_ (Ha) ^d^	*G*_SMD_ (Ha) ^e^	*G*_SMD,RRHO_ (Ha) ^f^
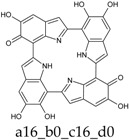	*C*_1_, conf1	−2047.716730 (5.1)	-	-	-	-
	*C*_2_, conf1	−2047.716589 (5.2)	-	-	-	-
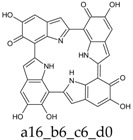	*C*_1_, conf1	−2047.722869 (1.3)	−2047.260061 (1.3)	−2047.355968 (1.4)	−2047.741845 (1.6)	−2047.374944 (1.5)
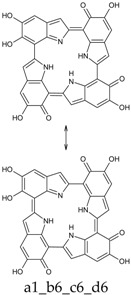	*C*_1_, conf1	−2047.714953 (6.2)	-	-	-	-
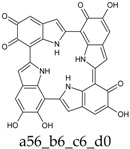	*C*_1_, conf1	−2047.714698 (6.4)	-	-	-	-
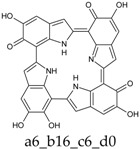	*C*_1_, conf1	−2047.724901 (0.0)	−2047.262199 (0.0)	−2047.358235 (0.0)	−2047.743929 (0.2)	−2047.377263 (0.0)
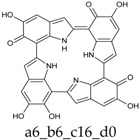	*C*_1_, conf1	−2047.722818 (1.3)	−2047.259858 (1.5)	−2047.355768 (1.5)	−2047.742349 (1.2)	−2047.375299 (1.2)
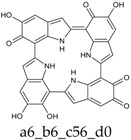	*C*_1_, conf1	−2047.715313 (6.0)	-	-	-	-
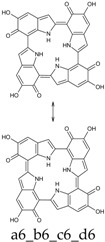	*C*_1_, conf1	−2047.723719 (0.7)	−2047.260186 (1.3)	−2047.354624 (2.3)	−2047.744321 (0.0)	−2047.375226 (1.3)
	*C*_2_, conf1	−2047.723546 (0.9)	−2047.260328 (1.2)	−2047.354611 (2.3)	−2047.743800 (0.3)	−2047.374865 (1.5)

In parentheses relative energies (kcal mol^−1^) refer to the most stable form identified at the specified level. ^a^ For chiral structures, only one enantiomer is listed. ^b^ Electronic energy including electrostatic contributions at the polarizable continuum model (PCM) level. ^c^ Enthalpy computed at 298.15 K within the rigid-rotor/harmonic-oscillator (RRHO) approximation. ^d^ Gibbs free energy computed at 298.15 K within the RRHO approximation. ^e^ Electronic energy including nonelectrostatic terms according to the SMD solvation model. ^f^ *G*_SMD,RRHO_ = *G*_PCM,RRHO_ + *G*_SMD_ − *G*_PCM_.

**Table 12:** KP-6e, neutral form in vacuo.

Tautomer	Conformer ^a^	*E* (Ha) ^b^	*H*_RRHO_ (Ha) ^c^	*G*_RRHO_ (Ha) ^d^
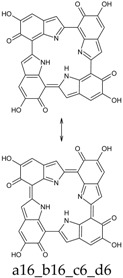	*C*_1_, conf1	−2046.467842 (9.1)	-	-
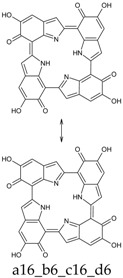	*C*_1_, conf1	−2046.482284 (0.0)	-	-
	*C*_2_, conf1	−2046.482242 (0.0)	−2046.043017 (0.0)	−2046.138676 (0.0)

In parentheses relative energies (kcal mol^−1^) refer to the most stable form identified at the specified level. ^a^ For chiral structures, only one enantiomer is listed. ^b^ Electronic energy. ^c^ Enthalpy computed at 298.15 K within the rigid-rotor/harmonic-oscillator (RRHO) approximation. ^d^ Gibbs free energy computed at 298.15 K within the RRHO approximation.

**Table 13:** KP-6e, neutral form in water.

Tautomer	Conformer ^a^	*G*_PCM_ (Ha) ^b^	*H*_PCM,RRHO_ (Ha) ^c^	*G*_PCM,RRHO_ (Ha) ^d^	*G*_SMD_ (Ha) ^e^	*G*_SMD,RRHO_ (Ha) ^f^
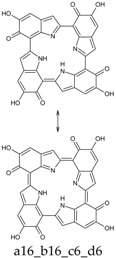	*C*_1_, conf1	−2046.484846 (7.9)	-	-	-	-
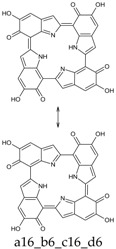	*C*_1_, conf1	−2046.497644 (−0.1)	-	-	-	-
	*C*_2_, conf1	−2046.497479 (0.0)	−2046.059837 (0.0)	−2046.154721 (0.0)	−2046.509330 (0.0)	−2046.166572 (0.0)

In parentheses relative energies (kcal mol^−1^) refer to the most stable form identified at the specified level. ^a^ For chiral structures, only one enantiomer is listed. ^b^ Electronic energy including electrostatic contributions at the polarizable continuum model (PCM) level. ^c^ Enthalpy computed at 298.15 K within the rigid-rotor/harmonic-oscillator (RRHO) approximation. ^d^ Gibbs free energy computed at 298.15 K within the RRHO approximation. ^e^ Electronic energy including nonelectrostatic terms according to the SMD solvation model. ^f^ *G*_SMD,RRHO_ = *G*_PCM,RRHO_ + *G*_SMD_ − *G*_PCM_.

**Table 14:** Excitations underlying the UV–Vis spectrum of a16_b6_c16_d6, computed at the *C*_2h_ geometry in vacuo.

Symmetry	Main CI Contributions (Coefficients) ^a^	λ (nm) (*f*) ^b^
^1^B_u_	HOMO−1 → LUMO (−0.12); HOMO → LUMO (0.60); HOMO → LUMO+1 (−0.32)	1080.6 (0.03)
^1^B_u_	HOMO−4 → LUMO+1 (0.14); HOMO−1 → LUMO (0.16); HOMO−1 → LUMO+1 (−0.15); HOMO → LUMO (0.32); HOMO → LUMO+1 (0.57)	988.5 (0.06)
^1^A_g_	HOMO−3 → LUMO (0.12); HOMO−2 → LUMO (−0.26); HOMO−2 → LUMO+1 (0.41); HOMO−1 → LUMO+2 (−0.11); HOMO → LUMO+2 (0.49)	779.9 (0.00)
^1^B_u_	HOMO−2 → LUMO+2 (−0.11); HOMO−1 → LUMO (0.34); HOMO−1 → LUMO+1 (0.59)	729.8 (0.02)
^1^A_g_	HOMO−2 → LUMO (0.64); HOMO−2 → LUMO+1 (0.22); HOMO → LUMO+2 (0.12)	714.7 (0.00)
^1^A_g_	HOMO−3 → LUMO (−0.35); HOMO−2 → LUMO+1 (0.46); HOMO−1 → LUMO+2 (−0.14); HOMO → LUMO+2 (−0.35)	679.8 (0.00)
^1^B_u_	HOMO−4 → LUMO (0.30); HOMO−1 → LUMO (0.51); HOMO−1 → LUMO+1 (−0.31); HOMO → LUMO+1 (−0.20)	678.2 (0.09)
^1^A_g_	HOMO−3 → LUMO (0.57); HOMO−2 → LUMO+1 (0.20); HOMO → LUMO+2 (−0.34)	638.0 (0.00)
^1^A_g_	HOMO−3 → LUMO (−0.12); HOMO−3 → LUMO+1 (0.67); HOMO−1 → LUMO+2 (0.14)	579.1 (0.00)
^1^B_u_	HOMO−5 → LUMO (0.20); HOMO−5 → LUMO+1 (0.17); HOMO−4 → LUMO (0.54); HOMO−3 → LUMO+2 (0.12); HOMO−2 → LUMO+2 (0.24); HOMO−1 → LUMO (−0.21)	568.8 (0.05)
^1^B_u_	HOMO−5 → LUMO (−0.36); HOMO−5 → LUMO+1 (0.12); HOMO−4 → LUMO+1 (0.49); HOMO−2 → LUMO+2 (0.29); HOMO−1 → LUMO+1 (0.10)	519.9 (0.04)
^1^A_g_	HOMO−3 → LUMO+1 (−0.16); HOMO−2 → LUMO+1 (0.15); HOMO−1 → LUMO+2 (0.66)	506.4 (0.00)
^1^B_u_	HOMO−5 → LUMO+1 (−0.36); HOMO−4 → LUMO (−0.15); HOMO−4 → LUMO+1 (−0.19); HOMO−2 → LUMO+2 (0.54)	487.2 (0.10)
^1^B_u_	HOMO−5 → LUMO (−0.42); HOMO−4 → LUMO+1 (−0.20); HOMO−3 → LUMO+2 (0.51); HOMO−1 → LUMO (−0.10)	458.4 (0.06)
^1^B_u_	HOMO−5 → LUMO (0.30); HOMO−5 → LUMO+1 (0.35); HOMO−4 → LUMO (−0.27); HOMO−4 → LUMO+1 (0.12); HOMO−3 → LUMO+2 (0.36); HOMO−2 → LUMO+2 (0.11); HOMO−1 → LUMO (0.15); HOMO → LUMO+1 (−0.13); HOMO → LUMO+4 (0.13)	430.7 (0.56)
^1^A_g_	HOMO−5 → LUMO+2 (0.12); HOMO−4 → LUMO+2 (0.68); HOMO−3 → LUMO (−0.11)	418.5 (0.00)
^1^B_u_	HOMO−5 → LUMO (−0.19); HOMO−5 → LUMO+1 (0.43); HOMO−4 → LUMO+1 (−0.35); HOMO−3 → LUMO+2 (−0.26); HOMO−2 → LUMO+2 (0.15); HOMO → LUMO (0.16); HOMO → LUMO+4 (0.11); HOMO → LUMO+5 (−0.12)	418.0 (0.79)
^1^B_g_	HOMO-10 → LUMO (0.24); HOMO-10 → LUMO+1 (0.20); HOMO-9 → LUMO+2 (−0.25); HOMO-8 → LUMO+2 (−0.10); HOMO-6 → LUMO (0.54); HOMO-6 → LUMO+1 (0.15)	407.1 (0.00)
^1^A_u_	HOMO-10 → LUMO+2 (−0.12); HOMO-9 → LUMO (0.49); HOMO-9 → LUMO+1 (0.14); HOMO-8 → LUMO (0.23); HOMO-8 → LUMO+1 (0.27); HOMO-6 → LUMO+2 (−0.28)	404.4 (0.00)

^a^ HOMO: Highest energy occupied molecular orbital; LUMO: Lowest energy unoccupied molecular orbital. ^b^ Only transitions with λ > 400 nm are listed.

**Table 15:** KP-8e, neutral form in vacuo.

Tautomer	Conformer ^a^	*E* (Ha) ^b^	*H*_RRHO_ (Ha) ^c^	*G*_RRHO_ (Ha) ^d^
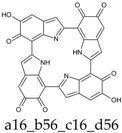	*C*_1_, conf1	−2045.192843 (−0.1)	-	-
	*C*_1_, conf2	−2045.192633 (0.0)	-	-
	*C*_2_, conf1	−2045.192633 (0.0)	−2044.778733 (0.0)	−2044.873783 (0.0)

In parentheses relative energies (kcal mol^−1^) refer to the most stable form identified at the specified level. ^a^ For chiral structures, only one enantiomer is listed. ^b^ Electronic energy. ^c^ Enthalpy computed at 298.15 K within the rigid-rotor/harmonic-oscillator (RRHO) approximation. ^d^ Gibbs free energy computed at 298.15 K within the RRHO approximation.

**Table 16:** KP-8e, neutral form in water.

Tautomer	Conformer ^a^	*G*_PCM_ (Ha) ^b^	*H*_PCM,RRHO_ (Ha) ^c^	*G*_PCM,RRHO_ (Ha) ^d^	*G*_SMD_ (Ha) ^e^	*G*_SMD,RRHO_ (Ha) ^f^
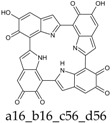	*C*_1_, conf1	−2045.205247 (9.5)	-	-	-	-
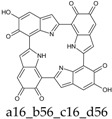	*C*_1_, conf1	−2045.220522 (0.0)	-	-	-	-
	*C*_2_, conf1	−2045.220445 (0.0)	−2044.807661 (0.0)	−2044.902116 (0.0)	−2045.234643 (0.0)	−2044.916314 (0.0)
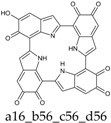	*C*_1_, conf1	−2045.208959 (7.2)	-	-	-	-

In parentheses relative energies (kcal mol^−1^) refer to the most stable form identified at the specified level. ^a^ For chiral structures, only one enantiomer is listed. ^b^ Electronic energy including electrostatic contributions at the polarizable continuum model (PCM) level. ^c^ Enthalpy computed at 298.15 K within the rigid-rotor/harmonic-oscillator (RRHO) approximation. ^d^ Gibbs free energy computed at 298.15 K within the RRHO approximation. ^e^ Electronic energy including nonelectrostatic terms according to the SMD solvation model. ^f^ *G*_SMD,RRHO_ = *G*_PCM,RRHO_ + *G*_SMD_ − *G*_PCM_.

**Table 17:** HOMO and LUMO energies, and corresponding HOMO–LUMO gaps, computed at the PBE0/6-311++G(2d,2p)//BE0/6-31+G(d,p) level in vacuo and in water.

Oxidation State	Tautomer	Conformer	*E*_HOMO_ (eV)	*E*_LUMO_ (eV)	Gap (eV)	*E*_HOMO_ (eV)	*E*_LUMO_ (eV)	Gap (eV)
			In Vacuo			In Water		
KP-Red	a0_b0_c0_d0	*S*_4_	−5.074	−1.308	3.767	−5.363	−1.499	3.864
KP-1e	a5_b0_c0_d0	*C*_1_, conf1	−5.341	−1.556	3.784	−5.465	−1.644	3.822
	a6_b0_c0_d0	*C*_1_, conf1	−5.073	−1.512	3.561	−5.314	−1.609	3.705
KP-2e	a16_b0_c0_d0	*C*_1_, conf1	−5.269	−3.449	1.821	−5.480	−3.696	1.783
	a6_b6_c0_d0	*C*_1_, conf1	−5.272	−3.708	1.565	−5.329	−3.834	1.495
KP-4e	a16_b0_c16_d0	*C*_2_, conf1	-	-	-	−5.603	−3.748	1.855
	a16_b6_c6_d0	*C*_1_, conf1	−5.506	−4.114	1.392	−5.443	−4.086	1.357
	a6_b16_c6_d0	*C*_1_, conf1	−5.479	−4.084	1.395	−5.443	−4.057	1.386
	a6_b6_c16_d0	*C*_1_, conf1	−5.451	−4.053	1.398	−5.418	−4.020	1.398
	a6_b6_c6_d6	*C*_2_, conf1	−5.479	−4.371	1.108	−5.280	−4.145	1.135
KP-6e	a16_b6_c16_d6	*C*_2_, conf1	−5.978	−4.280	1.698	−5.890	−4.171	1.719
KP-8e	a16_b56_c16_d56	*C*_2_, conf1	−6.393	−4.787	1.605	−6.132	−4.558	1.574

**Table 18:** Reaction free energies computed for several disproportionation processes involving KP.

Reaction	∆*G*_RRHO_ (kcal mol^−1^) (In Vacuo) ^a^	∆*G*_SMD,RRHO_ (kcal mol^−1^) (In Water) ^b^
2 KP-1e ⇌ KP-Red + KP-2e	−0.1 (−0.0_4_)	1.6 (0.8)
2 KP-2e ⇌ KP-Red + KP-4e	−0.1 (−0.0_5_)	0.8 (0.4)
2 KP-4e ⇌ KP-2e + KP-6e	−5.6 (−2.8)	−3.5 (−1.8)
2 KP-6e ⇌ KP-4e + KP-8e	44.3 (22.1)	24.8 (12.4)
6 KP-1e ⇌ 5 KP-Red + KP-6e	−6.3 (−1.0)	2.9 (0.5)
3 KP-2e ⇌ 2 KP-Red + KP-6e	−5.8 (−1.9)	−1.9 (−0.6)
3 KP-4e ⇌ KP-Red + 2 KP-6e	−11.3 (−3.8)	−6.2 (−2.1)
4 KP-6e ⇌ KP-Red + 3 KP-8e	121.4 (30.4)	68.2 (17.1)

For each oxidation state and condition (vacuo, water), the free energy of the most stable form identified was used in the calculations. In parentheses values referred to the conversion of one mole of reactant. ^a^ Reaction free energy computed at 298.15 K within the rigid-rotor/harmonic-oscillator (RRHO) approximation. ^b^ Reaction free energy computed at 298.15 K at the polarizable continuum model (PCM) level within the RRHO approximation, and including nonelectrostatic terms according to the SMD solvation model (*G*_SMD,RRHO_ = *G*_PCM,RRHO_ + *G*_SMD_ − *G*_PCM_).

## Supplementary Materials

The following are available online at www.mdpi.com/2313-7673/2/4/21/s1. Figure S1: Selected molecular orbitals of the a16_b6_c16_d6 tautomer of KP-6e, computed in vacuo at the *C*_2h_ geometry, Figure S2: Ultraviolet–visible spectrum of the parent non-oxygenated porphyrin ([31], compound **1**) and of a related brominated and oxidized aromatic derivative (*ibid*., compound **8**) computed at different theory levels, Figure S3: Ultraviolet–visible spectra of the most significant tautomers/conformers (neutral forms) in the different oxidation states, computed at the TD-B3LYP/6-311++G(2d,2p)//PBE0/6-31+G(d,p) level in vacuo, Figure S4: Molecular structures of the most significant tautomers/conformers (neutral forms in water) in the different oxidation states, Tables S1–S15: Structures, energies and relative stabilities of the most stable tautomers/conformers in all oxidation/protonation states explored, in vacuo and in water, Tables S16 and S17: Excitations underlying the UV–Vis spectrum of the a16_b6_c16_d6 tautomer of KP-6e, computed in vacuo at the *C*_2h_ geometry and at the *C*_2_ geometry, Table S18: Comparison of the electronic energies of the main tautomers/conformers of the neutral form of KP-4e, computed in vacuo at different theory levels, Tables S19 and S20: Electronic energies of main neutral species of KP computed in vacuo at different theory levels, and resulting electronic energy changes for disproportionation processes, Tables S21 and S22: Comparison of the electronic transitions of porphin freebase and of magnesium-porphin, computed in vacuo at different levels.
