# Supplementary material for: Kaxiras’s Porphyrin: DFT Modeling of Redox-Tuned Optical and Electronic Properties in a Theoretically Designed Catechol-Based Bioinspired Platform
Source: Biomimetics (Basel). 2017 Nov 7;2(4):21. doi: 10.3390/biomimetics2040021 (PMC6352670; doi:10.3390/biomimetics2040021)
Supplement: Supplementary file 1 [file biomimetics-02-00021-s001.pdf]

Biomimetics **2017**, *2*, 21

Supplementary Materials: Kaxiras's Porphyrin: DFT Modeling of Redox-Tuned Optical and Electronic Properties in a Theoretically Designed Catechol-Based Bioinspired Platform

Orlando Crescenzi *, Marco d'Ischia and Alessandra Napolitano

Department of Chemical Sciences, University of Naples Federico II, I-80126 Naples, Italy; dischia@unina.it (M.d.I.); alesnapo@unina.it (A.N.)
* Correspondence: orlando.crescenzi@unina.it; Tel.: +39-081-674206

**a16_b6_c16_d6 (C_{2h})**	**Magnesium–porphyrin (D_{4h})**	**Porphyrin free base (D_{2h})**

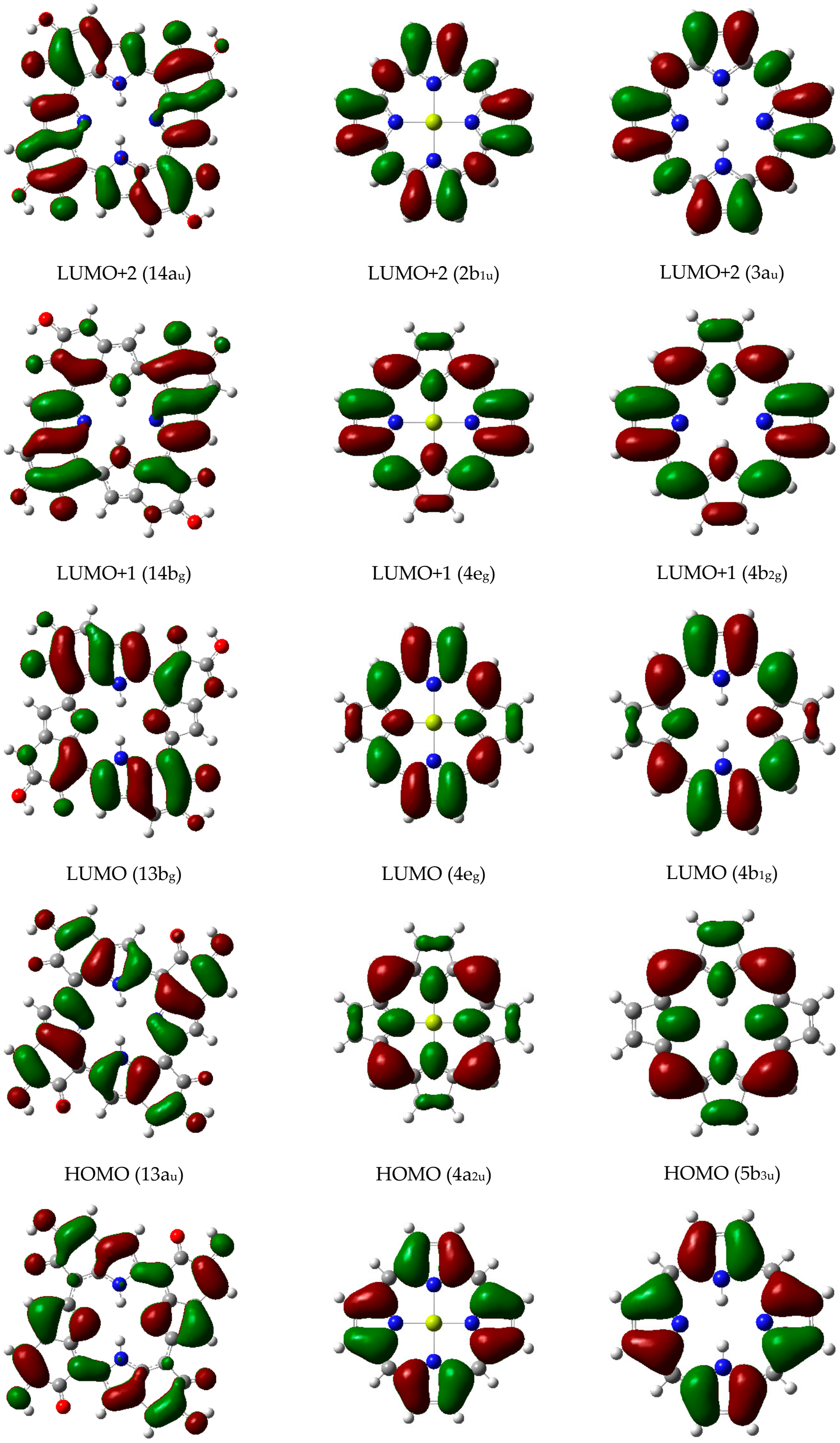

a16_b6_c16_d6 (C_{2h})	Magnesium–porphyrin (D_{4h})	Porphyrin free base (D_{2h})
LUMO+2 ($14a_u$)	LUMO+2 ($2b_{1u}$)	LUMO+2 ($3a_u$)
LUMO+1 ($14b_g$)	LUMO+1 ($4e_g$)	LUMO+1 ($4b_{2g}$)
LUMO ($13b_g$)	LUMO ($4e_g$)	LUMO ($4b_{1g}$)
HOMO ($13a_u$)	HOMO ($4a_{2u}$)	HOMO ($5b_{3u}$)
HOMO−1 ($12a_u$)	HOMO−1 ($1a_{1u}$)	HOMO−1 ($2a_u$)

Figure S1. *Cont.*

a16_b6_c16_d6 (C2h)	Magnesium–porphyrin (D4h)	Porphyrin free base (D2h)

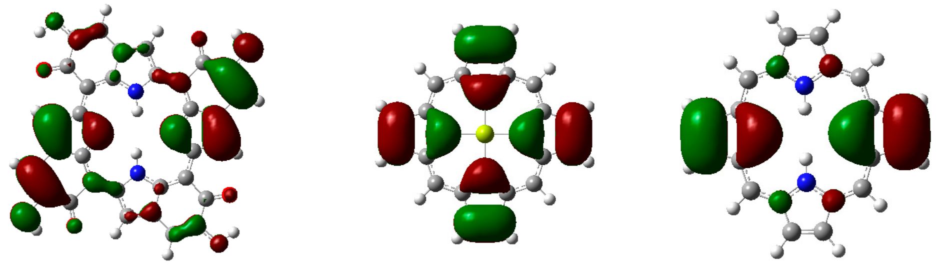

HOMO−2 ($12b_g$) | HOMO−2 ($2b_{2u}$) | HOMO−2 ($3b_{1g}$)

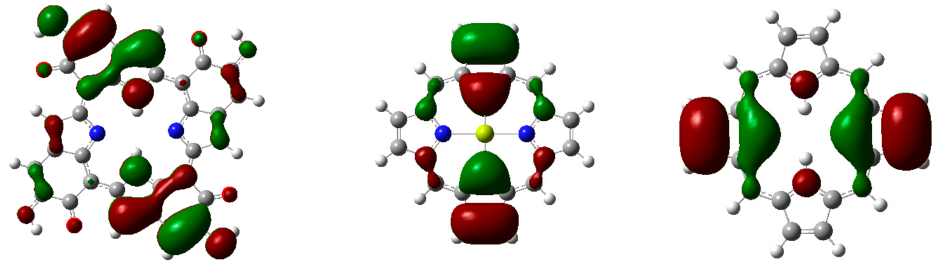

HOMO−3 ($11b_g$) | HOMO−3 ($3e_g$) | HOMO−3 ($4b_{3u}$)

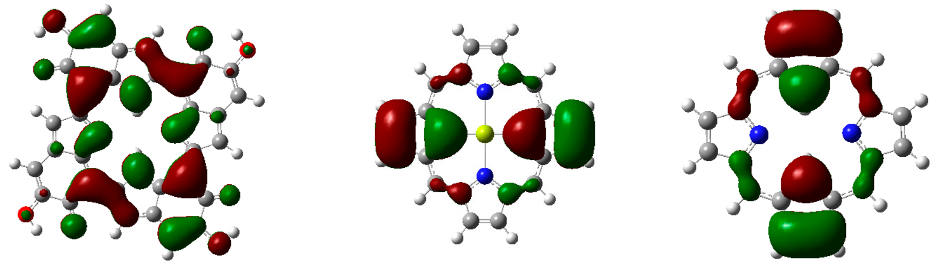

HOMO−4 ($11a_u$) | HOMO−4 ($3e_g$) | HOMO−4 ($3b_{2g}$)

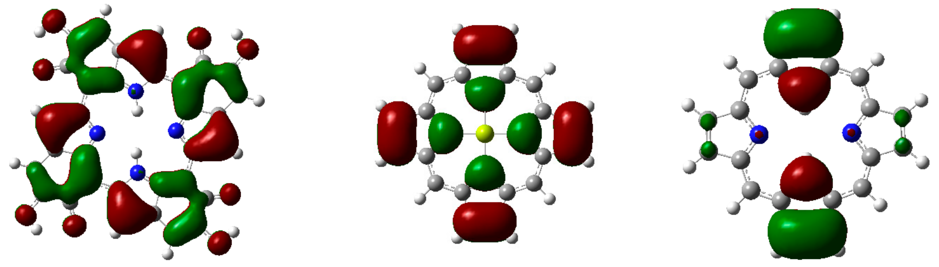

HOMO−5 ($10a_u$) | HOMO−5 ($3a_{2u}$) | HOMO−5 ($3b_{3u}$)

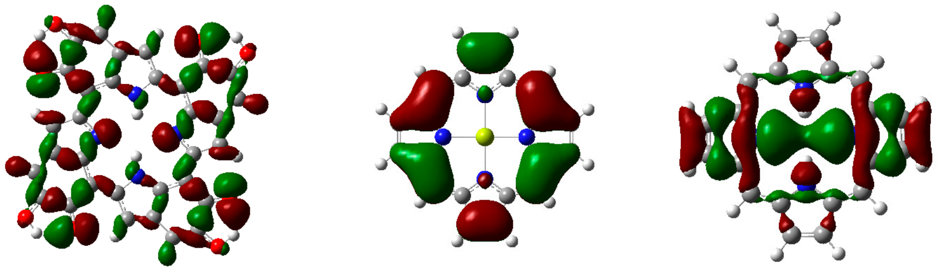

HOMO−6 ($62a_g$) | HOMO−6 ($2e_g$) | HOMO−6 ($20a_g$)

Figure S1. Selected molecular orbitals of the a16_b6_c16_d6 tautomer of KP-6e, computed in vacuo at the C_{2h} geometry. For comparison, corresponding molecular orbitals obtained at the same level for magnesium porphyrin and for porphyrin free base are also shown.

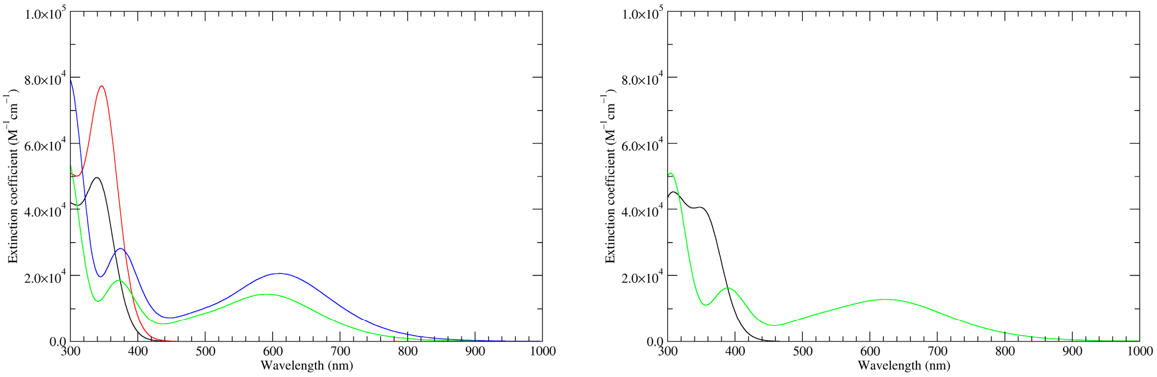

(**a**) (**b**)

Figure S2. Computed UV−Vis spectrum of the parent non−oxygenated porphyrin ([1], compound **1**) and of a related brominated and oxidized aromatic derivative (*ibid*., compound **8**). All compounds were modeled with methyl groups in lieu of the *tert*-butyl substituents. (**a**) TD-PBE0/6-311++G(2d,2p) // PBE0/6-31+G(d,p) level; black line, **1** in vacuo; red line, **1** in toluene; green line, **8** in vacuo; blue line, **8** in toluene. (**b**) TD-B3LYP/6-311++G(2d,2p) // PBE0/6-31+G(d,p) level; black line, **1** in vacuo; red line, green line, **8** in vacuo.

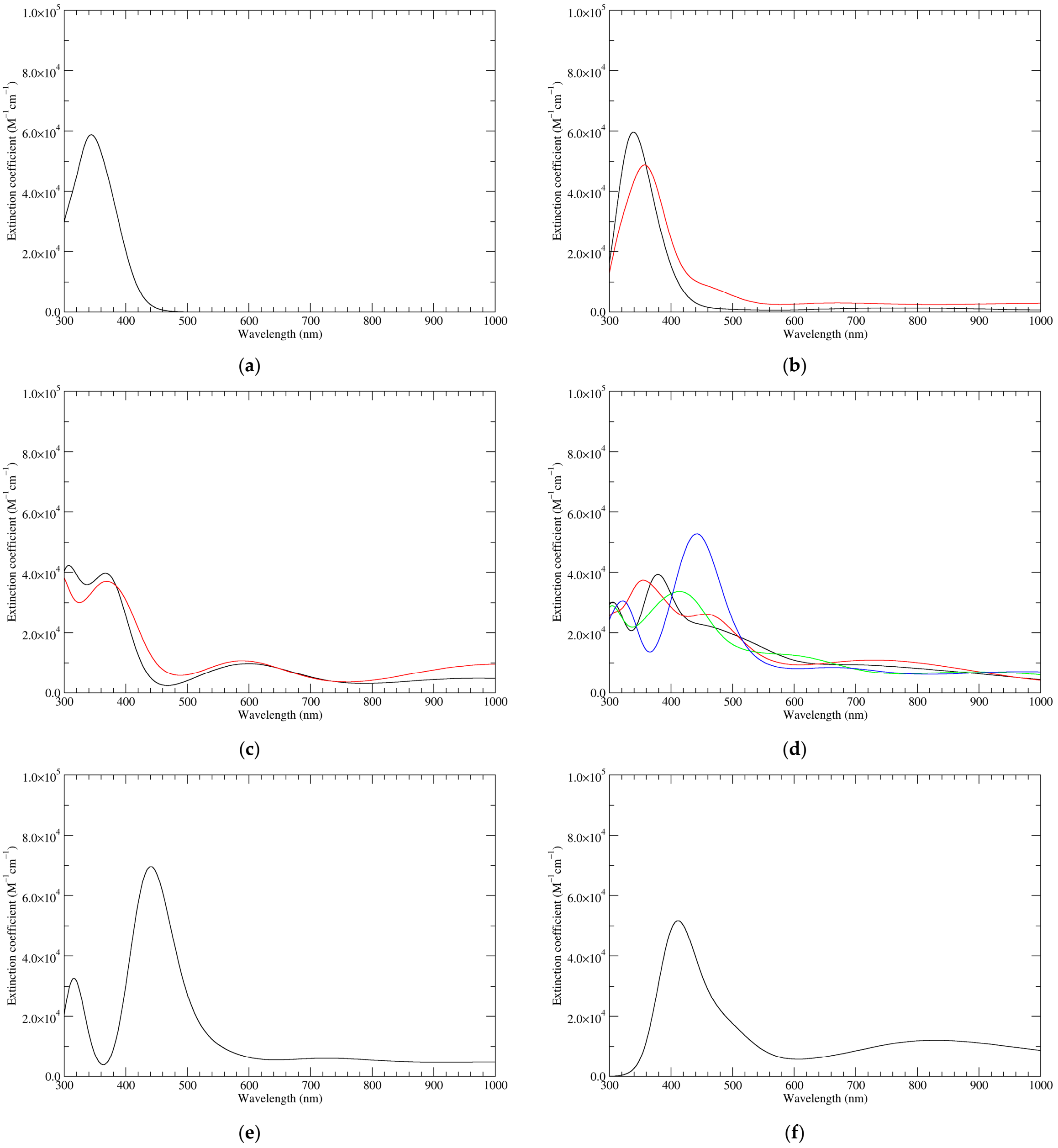

Figure S3. Ultraviolet–visible spectra of the most significant tautomers /conformers (neutral forms) in the different oxidation states, computed at the TD-B3LYP/6-311++G(2d,2p) // PBE0/6-31+G(d,p) level in vacuo. (**a**) Reduced state, a0_b0_c0_d0, S_4. (**b**) One-electron oxidation state: black line, a5_b0_c0_d0, C_1, conf1; red line, a6_b0_c0_d0, C_1, conf1. (**c**) Two-electrons oxidation state: black line, a16_b0_c0_d0, C_1, conf1; red line, a6_b6_c0_d0, C_1, conf1. (**d**) Four-electrons oxidation state: black line, a16_b6_c6_d0, C_1, conf1; red line, a6_b16_c6_d0, C_1, conf1; green line, a6_b6_c16_d0, C_1, conf1; blue line, a6_b6_c6_d6, C_2, conf1. (**e**) Six-electrons oxidation state, a16_b6_c16_d6, C_2, conf1. (**f**) Eight-electrons oxidation state, a16_b56_c16_d56, C_2, conf1.

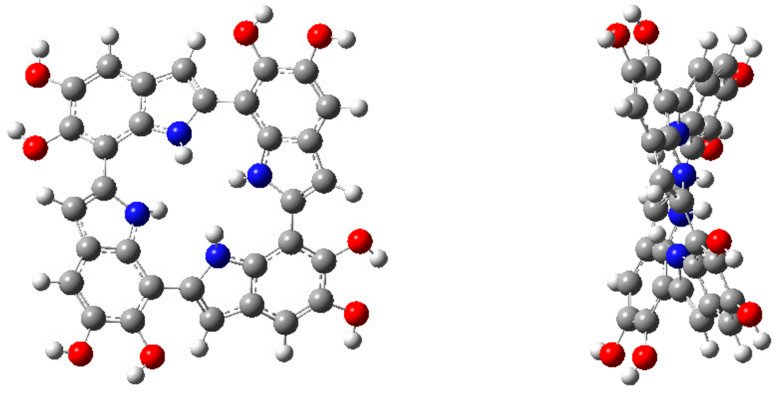

(**a**)

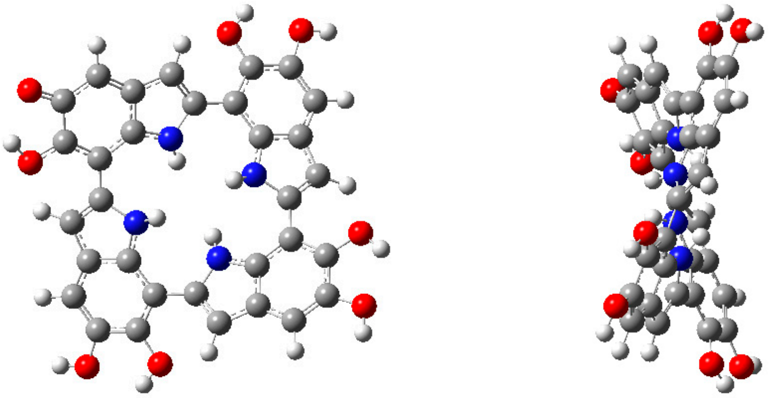

(**b**)

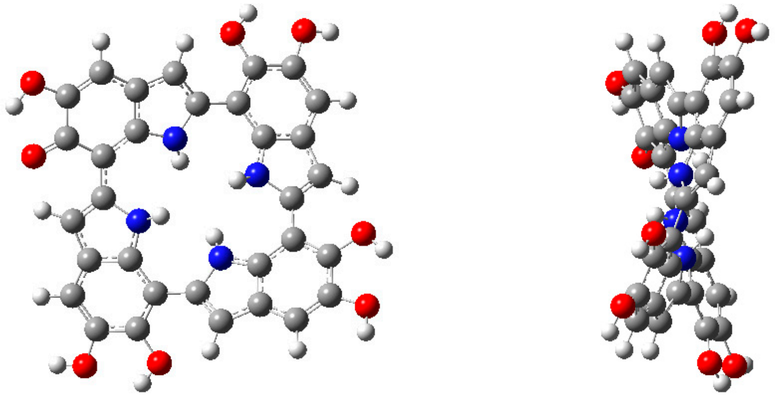

(**c**)

Figure S4. *Cont.*

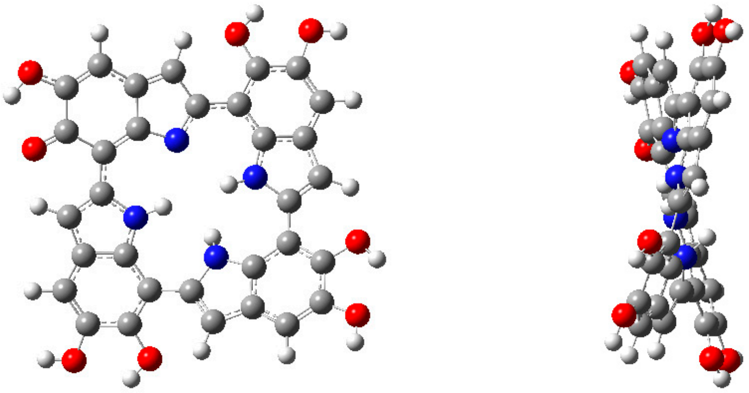

(**d**)

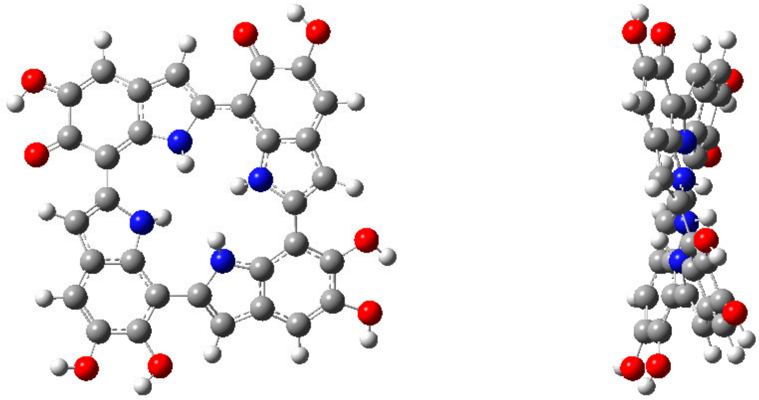

(**e**)

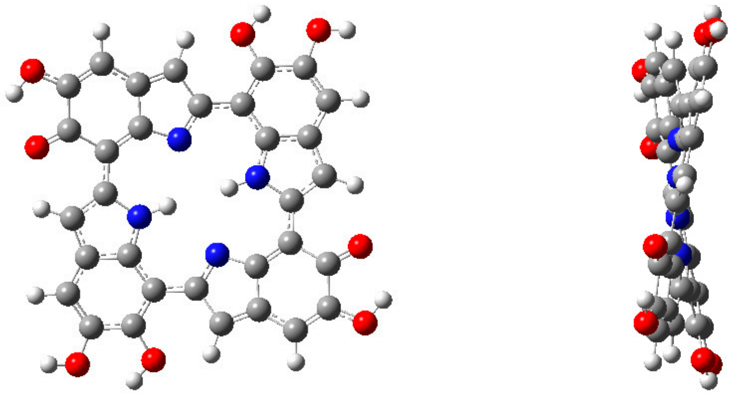

(**f**)

Figure S4. *Cont.*

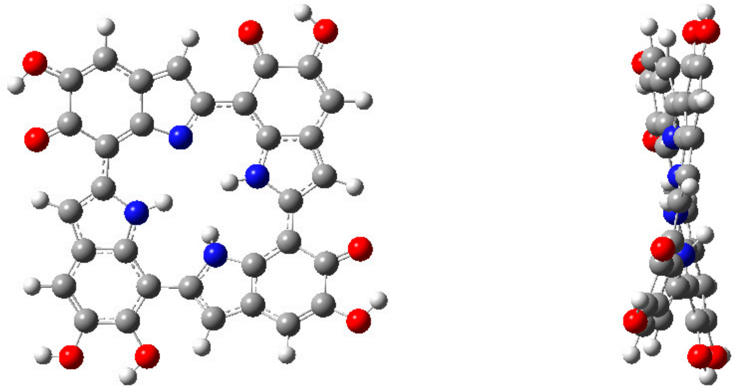

(**g**)

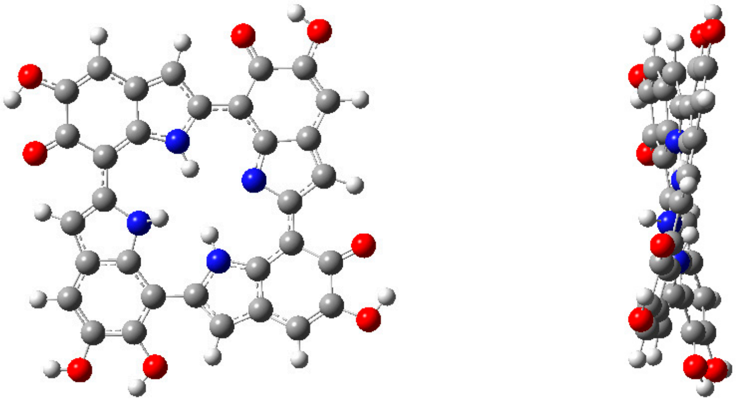

(**h**)

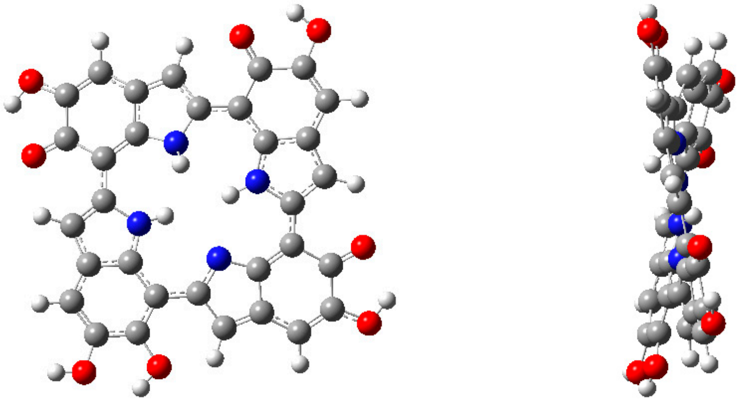

(**i**)

Figure S4. *Cont.*

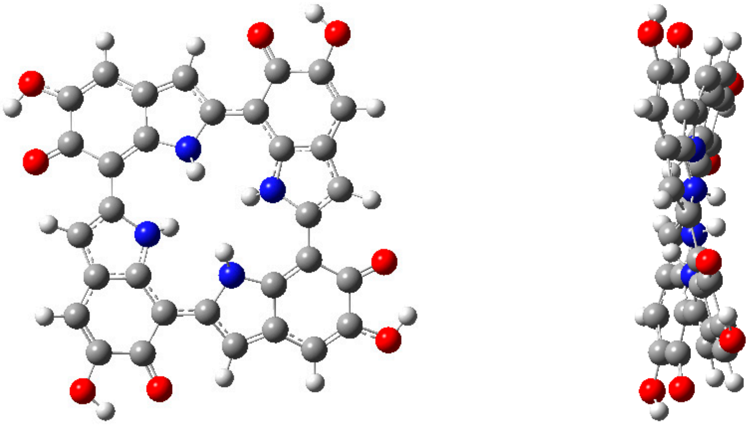

(**j**)

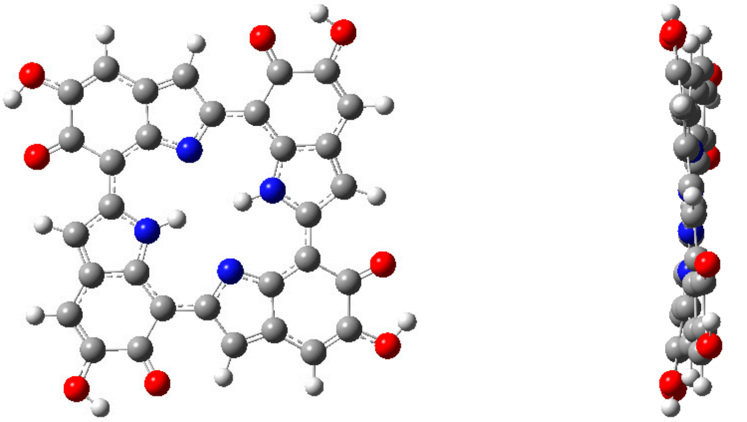

(**k**)

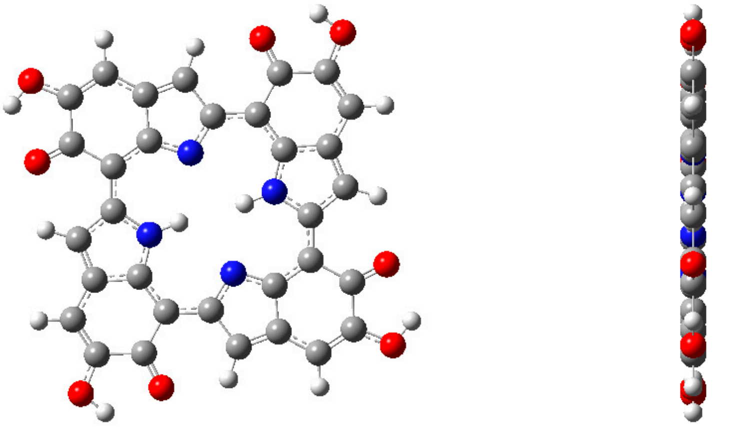

(**l**)

Figure S4. *Cont.*

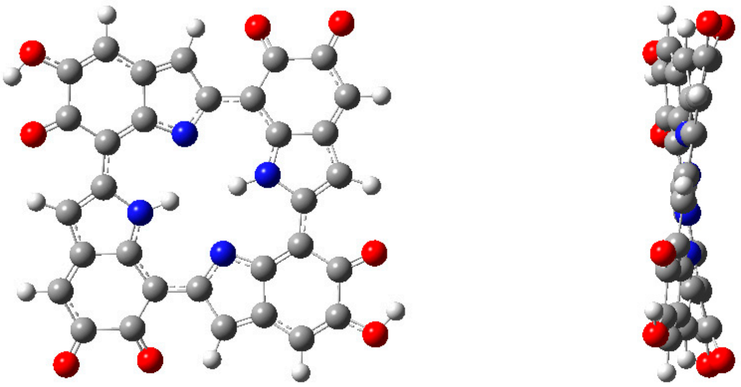

(**m**)

Figure S4. Molecular structures of the most significant tautomers/conformers (neutral forms in water) in the different oxidation states. For each structure, two views are shown, related by a 90-degree rotation along a vertical axis. (**a**) Reduced state, a0_b0_c0_d0, S_4. (**b**) One-electron oxidation state, a5_b0_c0_d0, C_1, conf1. (**c**) One-electron oxidation state, a6_b0_c0_d0, C_1, conf1. (**d**) Two-electrons oxidation state, a16_b0_c0_d0, C_1, conf1. (**e**) Two-electrons oxidation state, a6_b6_c0_d0, C_1, conf1. (**f**) Four-electrons oxidation state, a16_b0_c16_d0, C_2, conf1. (**g**) Four-electrons oxidation state, a16_b6_c6_d0, C_1, conf1. (**h**) Four-electrons oxidation state, a6_b16_c6_d0, C_1, conf1. (**i**) Four-electrons oxidation state, a6_b6_c16_d0, C_1, conf1. (**j**) Four-electrons oxidation state, a6_b6_c6_d6, C_2, conf1. (**k**) Six-electrons oxidation state, a16_b6_c16_d6, C_2, conf1. (**l**) Six-electrons oxidation state, a16_b6_c16_d6, C_{2h}, conf1. (**m**) Eight-electrons oxidation state, a16_b56_c16_d56, C_2, conf1.

Table S1. KP-Red, neutral form in vacuo.

Tautomer	Conformer [a]	E (Ha) [b]	H_{RRHO} (Ha) [c]	G_{RRHO} (Ha) [d]
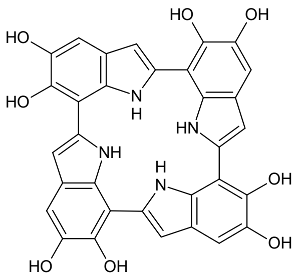 a0_b0_c0_d0	C_1	Evolves to S_4	-	-
	C_2	Evolves to S_4	-	-
	C_4	−2050.111991 (27.1)	Fourth-order saddle point	-
	S_4	**−2050.155247 (0.0)**	**−2049.641430 (0.0)**	**−2049.739870 (0.0)**
	C_{4h}	−2050.111036 (27.7)	Fifth-order saddle point	-

In parentheses relative energies (kcal mol^{-1}) refer to the most stable form (in bold) identified at the specified level. [a] For chiral structures, only one enantiomer is listed. [b] Electronic energy. [c] Enthalpy computed at 298.15 K within the rigid-rotor/harmonic-oscillator (RRHO) approximation. [d] Gibbs free energy computed at 298.15 K within the RRHO approximation.

Table S2. KP-Red, neutral form in water.

Tautomer	Conformer [a]	G_{PCM} (Ha) [b]	$H_{PCM,RRHO}$ (Ha) [c]	$G_{PCM,RRHO}$ (Ha) [d]	G_{SMD} (Ha) [e]	$G_{SMD,RRHO}$ (Ha) [f]
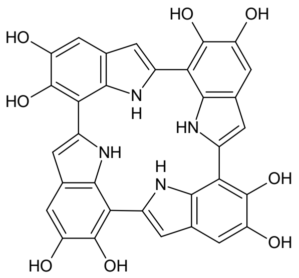 a0_b0_c0_d0	C_1	Evolves to S_4	-	-	-	-
	C_2	Evolves to S_4	-	-	-	-
	C_4	−2050.150972 (25.1)	Fourth-order saddle point	-	-	-
	S_4	**−2050.191047 (0.0)**	**−2049.678884 (0.0)**	**−2049.779144 (0.0)**	**−2050.220499 (0.0)**	**−2049.808596 (0.0)**
	C_{4h}	−2050.148120 (26.9)	Fifth-order saddle point	-	-	-

In parentheses relative energies (kcal mol^{-1}) refer to the most stable form (in bold) identified at the specified level. [a] For chiral structures, only one enantiomer is listed. [b] Electronic energy including electrostatic contributions at the polarizable continuum model (PCM) level. [c] Enthalpy computed at 298.15 K within the rigid-rotor/harmonic-oscillator (RRHO) approximation. [d] Gibbs free energy computed at 298.15 K within the RRHO approximation. [e] Electronic energy including nonelectrostatic terms according to the SMD solvation model. [f] $G_{SMD,RRHO} = G_{PCM,RRHO} + G_{SMD} - G_{PCM}$.

Table S3. KP-Red, monoanionic form in water.

Tautomer	Conformer [a]	G_{PCM} (Ha) [b]	$H_{PCM,RRHO}$ (Ha) [c]	$G_{PCM,RRHO}$ (Ha) [d]	G_{SMD} (Ha) [e]	$G_{SMD,RRHO}$ (Ha) [f]
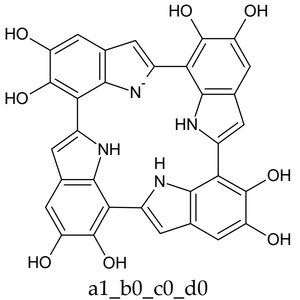 a1_b0_c0_d0	C_1, conf1	−2049.709473 (8.6)	-	-	-	-
	C_1, conf2	Evolves to C_1, conf1	-	-	-	-
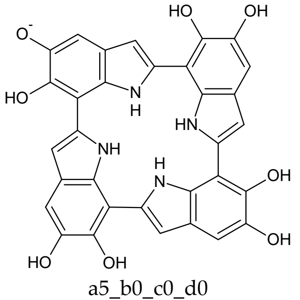 a5_b0_c0_d0	C_1, conf1	−2049.719292 (2.4)	−2049.220534 (2.6)	−2049.319341 (2.4)	−2049.749637 (1.7)	−2049.349686 (1.7)
	C_1, conf2	Evolves to C_1, conf1	-	-	-	-
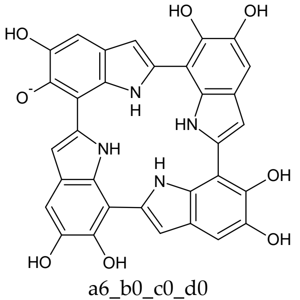 a6_b0_c0_d0	C_1, conf1	**−2049.723100 (0.0)**	**−2049.224673 (0.0)**	**−2049.323215 (0.0)**	**−2049.752271 (0.0)**	**−2049.352386 (0.0)**
	C_1, conf2	Evolves to C_1, conf1	-	-	-	-

In parentheses relative energies (kcal mol^{-1}) refer to the most stable form (in bold) identified at the specified level. [a] For chiral structures, only one enantiomer is listed. [b] Electronic energy including electrostatic contributions at the polarizable continuum model (PCM) level. [c] Enthalpy computed at 298.15 K within the rigid-rotor/harmonic-oscillator (RRHO) approximation. [d] Gibbs free energy computed at 298.15 K within the RRHO approximation. [e] Electronic energy including nonelectrostatic terms according to the SMD solvation model. [f] $G_{SMD,RRHO} = G_{PCM,RRHO} + G_{SMD} - G_{PCM}$.

Table S4. KP-1e, neutral form in vacuo.

Tautomer	Conformer [a]	E (Ha) [b]	H_{RRHO} (Ha) [c]	G_{RRHO} (Ha) [d]
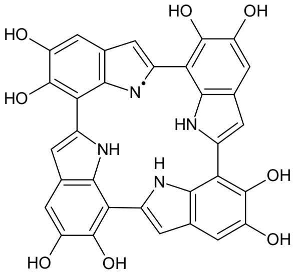 a1_b0_c0_d0	C_1, conf1	−2049.519425 (13.6)	−2049.019231 (13.0)	−2049.118845 (12.1)
	C_1, conf2	Evolves to C_1, conf1	-	-
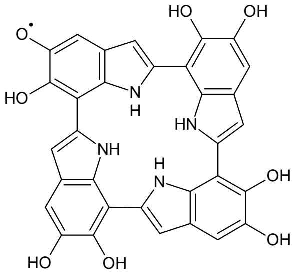 a5_b0_c0_d0	C_1, conf1	−2049.532339 (5.5)	−2049.031222 (5.4)	−2049.129769 (5.2)
	C_1, conf2	Evolves to C_1, conf1	-	-
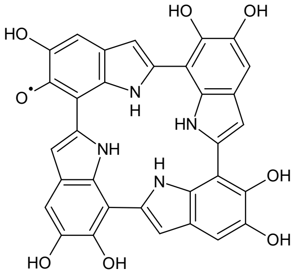 a6_b0_c0_d0	C_1, conf1	**−2049.541050 (0.0)**	**−2049.039878 (0.0)**	**−2049.138067 (0.0)**
	C_1, conf2	Evolves to C_1, conf1	-	-

In parentheses relative energies (kcal mol^{-1}) refer to the most stable form (in bold) identified at the specified level. [a] For chiral structures, only one enantiomer is listed. [b] Electronic energy. [c] Enthalpy computed at 298.15 K within the rigid-rotor/harmonic-oscillator (RRHO) approximation. [d] Gibbs free energy computed at 298.15 K within the RRHO approximation.

Table S5. KP-1e, neutral form in water.

Tautomer	Conformer [a]	G_{PCM} (Ha) [b]	$H_{PCM,RRHO}$ (Ha) [c]	$G_{PCM,RRHO}$ (Ha) [d]	G_{SMD} (Ha) [e]	$G_{SMD,RRHO}$ (Ha) [f]
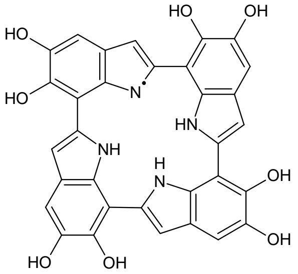 a1_b0_c0_d0	C_1, conf1	−2049.553629 (13.5)	-	-	-	-
	C_1, conf2	Evolves to C_1, conf1	-	-	-	-
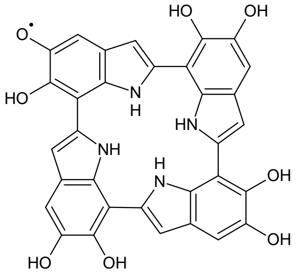 a5_b0_c0_d0	C_1, conf1	−2049.567367 (4.9)	−2049.067779 (4.8)	−2049.167187 (5.1)	−2049.594711 (4.7)	−2049.194531 (4.9)
	C_1, conf2	Evolves to C_1, conf1	-	-	-	-
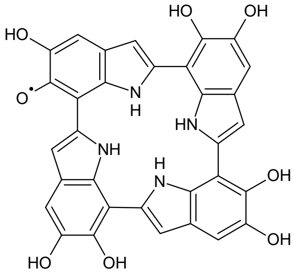 a6_b0_c0_d0	C_1, conf1	**−2049.575106 (0.0)**	**−2049.075489 (0.0)**	**−2049.175302 (0.0)**	**−2049.602176 (0.0)**	**−2049.202372 (0.0)**
	C_1, conf2	Evolves to C_1, conf1	-	-	-	-

In parentheses relative energies (kcal mol^{-1}) refer to the most stable form (in bold) identified at the specified level. [a] For chiral structures, only one enantiomer is listed. [b] Electronic energy including electrostatic contributions at the polarizable continuum model (PCM) level. [c] Enthalpy computed at 298.15 K within the rigid-rotor/harmonic-oscillator (RRHO) approximation. [d] Gibbs free energy computed at 298.15 K within the RRHO approximation. [e] Electronic energy including nonelectrostatic terms according to the SMD solvation model. [f] $G_{SMD,RRHO} = G_{PCM,RRHO} + G_{SMD} - G_{PCM}$.

Table S6. KP-1e, monoanionic form in water.

Tautomer	Conformer [a]	G_{PCM} (Ha) [b]	$H_{PCM,RRHO}$ (Ha) [c]	$G_{PCM,RRHO}$ (Ha) [d]	G_{SMD} (Ha) [e]	$G_{SMD,RRHO}$ (Ha) [f]
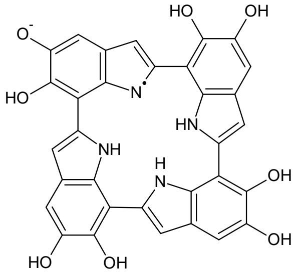 a15_b0_c0_d0	C_1, conf1	−2049.099703 (11.1)	-	-	-	-
	C_1, conf2	Evolves to C_1, conf1	-	-	-	-
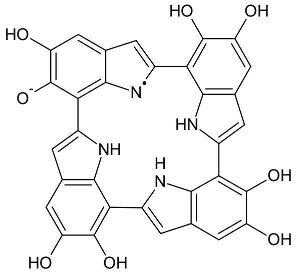 a16_b0_c0_d0	C_1, conf1	−2049.105711 (7.3)	-	-	-	-
	C_1, conf2	Evolves to C_1, conf1	-	-	-	-
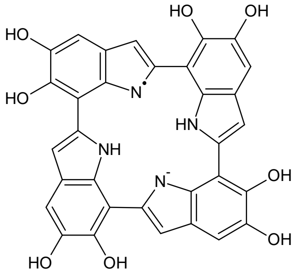 a1_b0_c1_d0	C_1, conf1	−2049.070770 (29.2)	-	-	-	-
	C_1, conf2	Evolves to C_1, conf1	-	-	-	-
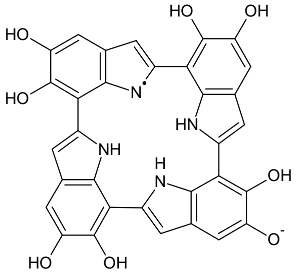 a1_b0_c5_d0	C_1, conf1	−2049.086228 (19.5)	-	-	-	-
	C_1, conf2	−2049.082061 (22.1)	-	-	-	-
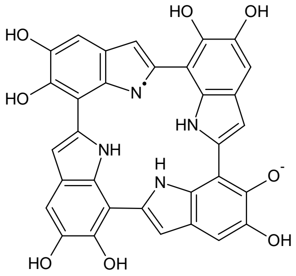 a1_b0_c6_d0	C_1, conf1	−2049.095838 (13.5)	-	-	-	-
	C_1, conf2	−2049.086042 (19.6)	-	-	-	-
	C_1, conf2	Evolves to C_1, conf1				

Table S6. *Cont.*

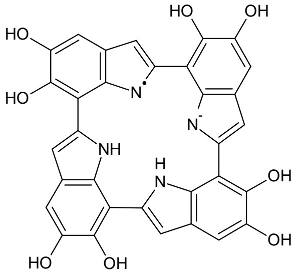 a1_b1_c0_d0	C_1, conf1	−2049.089417 (17.5)	-	-	-	-
	C_1, conf2	Evolves to C_1, conf1	-	-	-	-
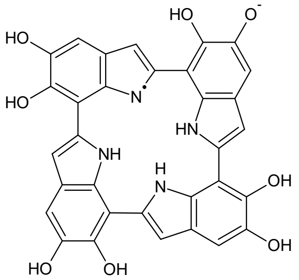 a1_b5_c0_d0	C_1, conf1	−2049.089417 (17.5)	-	-	-	-
	C_1, conf2	Evolves to C_1, conf1	-	-	-	-
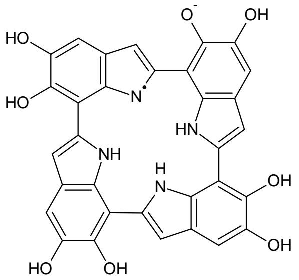 a1_b6_c0_d0	C_1, conf1	−2049.100548 (10.5)	-	-	-	-
	C_1, conf2	−2049.100435 (10.6)	-	-	-	-
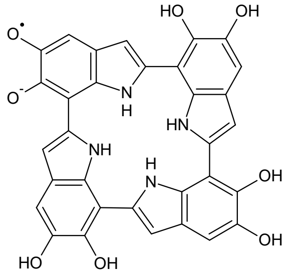 a56_b0_c0_d0	C_1, conf1	−2049.098719 (11.7)	-	-	-	-
	C_1, conf2	Evolves to C_1, conf1	-	-	-	-
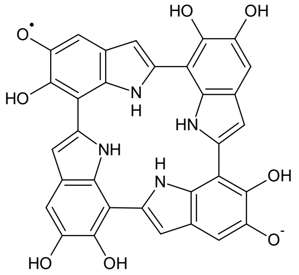 a5_b0_c5_d0	C_1, conf1	−2049.089244 (17.6)	-	-	-	-
	C_1, conf2	Evolves to C_1, conf1	-	-	-	-
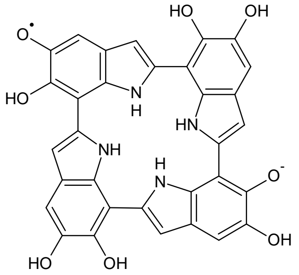 a5_b0_c6_d0	C_1, conf1	−2049.103783 (8.5)	-	-	-	-
	C_1, conf2	Evolves to C_1, conf1	-	-	-	-

Table S6. *Cont.*

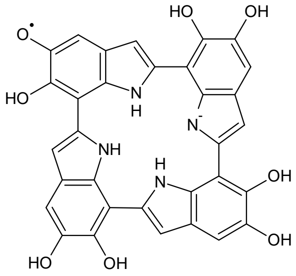 a5_b1_c0_d0	C_1, conf1	−2049.090441 (16.9)	-	-	-	-
	C_1, conf2	Evolves to C_1, conf1	-	-	-	-
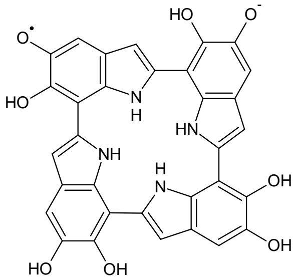 a5_b5_c0_d0	C_1, conf1	−2049.097829 (12.2)	-	-	-	-
	C_1, conf2	Evolves to C_1, conf1	-	-	-	-
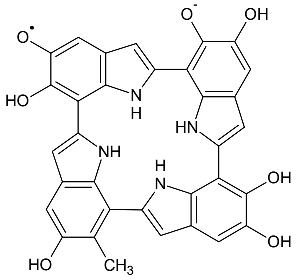 a5_b6_c0_d0	C_1, conf1	−2049.108387 (5.6)	-	-	-	-
	C_1, conf2	Evolves to C_1, conf1	-	-	-	-
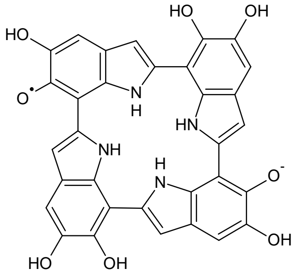 a6_b0_c6_d0	C_1, conf1	−2049.104303 (8.2)	-	-	-	-
	C_1, conf2	Evolves to C_1, conf1	-	-	-	-
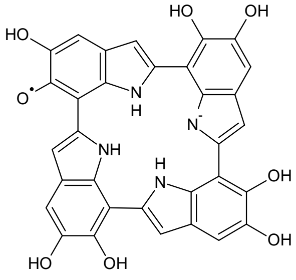 a6_b1_c0_d0	C_1, conf1	−2049.100492 (10.6)	-	-	-	-
	C_1, conf2	Evolves to C_1, conf1	-	-	-	-
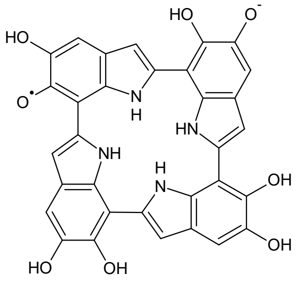 a6_b5_c0_d0	C_1, conf1	−2049.106920 (6.5)	-	-	-	-
	C_1, conf2	Evolves to C_1, conf1	-	-	-	-

Table S6. *Cont.*

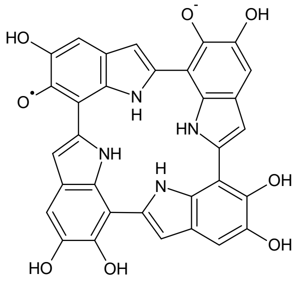 a6_b6_c0_d0	C_1, conf1	**−2049.117326** **(0.0)**	**−2048.630807** **(0.0)**	**−2048.728019** **(0.0)**	**−2049.141803** **(0.0)**	**−2048.752496** **(0.0)**

In parentheses relative energies (kcal mol^{-1}) refer to the most stable form (in bold) identified at the specified level. [a] For chiral structures, only one enantiomer is listed. [b] Electronic energy including electrostatic contributions at the polarizable continuum model (PCM) level. [c] Enthalpy computed at 298.15 K within the rigid-rotor/harmonic-oscillator (RRHO) approximation. [d] Gibbs free energy computed at 298.15 K within the RRHO approximation. [e] Electronic energy including nonelectrostatic terms according to the SMD solvation model. [f] $G_{SMD,RRHO} = G_{PCM,RRHO} + G_{SMD} - G_{PCM}$.

Table S7. KP-2e, neutral form in vacuo.

Tautomer	Conformer [a]	*E* (Ha) [b]	H_{RRHO} (Ha) [c]	G_{RRHO} (Ha) [d]
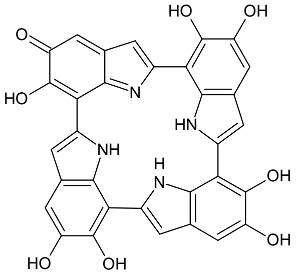 a15_b0_c0_d0	C_1, conf1	−2048.906522 (14.5)	-	-
	C_1, conf2	Evolves to C_1, conf1	-	-
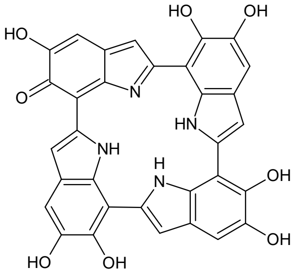 a16_b0_c0_d0	C_1, conf1	−2048.922889 (4.2)	−2048.433678 (4.0)	−2048.531488 (3.1)
	C_1, conf2	Evolves to C_1, conf1	-	-
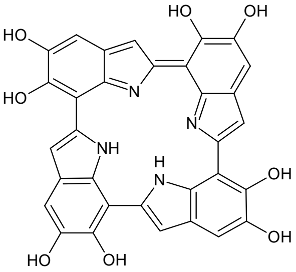 a1_b1_c0_d0	C_1, conf1	−2048.856081 (46.1) (RHF−UHF instability) −2048.870225 (37.2) (unresticted, broken symmetry)	-	-
	C_1, conf2	Evolves to C_1, conf1	-	-
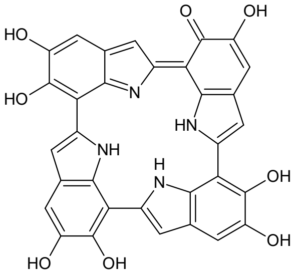 a1_b6_c0_d0	C_1, conf1	−2048.910013 (12.3)	-	-
	C_1, conf2	Evolves to C_1, conf1	-	-
	C_1, conf3	Evolves to C_1, conf1	-	-
	C_1, conf4	Evolves to C_1, conf1	-	-
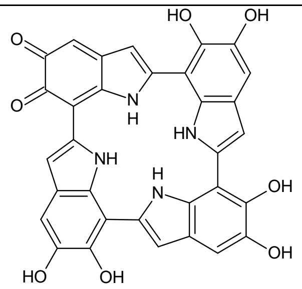 a56_b0_c0_d0	C_1, conf1	−2048.906660 (14.4)	-	-
	C_1, conf2	Evolves to C_1, conf1	-	-

Table S7. *Cont.*

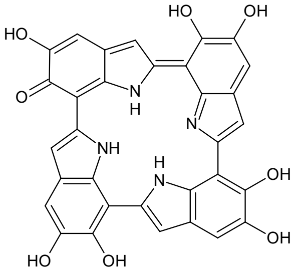 a6_b1_c0_d0	C_1, conf1	−2048.906255 (14.6)	-	-
	C_1, conf2	Evolves to C_1, conf1	-	-
	C_1, conf3	Evolves to C_1, conf1	-	-
	C_1, conf4	Evolves to C_1, conf1	-	-
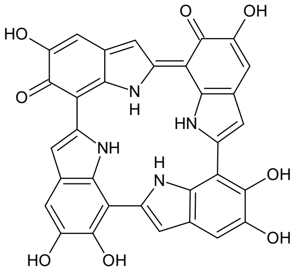 a6_b6_c0_d0	C_1, conf1	**−2048.929568 (0.0)**	**−2048.440034 (0.0)**	**−2048.536382 (0.0)**
	C_1, conf2	Evolves to C_1, conf1	-	-

In parentheses relative energies (kcal mol^{-1}) refer to the most stable form (in bold) identified at the specified level. [a] For chiral structures, only one enantiomer is listed. [b] Electronic energy. [c] Enthalpy computed at 298.15 K within the rigid-rotor/harmonic-oscillator (RRHO) approximation. [d] Gibbs free energy computed at 298.15 K within the RRHO approximation.

Table S8. KP-2e, neutral form in water.

Tautomer	Conformer [a]	G_{PCM} (Ha) [b]	$H_{PCM,RRHO}$ (Ha) [c]	$G_{PCM,RRHO}$ (Ha) [d]	G_{SMD} (Ha) [e]	$G_{SMD,RRHO}$ (Ha) [f]
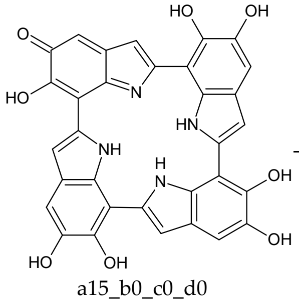 a15_b0_c0_d0	C_1, conf1	−2048.936593 (14.8)	-	-	-	-
	C_1, conf2	Evolves to C_1, conf1	-	-	-	-
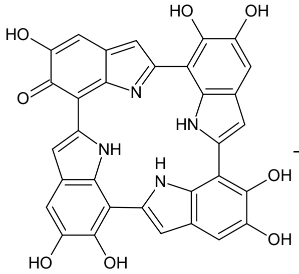 a16_b0_c0_d0	C_1, conf1	−2048.952050 (5.1)	-	-	-	-
	C_1, conf2	Evolves to C_1, conf1	-	-	-	-
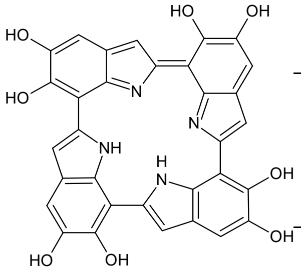 a1_b1_c0_d0	C_1, conf1	−2048.896188 (40.2)	-	-	-	-
	C_1, conf2	Evolves to C_1, conf1	-	-	-	-
	C_1, conf3	Evolves to C_1, conf1	-	-	-	-
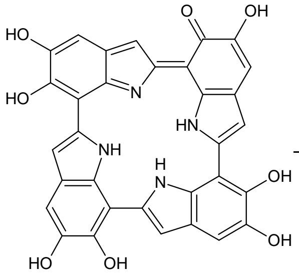 a1_b6_c0_d0	C_1, conf1	−2048.940431 (12.4)	-	-	-	-
	C_1, conf2	Evolves to C_1, conf1	-	-	-	-
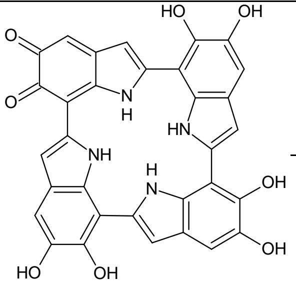 a56_b0_c0_d0	C_1, conf1	−2048.947596 (7.9)	-	-	-	-
	C_1, conf2	Evolves to C_1, conf1	-	-	-	-

Table S8. *Cont.*

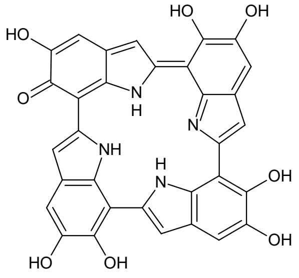 a6_b1_c0_d0	C_1, conf1	−2048.937889 (14.0)	-	-	-	-
	C_1, conf2	Evolves to C_1, conf1	-	-	-	-
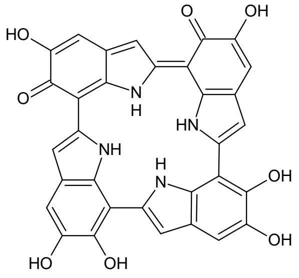 a6_b6_c0_d0	C_1, conf1	**−2048.960200 (0.0)**	**−2048.472201 (0.0)**	**−2048.569202 (0.0)**	**−2048.984556 (0.0)**	**−2048.593558 (0.0)**
	C_1, conf2	Evolves to C_1, conf1	-	-	-	-

In parentheses relative energies (kcal mol^{-1}) refer to the most stable form (in bold) identified at the specified level. [a] For chiral structures, only one enantiomer is listed. [b] Electronic energy including electrostatic contributions at the polarizable continuum model (PCM) level. [c] Enthalpy computed at 298.15 K within the rigid-rotor/harmonic-oscillator (RRHO) approximation. [d] Gibbs free energy computed at 298.15 K within the RRHO approximation. [e] Electronic energy including nonelectrostatic terms according to the SMD solvation model. [f] $G_{SMD,RRHO} = G_{PCM,RRHO} + G_{SMD} - G_{PCM}$.

Table S9. KP-2e, monoanionic form in water.

Tautomer	Conformer [a]	G_{PCM} (Ha) [b]	$H_{\text{PCM,RRHO}}$ (Ha) [c]	$G_{\text{PCM,RRHO}}$ (Ha) [d]	G_{SMD} (Ha) [e]	$G_{\text{SMD,RRHO}}$ (Ha) [f]
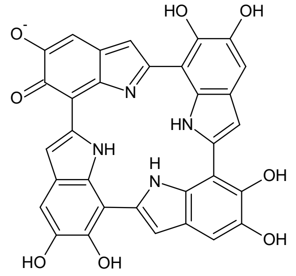 a156_b0_c0_d0	C_1, conf1	−2048.482452 (11.7)	-	-	-	-
	C_1, conf2	Evolves to C_1, conf1	-	-	-	-
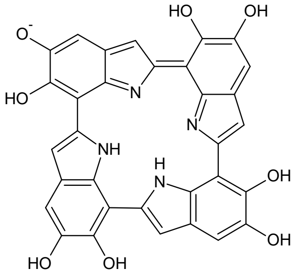 a15_b1_c0_d0	C_1, conf1	−2048.452133 (30.7)	-	-	-	-
	C_1, conf2	Evolves to C_1, conf1	-	-	-	-
	C_1, conf3	Evolves to C_1, conf1	-	-	-	-
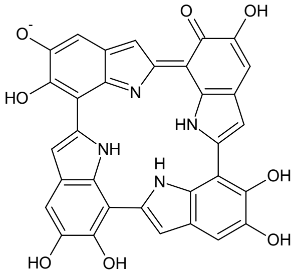 a15_b6_c0_d0	C_1, conf1	−2048.487906 (8.3)	-	-	-	-
	C_1, conf2	Evolves to C_1, conf1	-	-	-	-
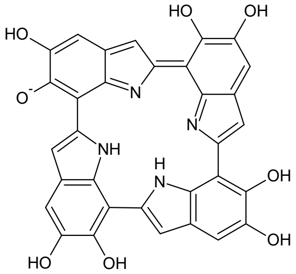 a16_b1_c0_d0	C_1, conf1	−2048.468071 (20.7)	-	-	-	-
	C_1, conf2	Evolves to C_1, conf1	-	-	-	-
	C_1, conf3	Evolves to C_1, conf1	-	-	-	-
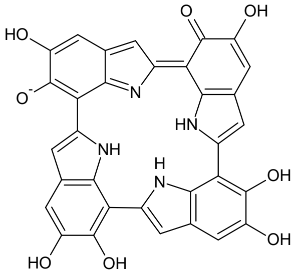 a16_b6_c0_d0	C_1, conf1	**−2048.501057 (0.0)**	**−2048.026967 (0.0)**	**−2048.123602 (0.0)**	**−2048.520660 (0.0)**	**−2048.143205 (0.5)**
	C_1, conf2	Evolves to C_1, conf1	-	-	-	-

Table S9. *Cont.*

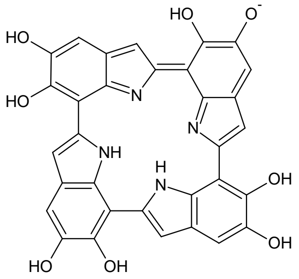 a1_b15_c0_d0	C_1, conf1	−2048.452485 (30.5)	-	-	-	-
	C_1, conf2	Evolves to C_1, conf1	-	-	-	-
	C_1, conf3	Evolves to C_1, conf1	-	-	-	-
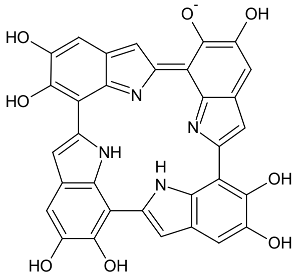 a1_b16_c0_d0	C_1, conf1	−2048.470317 (19.3)	-	-	-	-
	C_1, conf2	Evolves to C_1, conf1	-	-	-	-
	C_1, conf3	Evolves to C_1, conf1	-	-	-	-
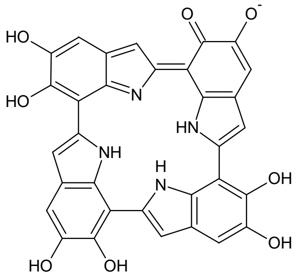 a1_b56_c0_d0	C_1, conf1	−2048.473653 (17.2)	-	-	-	-
	C_1, conf2	Evolves to C_1, conf1	-	-	-	-
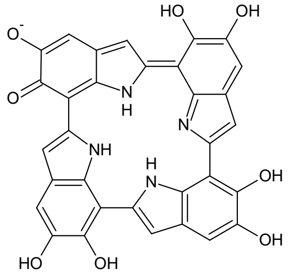 a56_b1_c0_d0	C_1, conf1	−2048.475514 (16.0)	-	-	-	-
	C_1, conf2	Evolves to C_1, conf1	-	-	-	-
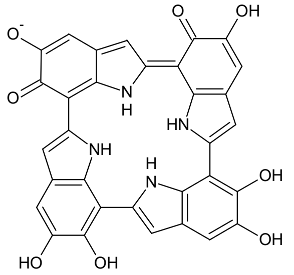 a56_b6_c0_d0	C_1, conf1	−2048.494742 (4.0)	−2048.020572 (4.0)	−2048.118108 (3.4)	−2048.520680 (0.0)	−2048.144046 (0.0)
	C_1, conf2	Evolves to C_1, conf1	-	-	-	-

Table S9. *Cont.*

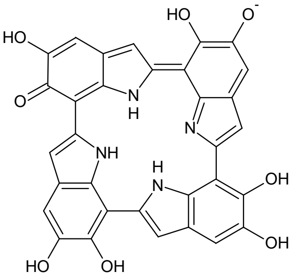 a6_b15_c0_d0	C_1, conf1	−2048.477324 (14.9)	-	-	-	-
	C_1, conf2	Evolves to C_1, conf1	-	-	-	-
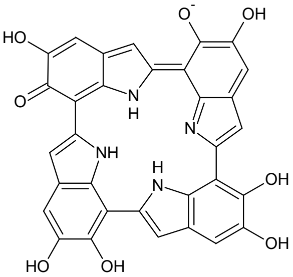 a6_b16_c0_d0	C_1, conf1	−2048.497643 (2.1)	−2048.023707 (2.0)	−2048.120613 (1.9)	−2048.517642 (1.9)	−2048.140612 (2.2)
	C_1, conf2	Evolves to C_1, conf1	-	-	-	-
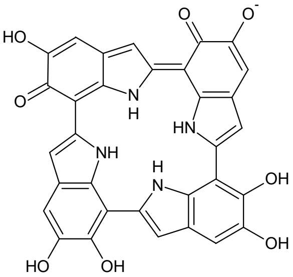 a6_b56_c0_d0	C_1, conf1	−2048.487298 (8.6)	-	-	-	-
	C_1, conf2	Evolves to C_1, conf1	-	-	-	-

In parentheses relative energies (kcal mol^{-1}) refer to the most stable form (in bold) identified at the specified level. [a] For chiral structures, only one enantiomer is listed. [b] Electronic energy including electrostatic contributions at the polarizable continuum model (PCM) level. [c] Enthalpy computed at 298.15 K within the rigid-rotor/harmonic-oscillator (RRHO) approximation. [d] Gibbs free energy computed at 298.15 K within the RRHO approximation. [e] Electronic energy including nonelectrostatic terms according to the SMD solvation model. [f] $G_{SMD,RRHO} = G_{PCM,RRHO} + G_{SMD} - G_{PCM}$.

Table S10. KP-4e, neutral form in vacuo.

Tautomer	Conformer [a]	*E* (Ha) [b]	H_{RRHO} (Ha) [c]	G_{RRHO} (Ha) [d]
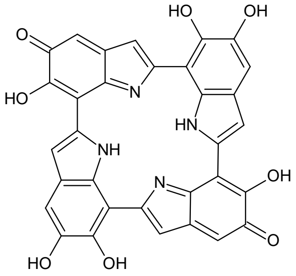 a15_b0_c15_d0	C_1, conf1	−2047.659734 (26.6)	-	-
	C_1, conf2	Evolves to C_1, conf1	-	-
	C_2, conf1	−2047.659607 (26.7)	-	-
	C_2, conf2	Evolves to C_2, conf1	-	-
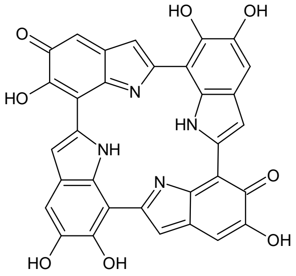 a15_b0_c16_d0	C_1, conf1	−2047.675979 (16.4)	-	-
	C_1, conf2	Evolves to C_1, conf1	-	-
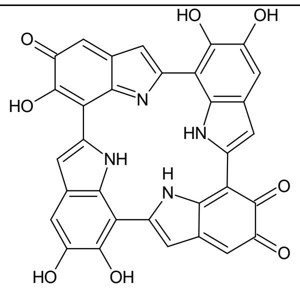 a15_b0_c56_d0	C_1, conf1	−2047.657991 (27.7)	-	-
	C_1, conf2	Evolves to C_1, conf1	-	-
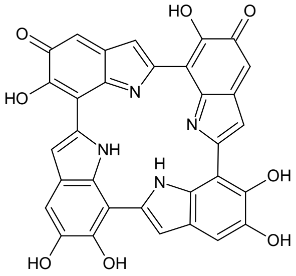 a15_b15_c0_d0	C_1, conf1	−2047.647562 (34.3)	-	-
	C_1, conf2	Evolves to C_1, conf1	-	-
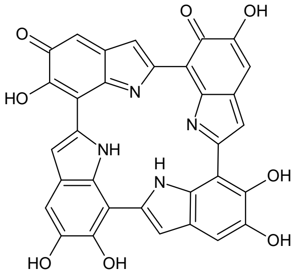 a15_b16_c0_d0	C_1, conf1	−2047.661078 (25.8)	-	-
	C_1, conf2	Evolves to C_1, conf1	-	-

Table S10. *Cont.*

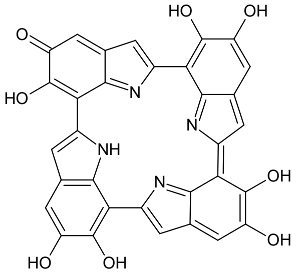 a15_b1_c1_d0	C_1, conf1	−2047.591879 (69.2)	-	-
	C_1, conf2	Evolves to C_1, conf1	-	-
	C_1, conf3	Evolves to C_1, conf1	-	-
	C_1, conf4	−2047.591935 (69.2)	-	-
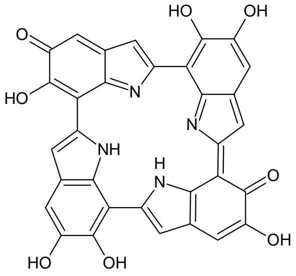 a15_b1_c6_d0	C_1, conf1	−2047.653159 (30.7)	-	-
	C_1, conf2	Evolves to C_1, conf1	-	-
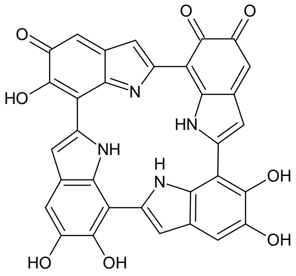 a15_b56_c0_d0	C_1, conf1	−2047.659044 (27.1)	-	-
	C_1, conf2	Evolves to C_1, conf1	-	-
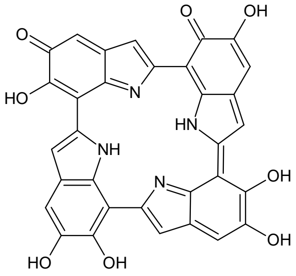 a15_b6_c1_d0	C_1, conf1	−2047.657784 (27.8)	-	-
	C_1, conf2	Evolves to C_1, conf1	-	-
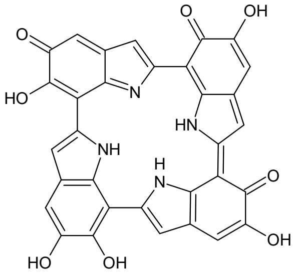 a15_b6_c6_d0	C_1, conf1	−2047.684806 (10.9)	-	-
	C_1, conf2	Evolves to C_1, conf1	-	-
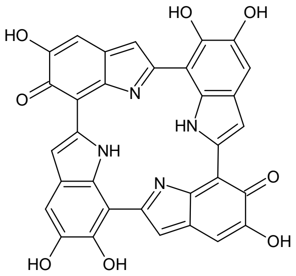 a16_b0_c16_d0	C_1, conf1	−2047.692169 (6.3)	-	-
	C_1, conf2	Evolves to C_1, conf1	-	-
	C_2, conf1	−2047.692054 (6.3)	-	-

Table S10. *Cont.*

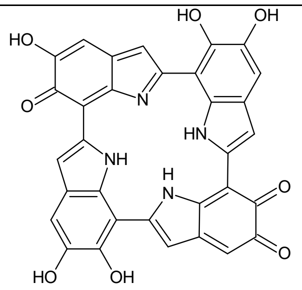 a16_b0_c56_d0	C_1, conf1	−2047.673763 (17.8)	-	-
	C_1, conf2	Evolves to C_1, conf1	-	-
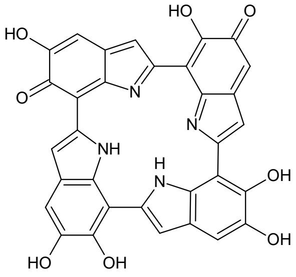 a16_b15_c0_d0	C_1, conf1	−2047.663293 (24.4)	-	-
	C_1, conf2	Evolves to C_1, conf1	-	-
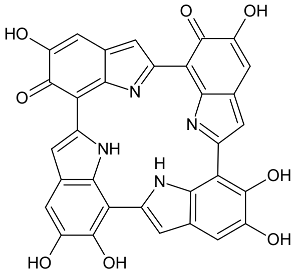 a16_b16_c0_d0	C_1, conf1	−2047.674752 (17.2)	-	-
	C_1, conf2	Evolves to C_1, conf1	-	-
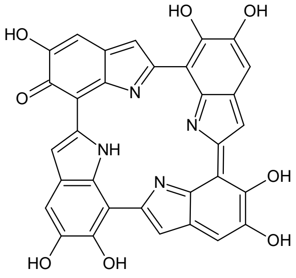 a16_b1_c1_d0	C_1, conf1	−2047.605478 (60.7)	-	-
	C_1, conf2	Evolves to C_1, conf1	-	-
	C_1, conf3	Evolves to C_1, conf1	-	-
	C_1, conf4	−2047.605528 (60.6)	-	-
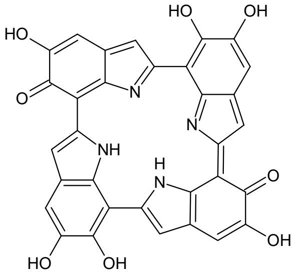 a16_b1_c6_d0	C_1, conf1	−2047.668923 (20.9)	-	-
	C_1, conf2	Evolves to C_1, conf1	-	-
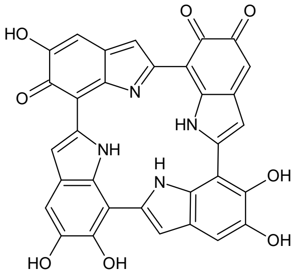 a16_b56_c0_d0	C_1, conf1	−2047.675354 (16.8)	-	-
	C_1, conf2	Evolves to C_1, conf1	-	-

Table S10. *Cont.*

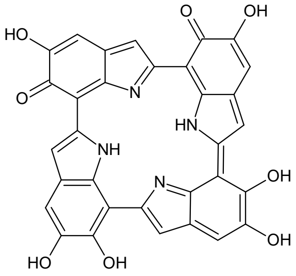 a16_b6_c1_d0	C_1, conf1	−2047.675395 (16.8)	-	-
	C_1, conf2	Evolves to C_1, conf1	-	-
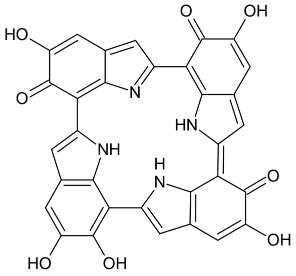 a16_b6_c6_d0	C_1, conf1	−2047.700139 (1.3)	−2047.235705 (1.2)	−2047.331297 (1.1)
	C_1, conf2	Evolves to C_1, conf1	-	-
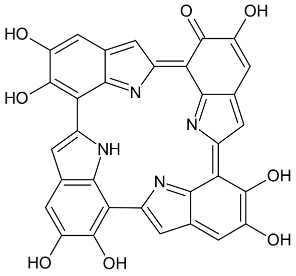 a1_b16_c1_d0	C_1, conf1	−2047.600627 (63.7)	-	-
	C_1, conf2	Evolves to C_1, conf1	-	-
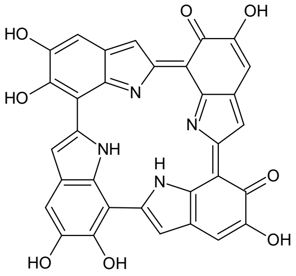 a1_b16_c6_d0	C_1, conf1	−2047.663803 (24.1)	-	-
	C_1, conf2	Evolves to C_1, conf1	-	-
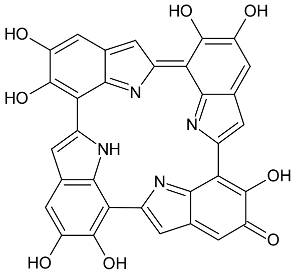 a1_b1_c15_d0	C_1, conf1	−2047.593839 (68.0)	-	-
	C_1, conf2	Evolves to C_1, conf1	-	-
	C_1, conf3	Evolves to C_1, conf1	-	-
	C_1, conf4	Evolves to C_1, conf1	-	-
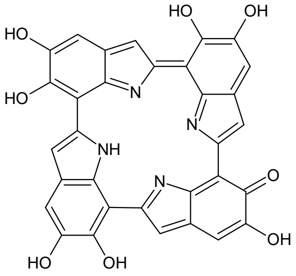 a1_b1_c16_d0	C_1, conf1	−2047.607402 (59.5)	-	-
	C_1, conf2	Evolves to C_1, conf1	-	-
	C_1, conf3	Evolves to C_1, conf1	-	-
	C_1, conf4	Evolves to C_1, conf1	-	-

Table S10. *Cont.*

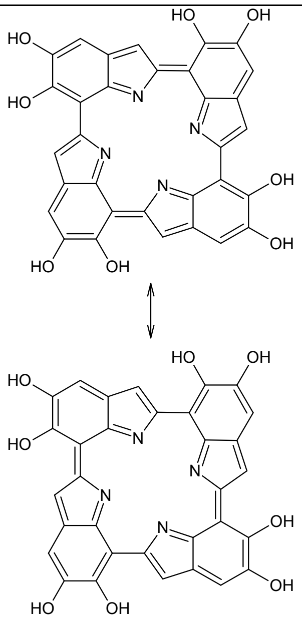 a1_b1_c1_d1	C_1, conf1	−2047.509613 (120.8)	-	-
	C_1, conf2	Evolves to C_1, conf1	-	-
	C_1, conf3	Evolves to C_1, conf1	-	-
	C_1, conf4	Evolves to C_1, conf1	-	-
	C_2, conf1	−2047.509485 (120.9)	-	-
	S_4, conf1	Evolves to C_2, conf1	-	-
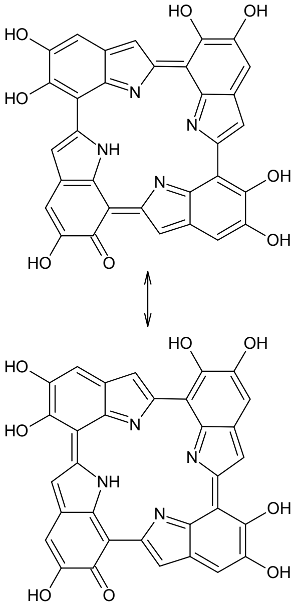 a1_b1_c1_d6	C_1, conf1	−2047.594386 (67.6)	-	-
	C_1, conf2	Evolves to C_1, conf1	-	-
	C_1, conf3	Evolves to C_1, conf1	-	-
	C_1, conf4	Evolves to C_1, conf1	-	-
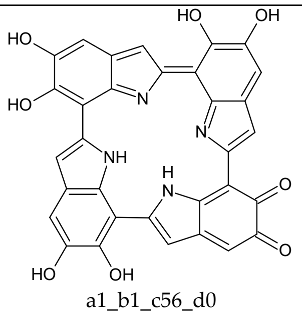 a1_b1_c56_d0	C_1, conf1	−2047.612442 (56.3)	-	-
	C_1, conf2	Evolves to C_1, conf1	-	-
	C_1, conf3	Evolves to C_1, conf1	-	-
	C_1, conf4	Evolves to C_1, conf1	-	-

Table S10. *Cont.*

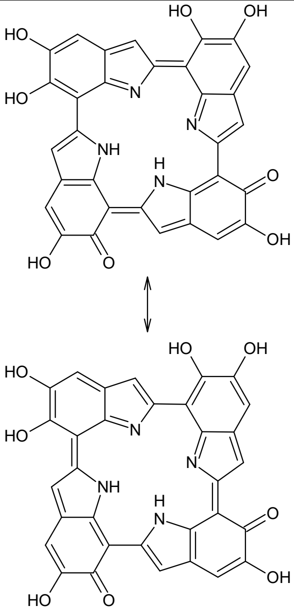	C_1, conf1	−2047.652726 (31.0)	-	-
	C_1, conf2	Evolves to C_1, conf1	-	-
	C_1, conf3	Evolves to C_1, conf1	-	-
a1_b1_c6_d6	C_1, conf4	Evolves to C_1, conf1	-	-
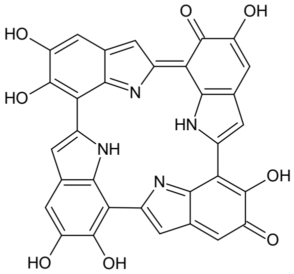	C_1, conf1	−2047.664002 (23.9)	-	-
a1_b6_c15_d0	C_1, conf2	Evolves to C_1, conf1	-	-
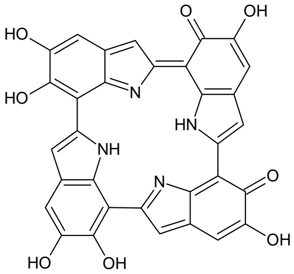	C_1, conf1	−2047.679623 (14.1)	-	-
a1_b6_c16_d0	C_1, conf2	Evolves to C_1, conf1	-	-

Table S10. *Cont.*

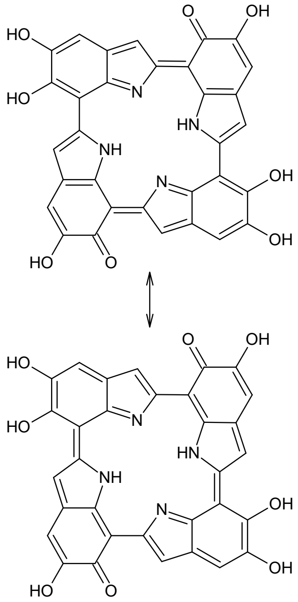 a1_b6_c1_d6	C_1, conf1	−2047.676296 (16.2)	-	-
	C_1, conf2	Evolves to C_1, conf1	-	-
	C_2, conf1	−2047.676081 (16.4)	-	-
	C_2, conf2	Evolves to C_2, conf1	-	-
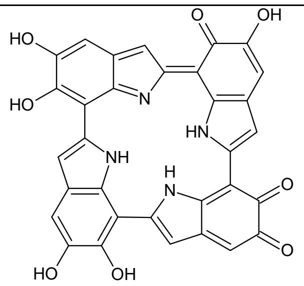 a1_b6_c56_d0	C_1, conf1	−2047.662061 (25.2)	-	-
	C_1, conf2	Evolves to C_1, conf1	-	-
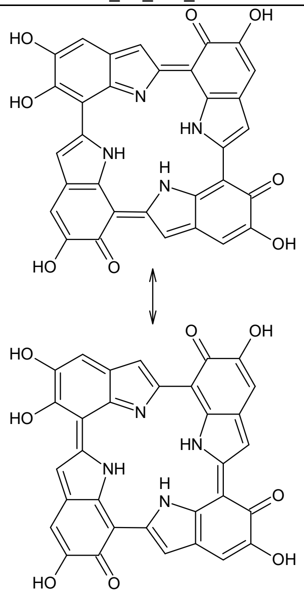 a1_b6_c6_d6	C_1, conf1	−2047.691396 (6.8)	-	-
	C_1, conf2	Evolves to C_1, conf1	-	-

Table S10. *Cont.*

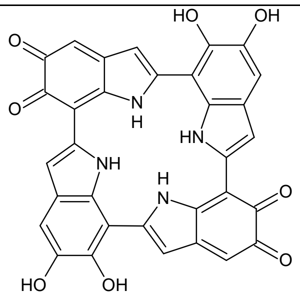 a56_b0_c56_d0	C_1, conf1	−2047.656227 (28.8)	-	-
	C_1, conf2	Evolves to C_1, conf1	-	-
	C_2, conf1	−2047.656138 (28.9)	-	-
	C_2, conf2	Evolves to C_2, conf1	-	-
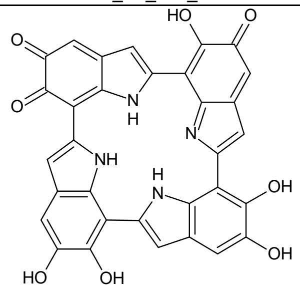 a56_b15_c0_d0	C_1, conf1	−2047.654504 (29.9)	-	-
	C_1, conf2	Evolves to C_1, conf1	-	-
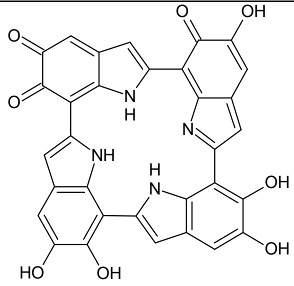 a56_b16_c0_d0	C_1, conf1	−2047.668558 (21.1)	-	-
	C_1, conf2	Evolves to C_1, conf1	-	-
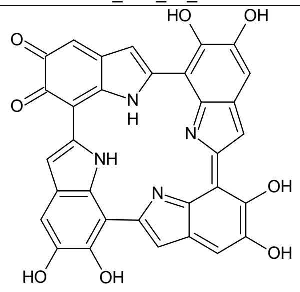 a56_b1_c1_d0	C_1, conf1	−2047.607910 (59.1)	-	-
	C_1, conf2	−2047.608930 (58.5)	-	-
	C_1, conf3	Evolves to C_1, conf1	-	-
	C_1, conf4	Evolves to C_1, conf2	-	-
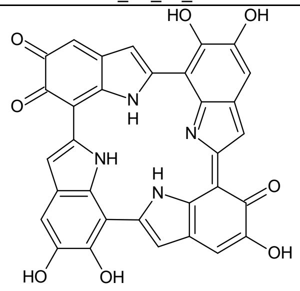 a56_b1_c6_d0	C_1, conf1	−2047.662068 (25.2)	-	-
	C_1, conf2	Evolves to C_1, conf1	-	-

Table S10. *Cont.*

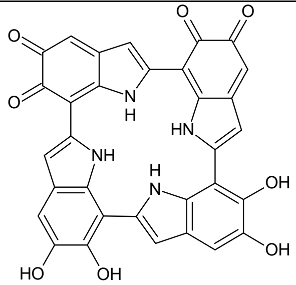 a56_b56_c0_d0	C_1, conf1	−2047.654721 (29.8)	-	-
	C_1, conf2	Evolves to C_1, conf1	-	-
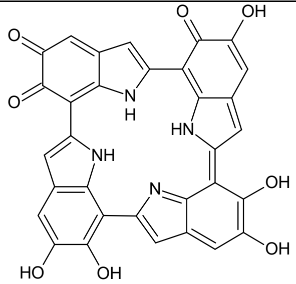 a56_b6_c1_d0	C_1, conf1	−2047.652373 (31.2)	-	-
	C_1, conf2	Evolves to C_1, conf1	-	-
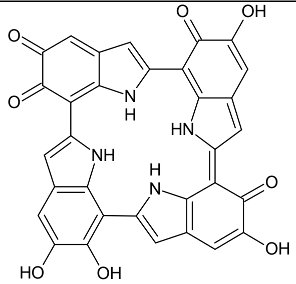 a56_b6_c6_d0	C_1, conf1	−2047.677799 (15.3)	-	-
	C_1, conf2	Evolves to C_1, conf1	-	-
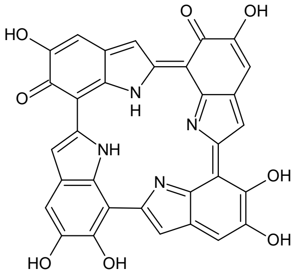 a6_b16_c1_d0	C_1, conf1	−2047.663156 (24.5)	-	-
	C_1, conf2	Evolves to C_1, conf1	-	-
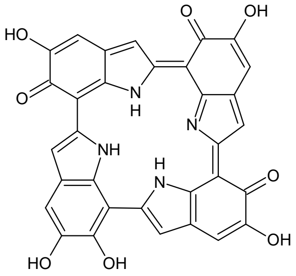 a6_b16_c6_d0	C_1, conf1	**−2047.702153 (0.0)**	**−2047.237674 (0.0)**	**−2047.333050 (0.0)**
	C_1, conf2	Evolves to C_1, conf1	-	-

Table S10. *Cont.*

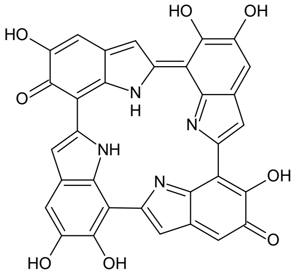 a6_b1_c15_d0	C_1, conf1	−2047.652884 (30.9)	-	-
	C_1, conf2	Evolves to C_1, conf1	-	-
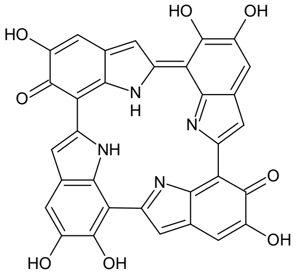 a6_b1_c16_d0	C_1, conf1	−2047.668512 (21.1)	-	-
	C_1, conf2	Evolves to C_1, conf1	-	-
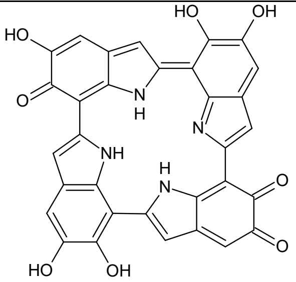 a6_b1_c56_d0	C_1, conf1	−2047.658487 (27.4)	-	-
	C_1, conf2	Evolves to C_1, conf1	-	-
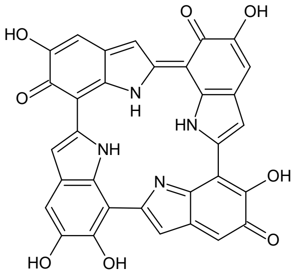 a6_b6_c15_d0	C_1, conf1	−2047.682897 (12.1)	-	-
	C_1, conf2	Evolves to C_1, conf1	-	-
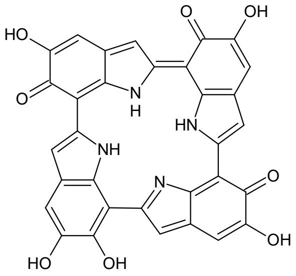 a6_b6_c16_d0	C_1, conf1	−2047.699471 (1.7)	−2047.234863 (1.8)	−2047.330241 (1.8)
	C_1, conf2	Evolves to C_1, conf1	-	-
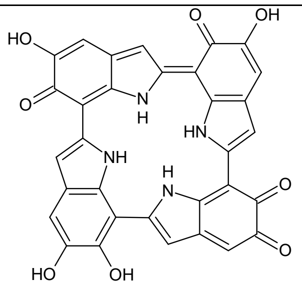 a6_b6_c56_d0	C_1, conf1	−2047.678495 (14.8)	-	-
	C_1, conf2	Evolves to C_1, conf1	-	-

Table S10. *Cont.*

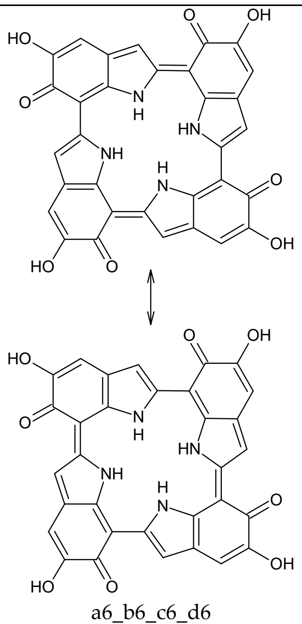	C_1, conf1	−2047.696161 (3.8)	−2047.231453 (3.9)	−2047.325734 (4.6)
	C_1, conf2	Evolves to C_1, conf1	-	-
	C_2, conf1	−2047.696144 (3.8)	−2047.231368 (4.0)	−2047.324833 (5.2)
	S_4, conf1	Evolves to C_2, conf1	-	-
a6_b6_c6_d6	C_4, conf1	Evolves to C_2, conf1	-	-

In parentheses relative energies (kcal mol^{-1}) refer to the most stable form (in bold) identified at the specified level. [a] For chiral structures, only one enantiomer is listed. [b] Electronic energy. [c] Enthalpy computed at 298.15 K within the rigid-rotor/harmonic-oscillator (RRHO) approximation. [d] Gibbs free energy computed at 298.15 K within the RRHO approximation.

Table S11. KP-4e, neutral form in water.

Tautomer	Conformer [a]	G_{PCM} (Ha) [b]	$H_{PCM,RRHO}$ (Ha) [c]	$G_{PCM,RRHO}$ (Ha) [d]	G_{SMD} (Ha) [e]	$G_{SMD,RRHO}$ (Ha) [f]
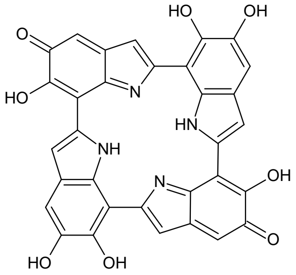 a15_b0_c15_d0	C_1, conf1	−2047.684632 (25.3)	-	-	-	-
	C_1, conf2	Evolves to C_1, conf1	-	-	-	-
	C_2, conf1	−2047.684450 (25.4)	-	-	-	-
	C_2, conf2	Evolves to C_2, conf1	-	-	-	-
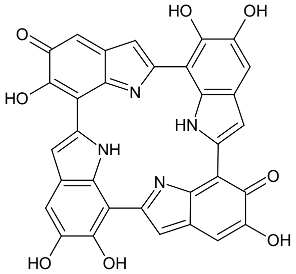 a15_b0_c16_d0	C_1, conf1	−2047.700685 (15.2)	-	-	-	-
	C_1, conf2	Evolves to C_1, conf1	-	-	-	-
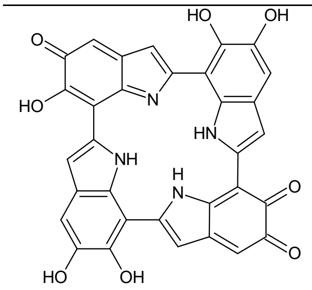 a15_b0_c56_d0	C_1, conf1	−2047.693100 (20.0)	-	-	-	-
	C_1, conf2	Evolves to C_1, conf1	-	-	-	-
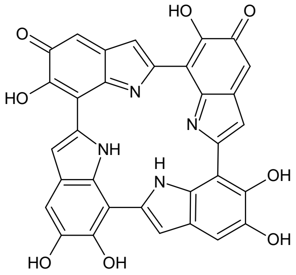 a15_b15_c0_d0	C_1, conf1	−2047.673587 (32.2)	-	-	-	-
	C_1, conf2	Evolves to C_1, conf1	-	-	-	-
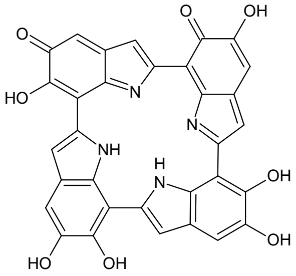 a15_b16_c0_d0	C_1, conf1	−2047.686687 (24.0)	-	-	-	-
	C_1, conf2	Evolves to C_1, conf1	-	-	-	-

Table S11. *Cont.*

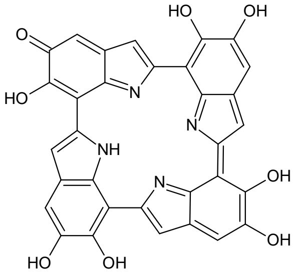 a15_b1_c1_d0	C_1, conf1	−2047.631792 (58.4)	-	-	-	-
	C_1, conf2	Evolves to C_1, conf1	-	-	-	-
	C_1, conf3	Evolves to C_1, conf1	-	-	-	-
	C_1, conf4	Evolves to C_1, conf1	-	-	-	-
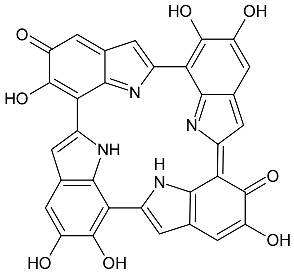 a15_b1_c6_d0	C_1, conf1	−2047.679840 (28.3)	-	-	-	-
	C_1, conf2	Evolves to C_1, conf1	-	-	-	-
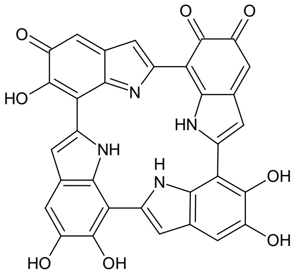 a15_b56_c0_d0	C_1, conf1	−2047.692672 (20.2)	-	-	-	-
	C_1, conf2	Evolves to C_1, conf1	-	-	-	-
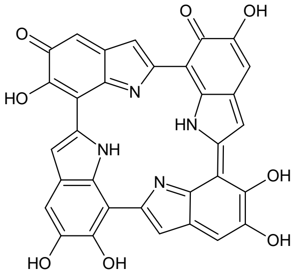 a15_b6_c1_d0	C_1, conf1	−2047.684216 (25.5)	-	-	-	-
	C_1, conf2	Evolves to C_1, conf1	-	-	-	-
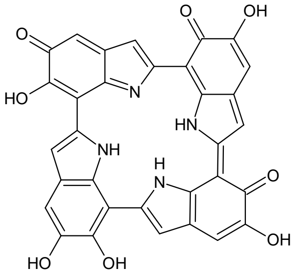 a15_b6_c6_d0	C_1, conf1	−2047.708533 (10.3)	-	-	-	-
	C_1, conf2	Evolves to C_1, conf1	-	-	-	-
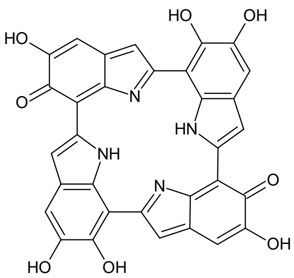 a16_b0_c16_d0	C_1, conf1	−2047.716730 (5.1)	-	-	-	-
	C_1, conf2	Evolves to C_1, conf1	-	-	-	-
	C_2, conf1	−2047.716589 (5.2)	-	-	-	-

Table S11. *Cont.*

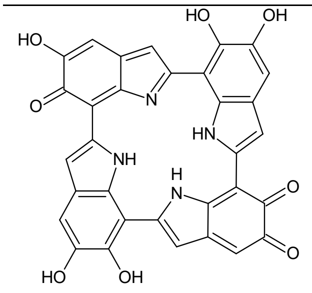 a16_b0_c56_d0	C_1, conf1	−2047.708017 (10.6)	-	-	-	-
	C_1, conf2	Evolves to C_1, conf1	-	-	-	-
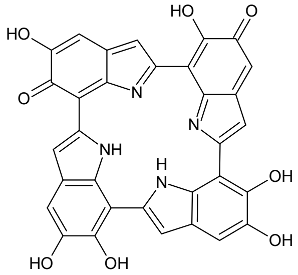 a16_b15_c0_d0	C_1, conf1	−2047.688825 (22.6)	-	-	-	-
	C_1, conf2	Evolves to C_1, conf1	-	-	-	-
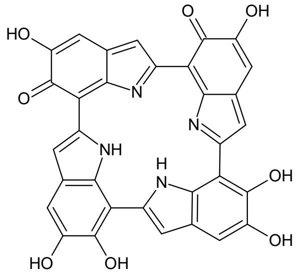 a16_b16_c0_d0	C_1, conf1	−2047.700117 (15.6)	-	-	-	-
	C_1, conf2	Evolves to C_1, conf1	-	-	-	-
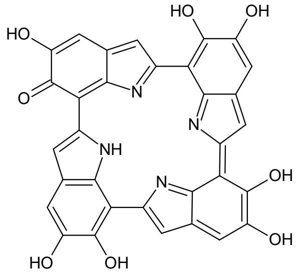 a16_b1_c1_d0	C_1, conf1	−2047.645704 (49.7)	-	-	-	-
	C_1, conf2	Evolves to C_1, conf1	-	-	-	-
	C_1, conf3	Evolves to C_1, conf1	-	-	-	-
	C_1, conf4	−2047.645523 (49.8)	-	-	-	-
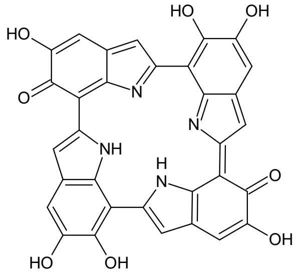 a16_b1_c6_d0	C_1, conf1	−2047.695687 (18.3)	-	-	-	-
	C_1, conf2	Evolves to C_1, conf1	-	-	-	-
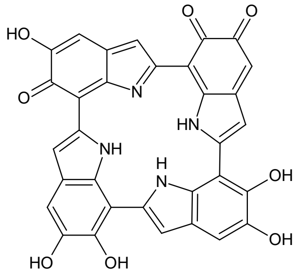 a16_b56_c0_d0	C_1, conf1	−2047.708327 (10.4)	-	-	-	-
	C_1, conf2	Evolves to C_1, conf1	-	-	-	-

Table S11. *Cont.*

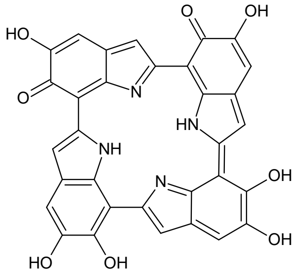 a16_b6_c1_d0	C_1, conf1	−2047.700766 (15.1)	-	-	-	-
	C_1, conf2	Evolves to C_1, conf1	-	-	-	-
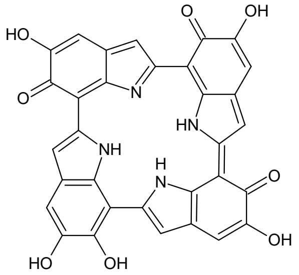 a16_b6_c6_d0	C_1, conf1	−2047.722869 (1.3)	−2047.260061 (1.3)	−2047.355968 (1.4)	−2047.741845 (1.6)	−2047.374944 (1.5)
	C_1, conf2	Evolves to C_1, conf1	-	-	-	-
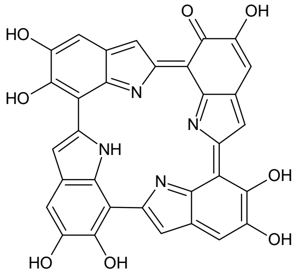 a1_b16_c1_d0	C_1, conf1	−2047.639492 (53.6)	-	-	-	-
	C_1, conf2	Evolves to C_1, conf1	-	-	-	-
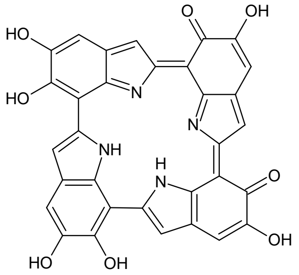 a1_b16_c6_d0	C_1, conf1	−2047.690404 (21.6)	-	-	-	-
	C_1, conf2	Evolves to C_1, conf1	-	-	-	-
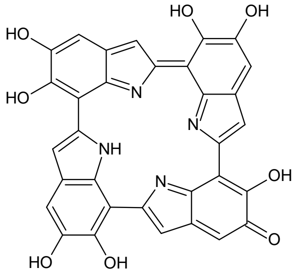 a1_b1_c15_d0	C_1, conf1	−2047.632813 (57.8)	-	-	-	-
	C_1, conf2	Evolves to C_1, conf1	-	-	-	-
	C_1, conf3	Evolves to C_1, conf1	-	-	-	-
	C_1, conf4	Evolves to C_1, conf1	-	-	-	-
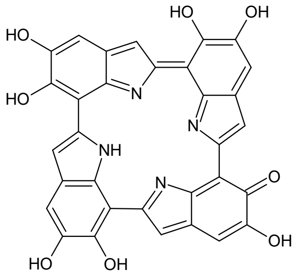 a1_b1_c16_d0	C_1, conf1	−2047.647321 (48.7)	-	-	-	-
	C_1, conf2	Evolves to C_1, conf1	-	-	-	-
	C_1, conf3	Evolves to C_1, conf1	-	-	-	-
	C_1, conf4	Evolves to C_1, conf1	-	-	-	-

Table S11. *Cont.*

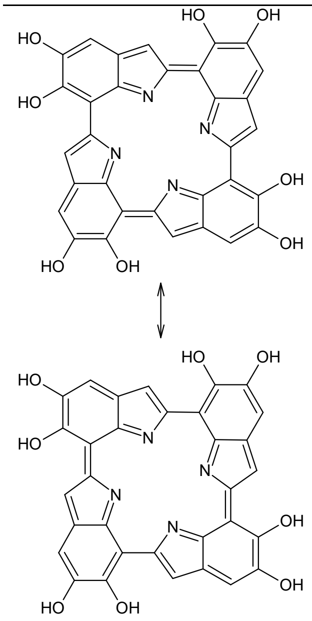	C_1, conf1	−2047.572925 (95.4)	-	-	-	-
	C_1, conf2	−2047.573059 (95.3)	-	-	-	-
	C_1, conf3	Evolves to C_1, conf2	-	-	-	-
	C_1, conf4	Evolves to C_1, conf2	-	-	-	-
	C_2, conf1	−2047.572958 (95.3)	-	-	-	-
a1_b1_c1_d1	S_4, conf1	Evolves to C_2, conf1	-	-	-	-
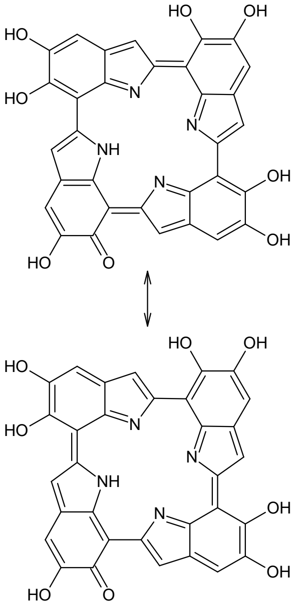	C_1, conf1	−2047.635698 (56.0)	-	-	-	-
	C_1, conf2	Evolves to C_1, conf1	-	-	-	-
	C_1, conf3	Evolves to C_1, conf1	-	-	-	-
a1_b1_c1_d6	C_1, conf4	Evolves to C_1, conf1	-	-	-	-
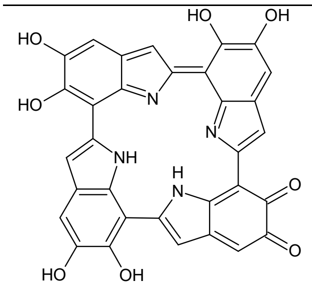	C_1, conf1	−2047.656074 (43.2)	-	-	-	-
	C_1, conf2	Evolves to C_1, conf1	-	-	-	-
	C_1, conf3	Evolves to C_1, conf1	-	-	-	-
a1_b1_c56_d0	C_1, conf4	Evolves to C_1, conf1	-	-	-	-

Table S11. *Cont.*

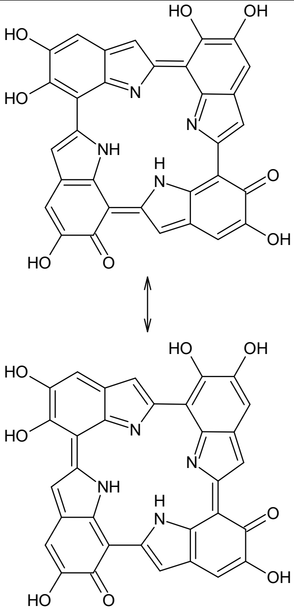	C_1, conf1	−2047.674939 (31.4)	-	-	-	-
	C_1, conf2	Evolves to C_1, conf1	-	-	-	-
	C_1, conf3	Evolves to C_1, conf1	-	-	-	-
a1_b1_c6_d6	C_1, conf4	Evolves to C_1, conf1	-	-	-	-
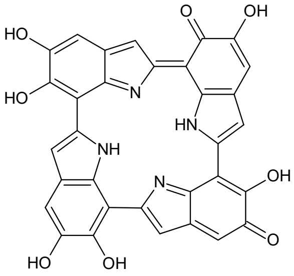	C_1, conf1	−2047.689599 (22.2)				
a1_b6_c15_d0	C_1, conf2	Evolves to C_1, conf1	-	-	-	-
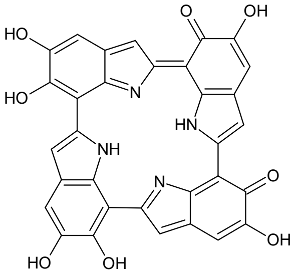	C_1, conf1	−2047.704878 (12.6)	-	-	-	-
a1_b6_c16_d0	C_1, conf2	Evolves to C_1, conf1	-	-	-	-

Table S11. *Cont.*

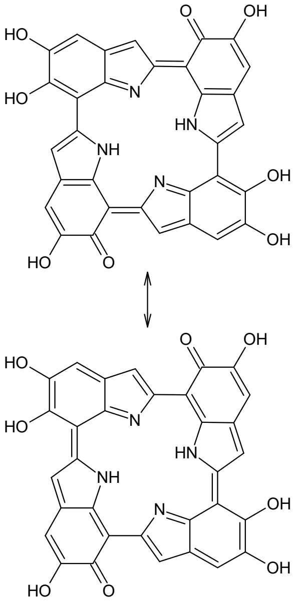	C_1, conf1	−2047.702310 (14.2)	-	-	-	-
	C_1, conf2	Evolves to C_1, conf1	-	-	-	-
	C_2, conf1	−2047.702182 (14.3)	-	-	-	-
a1_b6_c1_d6	C_2, conf2	Evolves to C_2, conf1	-	-	-	-
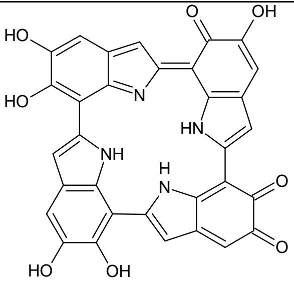	C_1, conf1	−2047.696154 (18.0)	-	-	-	-
a1_b6_c56_d0	C_1, conf2	Evolves to C_1, conf1	-	-	-	-
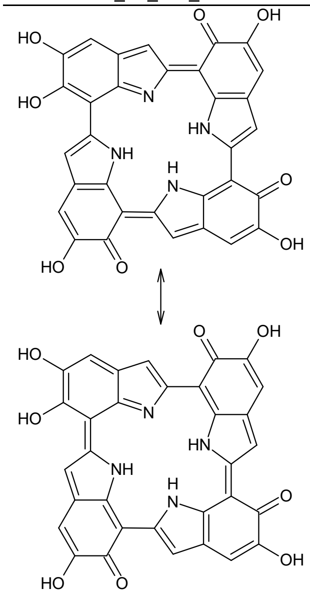	C_1, conf1	−2047.714953 (6.2)	-	-	-	-
a1_b6_c6_d6	C_1, conf2	Evolves to C_1, conf1	-	-	-	-

Table S11. *Cont.*

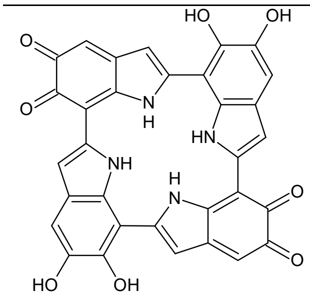 a56_b0_c56_d0	C_1, conf1	−2047.702924 (13.8)	-	-	-	-
	C_1, conf2	Evolves to C_1, conf1	-	-	-	-
	C_2, conf1	−2047.702974 (13.8)	-	-	-	-
	C_2, conf2	Evolves to C_2, conf1	-	-	-	-
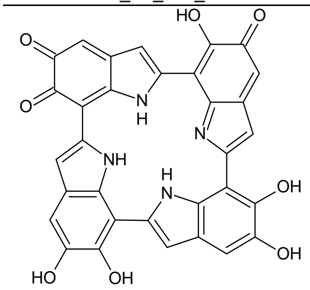 a56_b15_c0_d0	C_1, conf1	−2047.690061 (21.9)	-	-	-	-
	C_1, conf2	Evolves to C_1, conf1	-	-	-	-
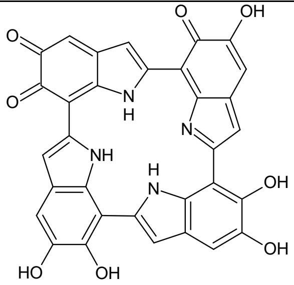 a56_b16_c0_d0	C_1, conf1	−2047.702841 (13.8)	-	-	-	-
	C_1, conf2	Evolves to C_1, conf1	-	-	-	-
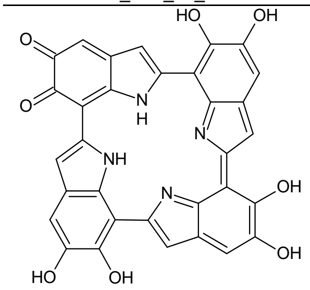 a56_b1_c1_d0	C_1, conf1	−2047.654385 (44.2)	-	-	-	-
	C_1, conf2	Evolves to C_1, conf1	-	-	-	-
	C_1, conf3	Evolves to C_1, conf1	-	-	-	-
	C_1, conf4	Evolves to C_1, conf2	-	-	-	-
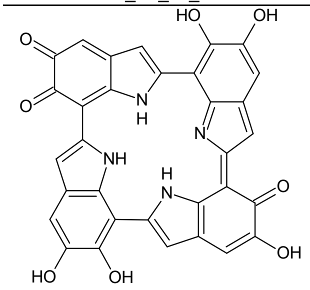 a56_b1_c6_d0	C_1, conf1	−2047.698292 (16.7)	-	-	-	-
	C_1, conf2	Evolves to C_1, conf1	-	-	-	-

Table S11. *Cont.*

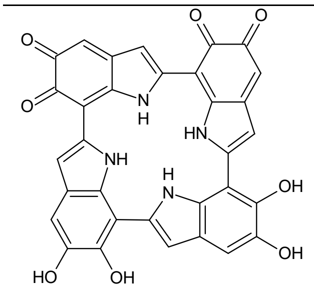 a56_b56_c0_d0	C_1, conf1	−2047.701202 (14.9)	-	-	-	-
	C_1, conf2	Evolves to C_1, conf1	-	-	-	-
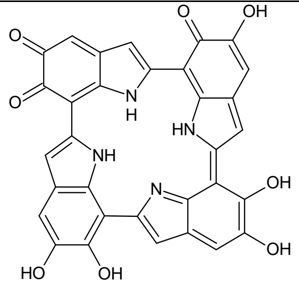 a56_b6_c1_d0	C_1, conf1	−2047.689301 (22.3)	-	-	-	-
	C_1, conf2	Evolves to C_1, conf1	-	-	-	-
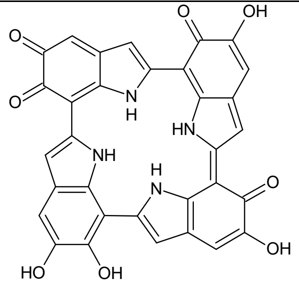 a56_b6_c6_d0	C_1, conf1	−2047.714698 (6.4)	-	-	-	-
	C_1, conf2	Evolves to C_1, conf1	-	-	-	-
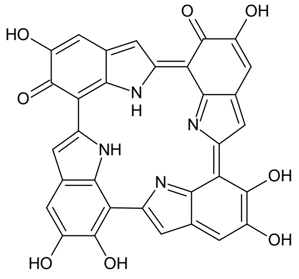 a6_b16_c1_d0	C_1, conf1	−2047.689340 (22.3)	-	-	-	-
	C_1, conf2	Evolves to C_1, conf1	-	-	-	-
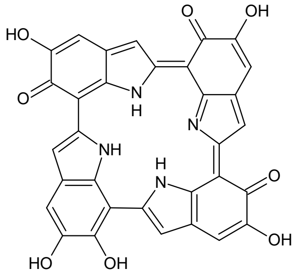 a6_b16_c6_d0	C_1, conf1	**−2047.724901 (0.0)**	**−2047.262199 (0.0)**	**−2047.358235 (0.0)**	−2047.743929 (0.2)	**−2047.377263 (0.0)**
	C_1, conf2	Evolves to C_1, conf1	-	-	-	-

Table S11. *Cont.*

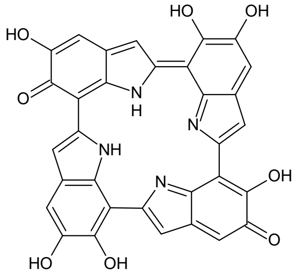 a6_b1_c15_d0	C_1, conf1	−2047.680147 (28.1)	-	-	-	-
	C_1, conf2	Evolves to C_1, conf1	-	-	-	-
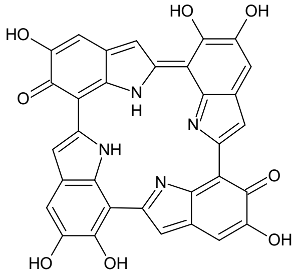 a6_b1_c16_d0	C_1, conf1	−2047.696146 (18.0)	-	-	-	-
	C_1, conf2	Evolves to C_1, conf1	-	-	-	-
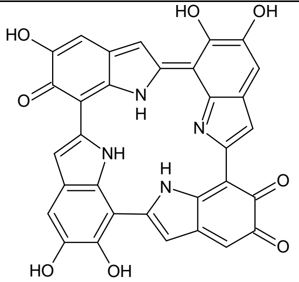 a6_b1_c56_d0	C_1, conf1	−2047.696306 (17.9)	-	-	-	-
	C_1, conf2	Evolves to C_1, conf1	-	-	-	-
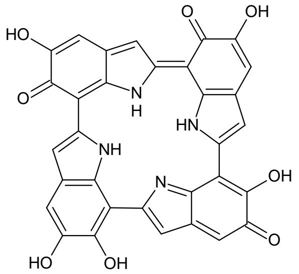 a6_b6_c15_d0	C_1, conf1	−2047.707733 (10.8)	-	-	-	-
	C_1, conf2	Evolves to C_1, conf1	-	-	-	-
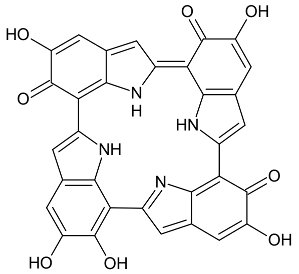 a6_b6_c16_d0	C_1, conf1	−2047.722818 (1.3)	−2047.259858 (1.5)	−2047.355768 (1.5)	−2047.742349 (1.2)	−2047.375299 (1.2)
	C_1, conf2	Evolves to C_1, conf1	-	-	-	-
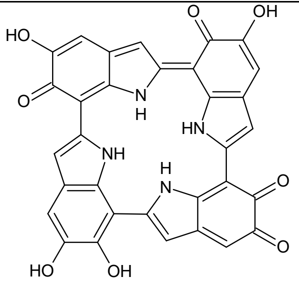 a6_b6_c56_d0	C_1, conf1	−2047.715313 (6.0)	-	-	-	-
	C_1, conf2	Evolves to C_1, conf1	-	-	-	-

Table S11. *Cont.*

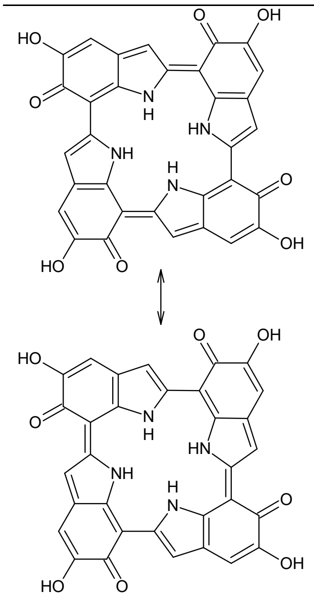 a6_b6_c6_d6	C_1, conf1	−2047.723719 (0.7)	−2047.260186 (1.3)	−2047.354624 (2.3)	**−2047.744321 (0.0)**	−2047.375226 (1.3)
	C_1, conf2	Evolves to C_1, conf1	-	-	-	-
	C_2, conf1	−2047.723546 (0.9)	−2047.260328 (1.2)	−2047.354611 (2.3)	−2047.743800 (0.3)	−2047.374865 (1.5)
	S_4, conf1	Evolves to C_2, conf1	-	-	-	-
	C_4, conf1	Evolves to C_2, conf1	-	-	-	-

In parentheses relative energies (kcal mol^{-1}) refer to the most stable form (in bold) identified at the specified level. [a] For chiral structures, only one enantiomer is listed. [b] Electronic energy including electrostatic contributions at the polarizable continuum model (PCM) level. [c] Enthalpy computed at 298.15 K within the rigid-rotor/harmonic-oscillator (RRHO) approximation. [d] Gibbs free energy computed at 298.15 K within the RRHO approximation. [e] Electronic energy including nonelectrostatic terms according to the SMD solvation model. [f] $G_{SMD,RRHO} = G_{PCM,RRHO} + G_{SMD} - G_{PCM}$.

Table S12. KP-6e, neutral form in vacuo.

Tautomer	Conformer [a]	*E* (Ha) [b]	H_{RRHO} (Ha) [c]	G_{RRHO} (Ha) [d]
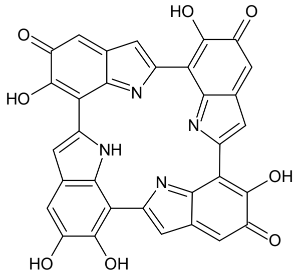 a15_b15_c15_d0	C_1, conf1	−2046.383831 (61.8)	-	-
	C_1, conf2	Evolves to C_1, conf1	-	-
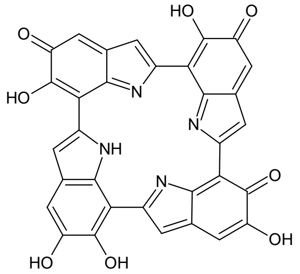 a15_b15_c16_d0	C_1, conf1	−2046.397032 (53.5)	-	-
	C_1, conf2	Evolves to C_1, conf1	-	-
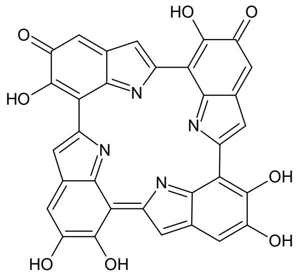 a15_b15_c1_d1	C_1, conf1	−2046.305536 (110.9)	-	-
	C_1, conf2	Evolves to C_1, conf1	-	-
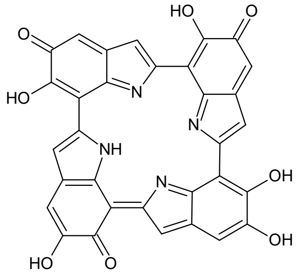 a15_b15_c1_d6	C_1, conf1	−2046.387555 (59.4)	-	-
	C_1, conf2	Evolves to C_1, conf1	-	-
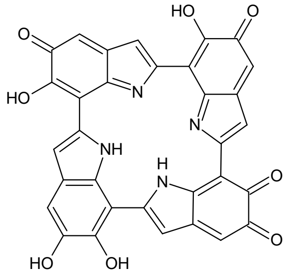 a15_b15_c56_d0	C_1, conf1	−2046.401028 (51.0)	-	-
	C_1, conf2	Evolves to C_1, conf1	-	-

Table S12. *Cont.*

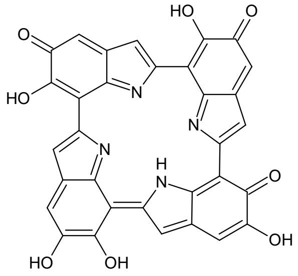	C_1, conf1	−2046.382678 (62.5)	-	-
a15_b15_c6_d1	C_1, conf2	Evolves to C_1, conf1	-	-
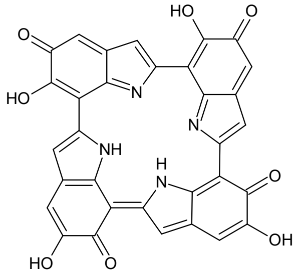	C_1, conf1	−2046.430818 (32.3)	-	-
a15_b15_c6_d6	C_1, conf2	Evolves to C_1, conf1	-	-
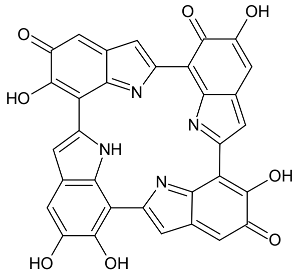	C_1, conf1	−2046.395221 (54.6)	-	-
a15_b16_c15_d0	C_1, conf2	Evolves to C_1, conf1	-	-
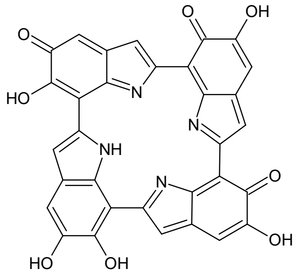	C_1, conf1	−2046.405487 (48.2)	-	-
a15_b16_c16_d0	C_1, conf2	Evolves to C_1, conf1	-	-
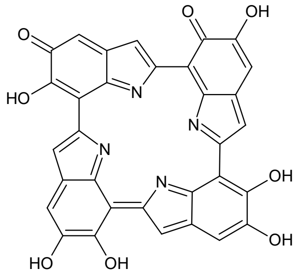	C_1, conf1	−2046.314767 (105.1)	-	-
a15_b16_c1_d1	C_1, conf2	Evolves to C_1, conf1	-	-
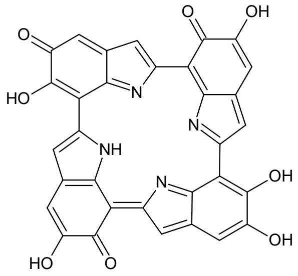	C_1, conf1	−2046.399413 (52.0)	-	-
a15_b16_c1_d6	C_1, conf2	Evolves to C_1, conf1	-	-

Table S12. *Cont.*

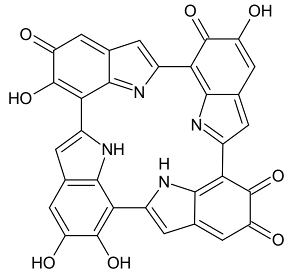 a15_b16_c56_d0	C_1, conf1	−2046.414925 (42.3)	-	-
	C_1, conf2	Evolves to C_1, conf1	-	-
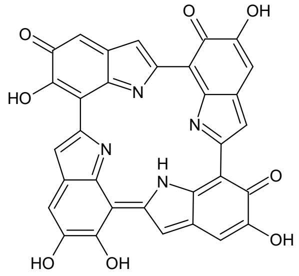 a15_b16_c6_d1	C_1, conf1	−2046.394871 (54.9)	-	-
	C_1, conf2	Evolves to C_1, conf1	-	-
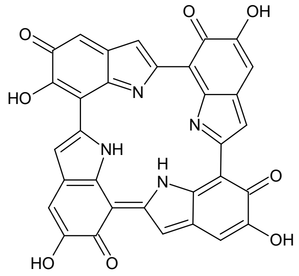 a15_b16_c6_d6	C_1, conf1	−2046.441008 (25.9)	-	-
	C_1, conf2	Evolves to C_1, conf1	-	-
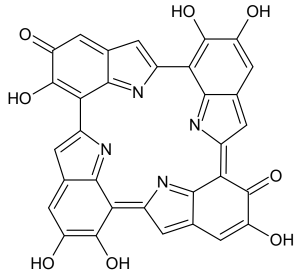 a15_b1_c16_d1	C_1, conf1	−2046.313834 (105.7)	-	-
	C_1, conf2	Evolves to C_1, conf1	-	-
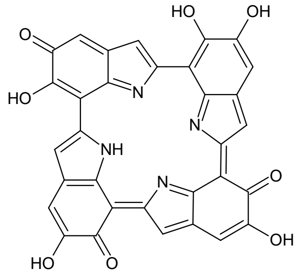 a15_b1_c16_d6	C_1, conf1	−2046.395274 (54.6)	-	-
	C_1, conf2	Evolves to C_1, conf1	-	-
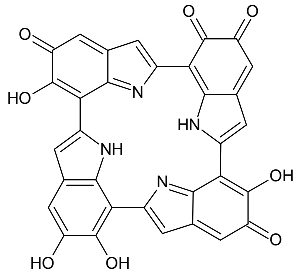 a15_b56_c15_d0	C_1, conf1	−2046.410049 (45.3)	-	-
	C_1, conf2	Evolves to C_1, conf1	-	-

Table S12. *Cont.*

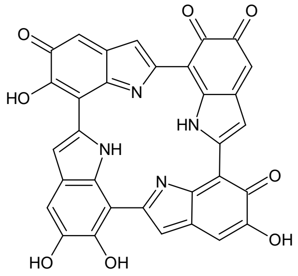 a15_b56_c16_d0	C_1, conf1	−2046.424657 (36.2)	-	-
	C_1, conf2	Evolves to C_1, conf1	-	-
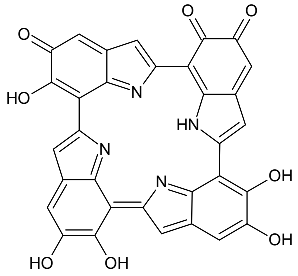 a15_b56_c1_d1	C_1, conf1	−2046.347254 (84.7)	-	-
	C_1, conf2	Evolves to C_1, conf1	-	-
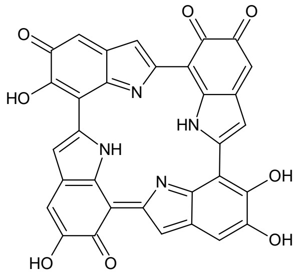 a15_b56_c1_d6	C_1, conf1	−2046.419064 (39.7)	-	-
	C_1, conf2	Evolves to C_1, conf1	-	-
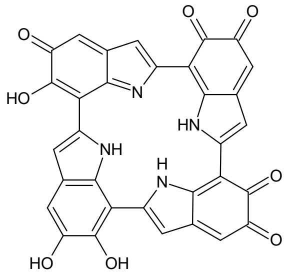 a15_b56_c56_d0	C_1, conf1	−2046.407369 (47.0)	-	-
	C_1, conf2	Evolves to C_1, conf1	-	-
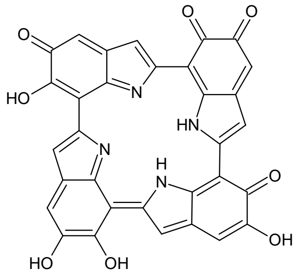 a15_b56_c6_d1	C_1, conf1	−2046.399438 (52.0)	-	-
	C_1, conf2	Evolves to C_1, conf1	-	-
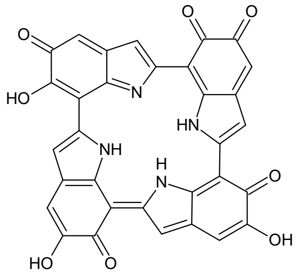 a15_b56_c6_d6	C_1, conf1	−2046.432108 (31.5)	-	-
	C_1, conf2	Evolves to C_1, conf1	-	-

Table S12. *Cont.*

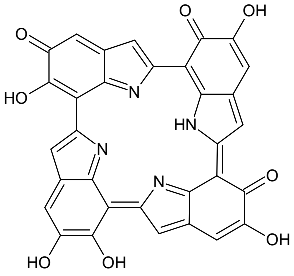 a15_b6_c16_d1	C_1, conf1	−2046.394358 (55.2)	-	-
	C_1, conf2	Evolves to C_1, conf1	-	-
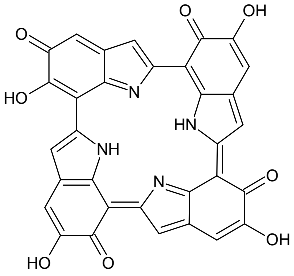 a15_b6_c16_d6	C_1, conf1	−2046.454423 (17.5)	-	-
	C_1, conf2	Evolves to a different tautomer	-	-
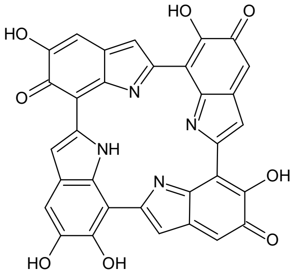 a16_b15_c15_d0	C_1, conf1	−2046.396587 (53.8)	-	-
	C_1, conf2	Evolves to C_1, conf1	-	-
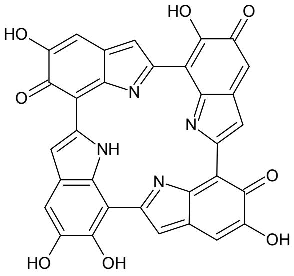 a16_b15_c16_d0	C_1, conf1	−2046.408755 (46.1)	-	-
	C_1, conf2	Evolves to C_1, conf1	-	-
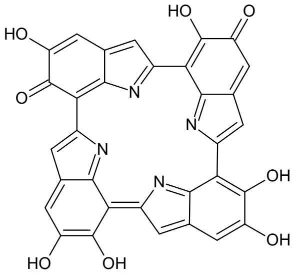 a16_b15_c1_d1	C_1, conf1	−2046.314789 (105.1)	-	-
	C_1, conf2	Evolves to C_1, conf1	-	-
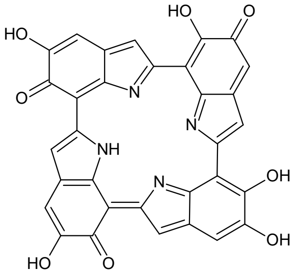 a16_b15_c1_d6	C_1, conf1	−2046.398742 (52.4)	-	-
	C_1, conf2	Evolves to C_1, conf1	-	-

Table S12. *Cont.*

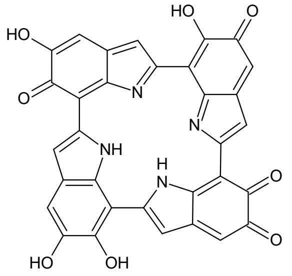 a16_b15_c56_d0	C_1, conf1	−2046.415824 (41.7)	-	-
	C_1, conf2	Evolves to C_1, conf1	-	-
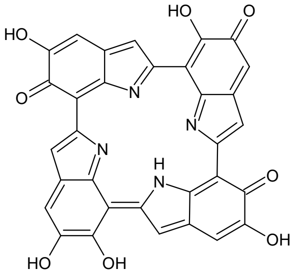 a16_b15_c6_d1	C_1, conf1	−2046.395262 (54.6)	-	-
	C_1, conf2	Evolves to C_1, conf1	-	-
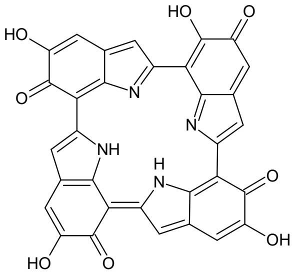 a16_b15_c6_d6	C_1, conf1	−2046.442111 (25.2)	-	-
	C_1, conf2	Evolves to C_1, conf1	-	-
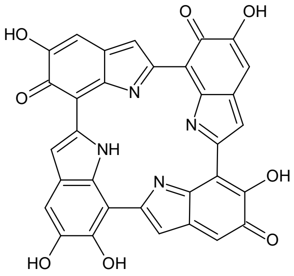 a16_b16_c15_d0	C_1, conf1	−2046.404817 (48.6)	-	-
	C_1, conf2	Evolves to C_1, conf1	-	-
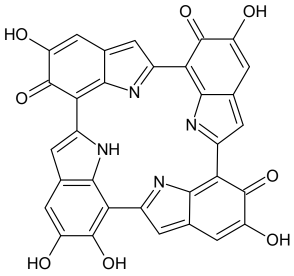 a16_b16_c16_d0	C_1, conf1	−2046.416449 (41.3)	-	-
	C_1, conf2	Evolves to C_1, conf1	-	-

Table S12. *Cont.*

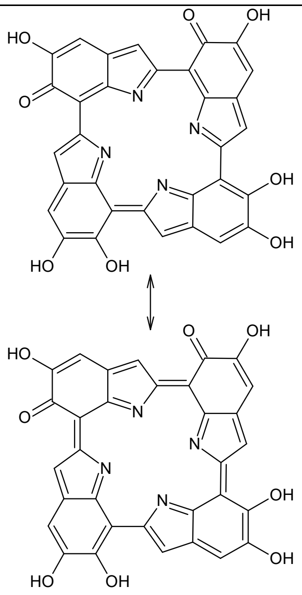	C_1, conf1	−2046.322541 (100.2)	-	-
a16_b16_c1_d1	C_1, conf2	−2046.322017 (100.6)	-	-
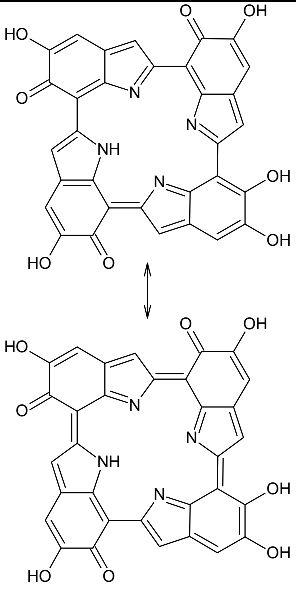	C_1, conf1	−2046.409868 (45.4)	-	-
a16_b16_c1_d6	C_1, conf2	Evolves to C_1, conf1	-	-
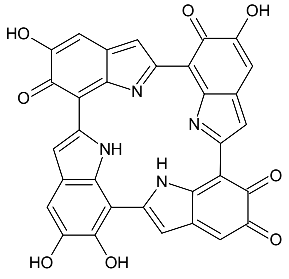	C_1, conf1	−2046.428450 (33.8)	-	-
a16_b16_c56_d0	C_1, conf2	Evolves to C_1, conf1	-	-

Table S12. *Cont.*

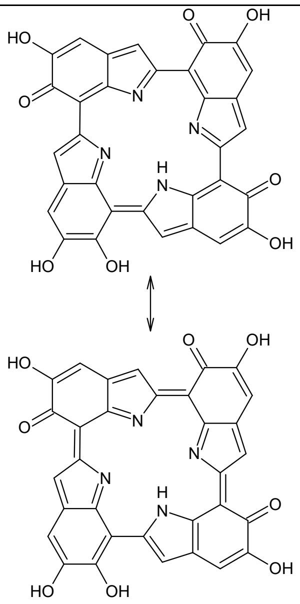	C_1, conf1	−2046.405990 (47.9)	-	-
a16_b16_c6_d1	C_1, conf2	Evolves to C_1, conf1	-	-
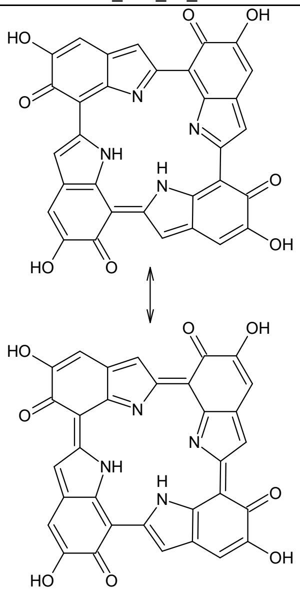	C_1, conf1	−2046.467842 (9.1)	-	-
a16_b16_c6_d6	C_1, conf2	Evolves to C_1, conf1	-	-

Table S12. *Cont.*

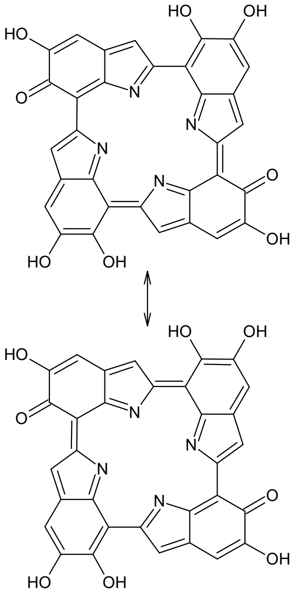 a16_b1_c16_d1	C_1, conf1	−2046.323564 (99.6)	-	-
	C_1, conf2	Evolves to C_1, conf1	-	-
	C_2, conf1	−2046.311204 (107.4)	-	-
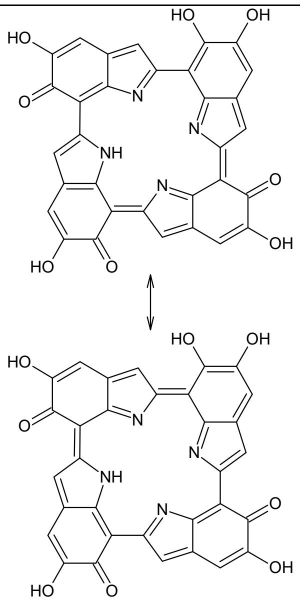 a16_b1_c16_d6	C_1, conf1	−2046.408208 (46.5)	-	-
	C_1, conf2	Evolves to C_1, conf1	-	-
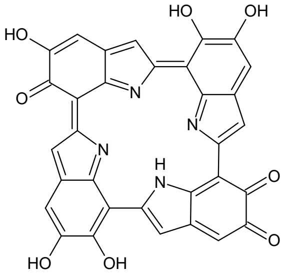 a16_b1_c56_d1	C_1, conf1	−2046.352410 (81.5)	-	-
	C_1, conf2	Evolves to C_1, conf1	-	-

Table S12. *Cont.*

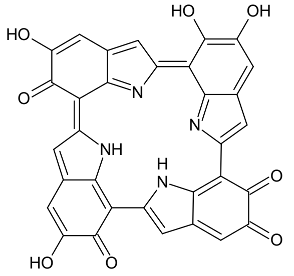 a16_b1_c56_d6	C_1, conf1	−2046.407292 (47.1)	-	-
	C_1, conf2	Evolves to C_1, conf1	-	-
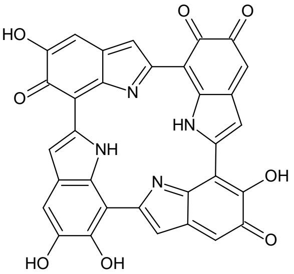 a16_b56_c15_d0	C_1, conf1	−2046.425910 (35.4)	-	-
	C_1, conf2	Evolves to C_1, conf1	-	-
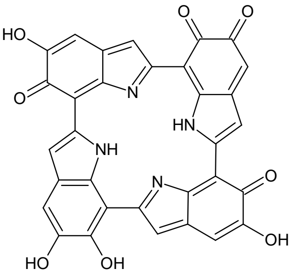 a16_b56_c16_d0	C_1, conf1	−2046.441030 (25.9)	-	-
	C_1, conf2	Evolves to C_1, conf1	-	-
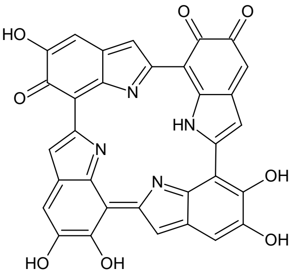 a16_b56_c1_d1	C_1, conf1	−2046.360616 (76.3)	-	-
	C_1, conf2	Evolves to C_1, conf1	-	-
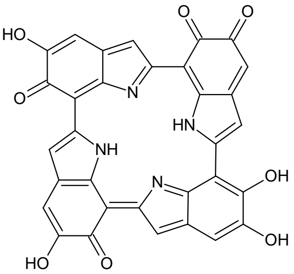 a16_b56_c1_d6	C_1, conf1	−2046.435845 (29.1)	-	-
	C_1, conf2	Evolves to C_1, conf1	-	-
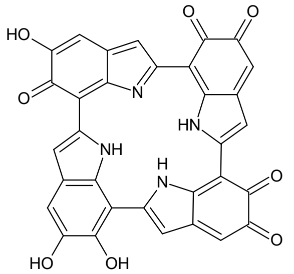 a16_b56_c56_d0	C_1, conf1	−2046.423378 (37.0)	-	-
	C_1, conf2	Evolves to C_1, conf1	-	-

Table S12. *Cont.*

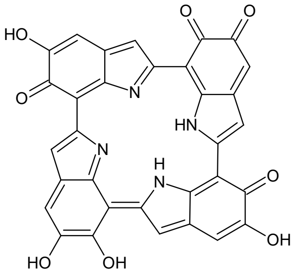 a16_b56_c6_d1	C_1, conf1	−2046.414364 (42.6)	-	-
	C_1, conf2	Evolves to C_1, conf1	-	-
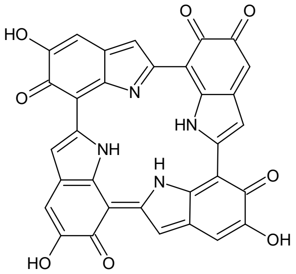 a16_b56_c6_d6	C_1, conf1	−2046.450064 (20.2)	-	-
	C_1, conf2	Evolves to C_1, conf1	-	-
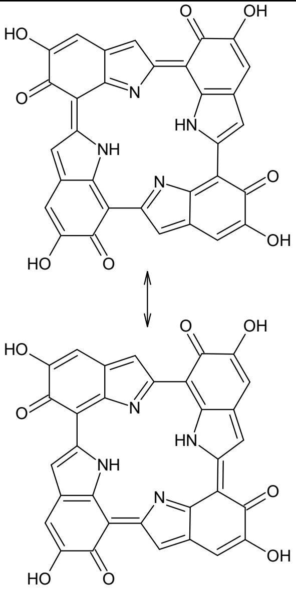 a16_b6_c16_d6	C_1, conf1	−2046.482284 (0.0)	-	-
	C_1, conf2	Evolves to C_1, conf1	-	-
	C_2, conf1	**−2046.482242 (0.0)**	**−2046.043017 (0.0)**	**−2046.138676 (0.0)**
	C_{2h}, conf1	−2046.482289 (0.0)	First-order saddle point	-
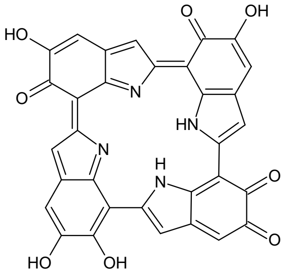 a16_b6_c56_d1	C_1, conf1	−2046.418406 (40.1)	-	-
	C_1, conf2	Evolves to C_1, conf1	-	-
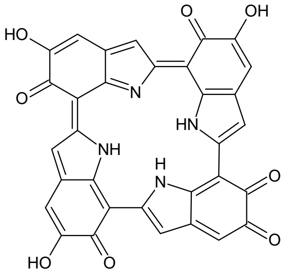 a16_b6_c56_d6	C_1, conf1	−2046.449827 (20.4)	-	-
	C_1, conf2	Evolves to C_1, conf1	-	-

Table S12. *Cont.*

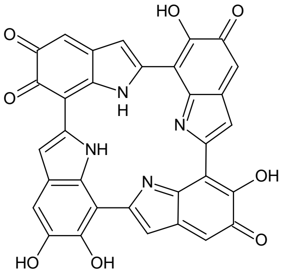 a56_b15_c15_d0	C_1, conf1	−2046.395156 (54.7)	-	-
	C_1, conf2	Evolves to C_1, conf1	-	-
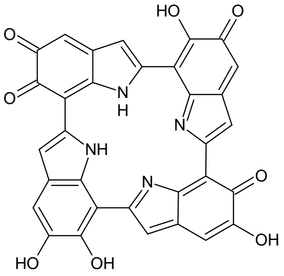 a56_b15_c16_d0	C_1, conf1	−2046.408816 (46.1)	-	-
	C_1, conf2	Evolves to C_1, conf1	-	-
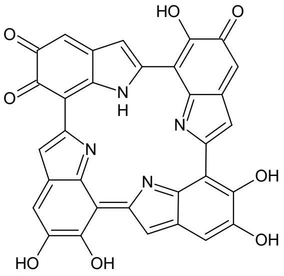 a56_b15_c1_d1	C_1, conf1	−2046.343856 (86.9)	-	-
	C_1, conf2	Evolves to C_1, conf1	-	-
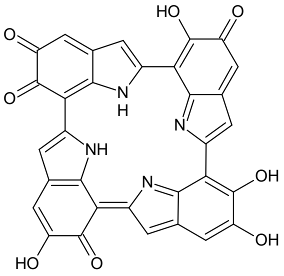 a56_b15_c1_d6	C_1, conf1	−2046.403724 (49.3)	-	-
	C_1, conf2	Evolves to C_1, conf1	-	-
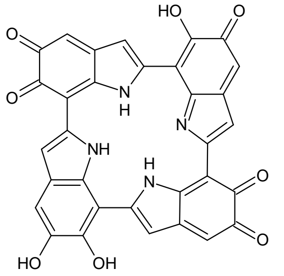 a56_b15_c56_d0	C_1, conf1	−2046.406002 (47.9)	-	-
	C_1, conf2	Evolves to C_1, conf1	-	-
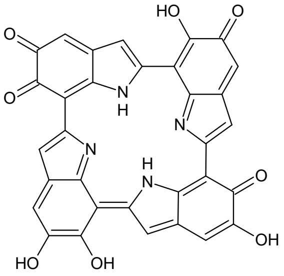 a56_b15_c6_d1	C_1, conf1	−2046.406481 (47.6)	-	-
	C_1, conf2	Evolves to C_1, conf1	-	-

Table S12. *Cont.*

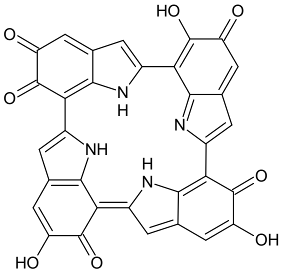<br>a56_b15_c6_d6	C_1, conf1	−2046.431965 (31.6)	-	-
	C_1, conf2	Evolves to C_1, conf1	-	-
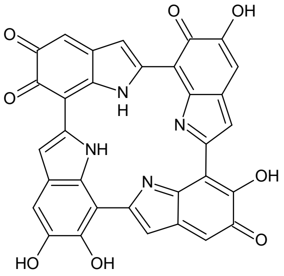<br>a56_b16_c15_d0	C_1, conf1	−2046.408448 (46.3)	-	-
	C_1, conf2	Evolves to C_1, conf1	-	-
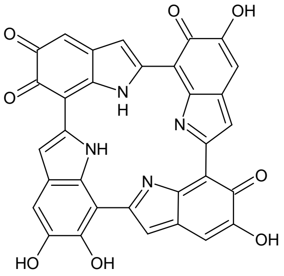<br>a56_b16_c16_d0	C_1, conf1	−2046.420495 (38.8)	-	-
	C_1, conf2	Evolves to C_1, conf1	-	-
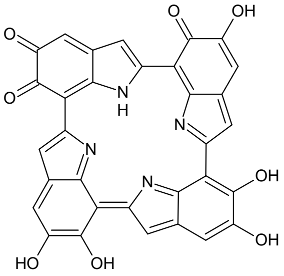<br>a56_b16_c1_d1	C_1, conf1	−2046.355012 (79.9)	-	-
	C_1, conf2	Evolves to C_1, conf1	-	-
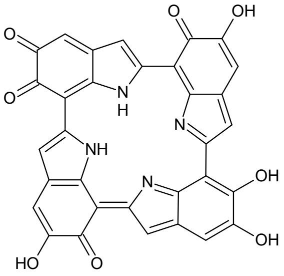<br>a56_b16_c1_d6	C_1, conf1	−2046.420301 (38.9)	-	-
	C_1, conf2	Evolves to C_1, conf1	-	-
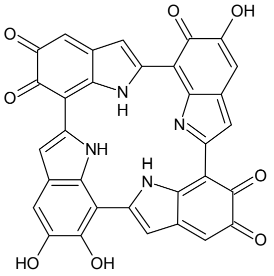<br>a56_b16_c56_d0	C_1, conf1	−2046.420420 (38.8)	-	-
	C_1, conf2	Evolves to C_1, conf1	-	-

Table S12. *Cont.*

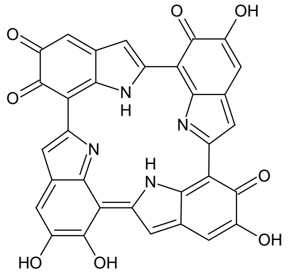 a56_b16_c6_d1	C_1, conf1	−2046.420833 (38.6)	-	-
	C_1, conf2	Evolves to C_1, conf1	-	-
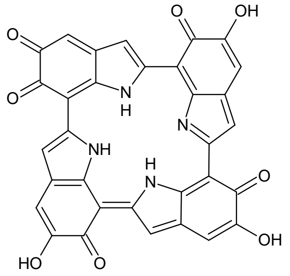 a56_b16_c6_d6	C_1, conf1	−2046.448290 (21.3)	-	-
	C_1, conf2	Evolves to C_1, conf1	-	-
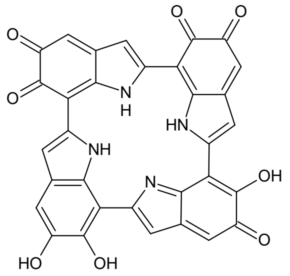 a56_b56_c15_d0	C_1, conf1	−2046.403634 (49.4)	-	-
	C_1, conf2	Evolves to C_1, conf1	-	-
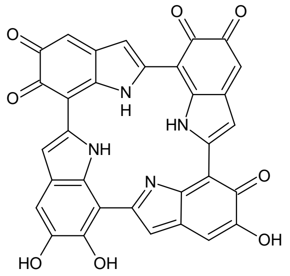 a56_b56_c16_d0	C_1, conf1	−2046.418074 (40.3)	-	-
	C_1, conf2	Evolves to C_1, conf1	-	-
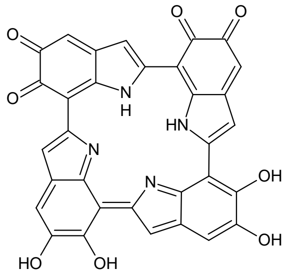 a56_b56_c1_d1	C_1, conf1	−2046.363000 (74.9)	-	-
	C_1, conf2	Evolves to C_1, conf1	-	-
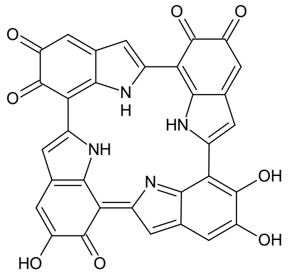 a56_b56_c1_d6	C_1, conf1	−2046.412435 (43.8)	-	-
	C_1, conf2	Evolves to C_1, conf1	-	-

Table S12. *Cont.*

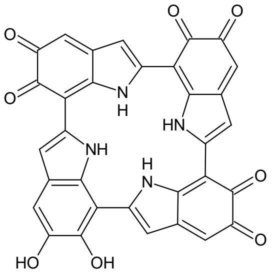 a56_b56_c56_d0	C_1, conf1	−2046.401527 (50.7)	-	-
	C_1, conf2	Evolves to C_1, conf1	-	-
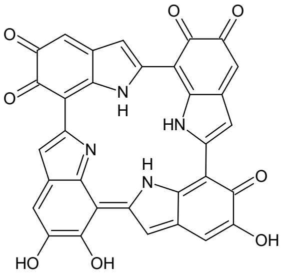 a56_b56_c6_d1	C_1, conf1	−2046.401592 (50.6)	-	-
	C_1, conf2	Evolves to C_1, conf1	-	-
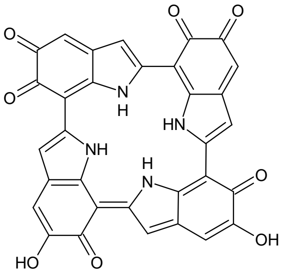 a56_b56_c6_d6	C_1, conf1	−2046.424365 (36.3)	-	-
	C_1, conf2	Evolves to C_1, conf1	-	-

In parentheses relative energies (kcal mol^{-1}) refer to the most stable form (in bold) identified at the specified level. [a] For chiral structures, only one enantiomer is listed. [b] Electronic energy. [c] Enthalpy computed at 298.15 K within the rigid-rotor/harmonic-oscillator (RRHO) approximation. [d] Gibbs free energy computed at 298.15 K within the RRHO approximation.

Table S13. KP-6e, neutral form in water.

Tautomer	Conformer [a]	G_{PCM} (Ha) [b]	$H_{\mathrm{PCM,RRHO}}$ (Ha) [c]	$G_{\mathrm{PCM,RRHO}}$ (Ha) [d]	G_{SMD} (Ha) [e]	$G_{\mathrm{SMD,RRHO}}$ (Ha) [f]
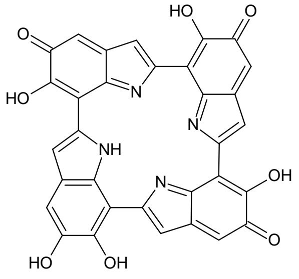 a15_b15_c15_d0	C_1, conf1	−2046.409084 (55.5)	-	-	-	-
	C_1, conf2	Evolves to C_1, conf1	-	-	-	-
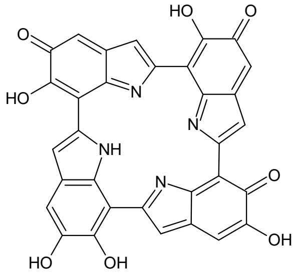 a15_b15_c16_d0	C_1, conf1	−2046.422437 (47.1)	-	-	-	-
	C_1, conf2	Evolves to C_1, conf1	-	-	-	-
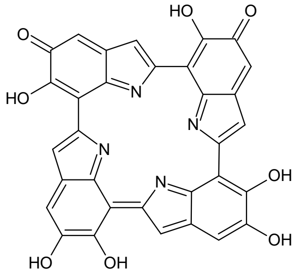 a15_b15_c1_d1	C_1, conf1	−2046.353436 (90.4)	-	-	-	-
	C_1, conf2	Evolves to C_1, conf1	-	-	-	-
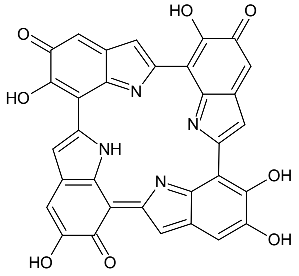 a15_b15_c1_d6	C_1, conf1	−2046.414522 (52.1)	-	-	-	-
	C_1, conf2	Evolves to C_1, conf1	-	-	-	-
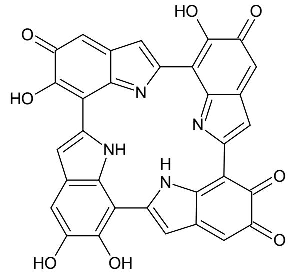 a15_b15_c56_d0	C_1, conf1	−2046.429797 (42.5)	-	-	-	-
	C_1, conf2	Evolves to C_1, conf1	-	-	-	-

Table S13. *Cont.*

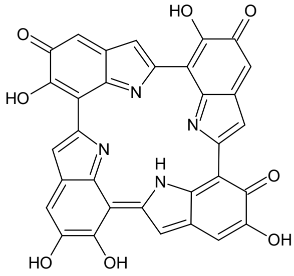 a15_b15_c6_d1	C_1, conf1	−2046.409686 (55.1)	-	-	-	-
	C_1, conf2	Evolves to C_1, conf1	-	-	-	-
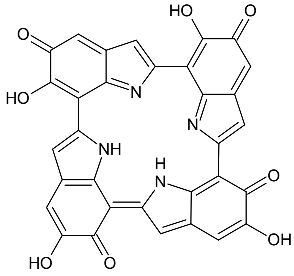 a15_b15_c6_d6	C_1, conf1	−2046.449454 (30.1)	-	-	-	-
	C_1, conf2	Evolves to C_1, conf1	-	-	-	-
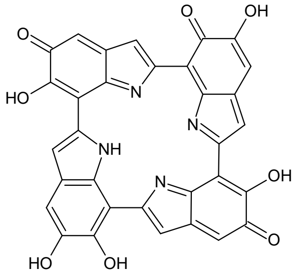 a15_b16_c15_d0	C_1, conf1	−2046.420481 (48.3)	-	-	-	-
	C_1, conf2	Evolves to C_1, conf1	-	-	-	-
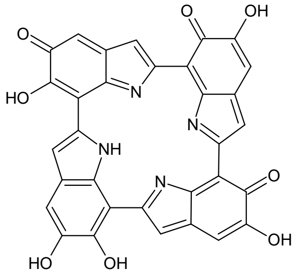 a15_b16_c16_d0	C_1, conf1	−2046.430696 (41.9)	-	-	-	-
	C_1, conf2	Evolves to C_1, conf1	-	-	-	-
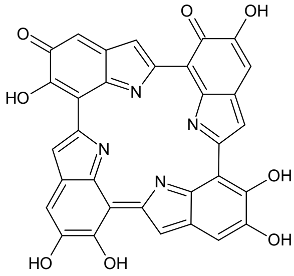 a15_b16_c1_d1	C_1, conf1	−2046.363606 (84.0)	-	-	-	-
	C_1, conf2	Evolves to C_1, conf1	-	-	-	-
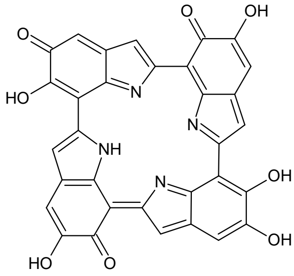 a15_b16_c1_d6	C_1, conf1	−2046.426215 (44.7)	-	-	-	-
	C_1, conf2	Evolves to C_1, conf1	-	-	-	-

Table S13. *Cont.*

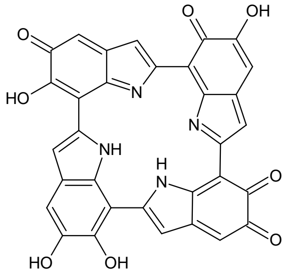 a15_b16_c56_d0	C_1, conf1	−2046.442512 (34.5)	-	-	-	-
	C_1, conf2	Evolves to C_1, conf1	-	-	-	-
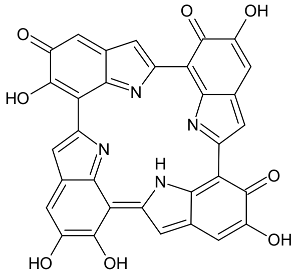 a15_b16_c6_d1	C_1, conf1	−2046.421542 (47.7)	-	-	-	-
	C_1, conf2	Evolves to C_1, conf1	-	-	-	-
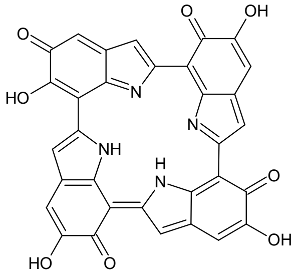 a15_b16_c6_d6	C_1, conf1	−2046.459088 (24.1)	-	-	-	-
	C_1, conf2	Evolves to C_1, conf1	-	-	-	-
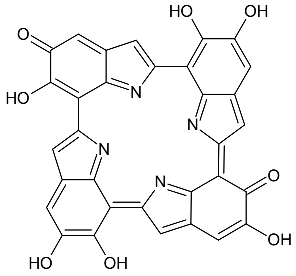 a15_b1_c16_d1	C_1, conf1	−2046.361620 (85.3)	-	-	-	-
	C_1, conf2	Evolves to C_1, conf1	-	-	-	-
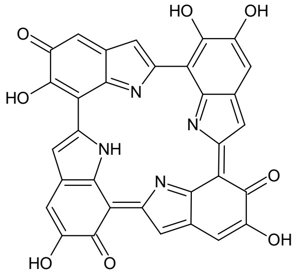 a15_b1_c16_d6	C_1, conf1	−2046.422444 (47.1)	-	-	-	-
	C_1, conf2	Evolves to C_1, conf1	-	-	-	-
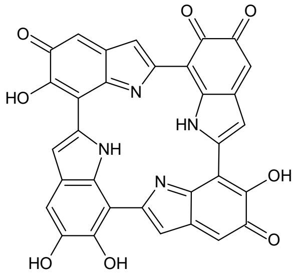 a15_b56_c15_d0	C_1, conf1	−2046.438349 (37.1)	-	-	-	-
	C_1, conf2	Evolves to C_1, conf1	-	-	-	-

Table S13. *Cont.*

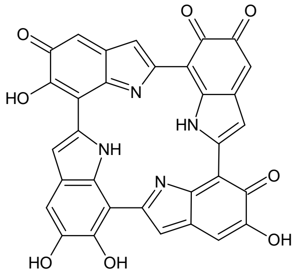 a15_b56_c16_d0	C_1, conf1	−2046.452009 (28.5)	-	-	-	-
	C_1, conf2	Evolves to C_1, conf1	-	-	-	-
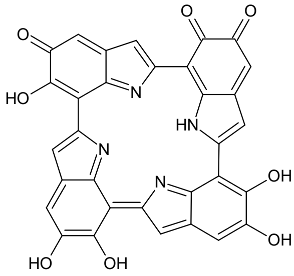 a15_b56_c1_d1	C_1, conf1	−2046.389381 (67.8)	-	-	-	-
	C_1, conf2	Evolves to C_1, conf1	-	-	-	-
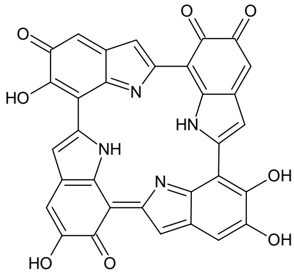 a15_b56_c1_d6	C_1, conf1	−2046.447425 (31.4)	-	-	-	-
	C_1, conf2	Evolves to C_1, conf1	-	-	-	-
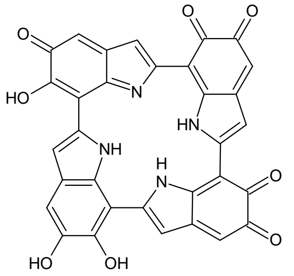 a15_b56_c56_d0	C_1, conf1	−2046.445640 (32.5)	-	-	-	-
	C_1, conf2	Evolves to C_1, conf1	-	-	-	-
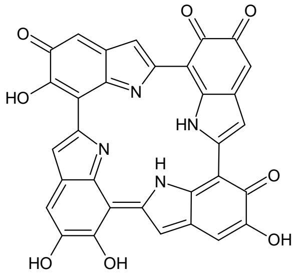 a15_b56_c6_d1	C_1, conf1	−2046.430162 (42.2)	-	-	-	-
	C_1, conf2	Evolves to C_1, conf1	-	-	-	-
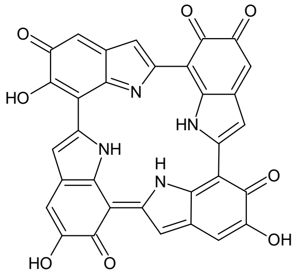 a15_b56_c6_d6	C_1, conf1	−2046.460860 (23.0)	-	-	-	-
	C_1, conf2	Evolves to C_1, conf1	-	-	-	-

Table S13. *Cont.*

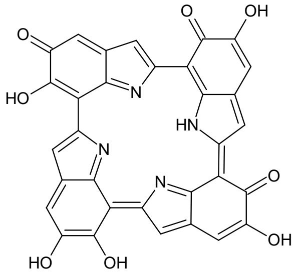<br>a15_b6_c16_d1	C_1, conf1	−2046.420376 (48.4)	-	-	-	-
	C_1, conf2	Evolves to C_1, conf1	-	-	-	-
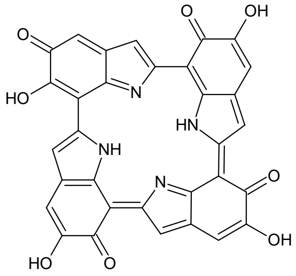<br>a15_b6_c16_d6	C_1, conf1	−2046.471338 (16.4)	-	-	-	-
	C_1, conf2	Evolves to a different tautomer	-	-	-	-
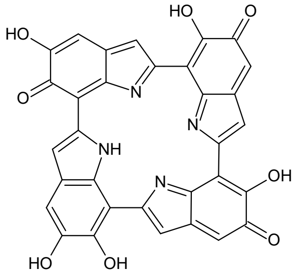<br>a16_b15_c15_d0	C_1, conf1	−2046.421880 (47.4)	-	-	-	-
	C_1, conf2	Evolves to C_1, conf1	-	-	-	-
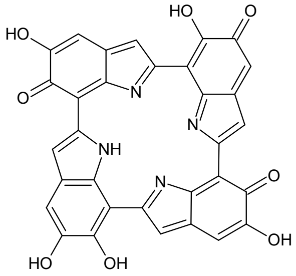<br>a16_b15_c16_d0	C_1, conf1	−2046.434039 (39.8)	-	-	-	-
	C_1, conf2	Evolves to C_1, conf1	-	-	-	-
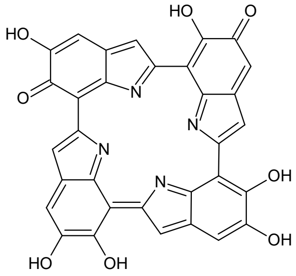<br>a16_b15_c1_d1	C_1, conf1	−2046.363974 (83.8)	-	-	-	-
	C_1, conf2	Evolves to C_1, conf1	-	-	-	-
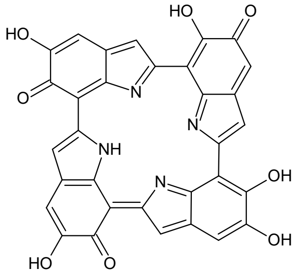<br>a16_b15_c1_d6	C_1, conf1	−2046.425252 (45.3)	-	-	-	-
	C_1, conf2	Evolves to C_1, conf1	-	-	-	-

Table S13. *Cont.*

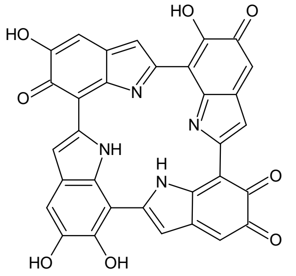 a16_b15_c56_d0	C_1, conf1	−2046.444032 (33.5)	-	-	-	-
	C_1, conf2	Evolves to C_1, conf1	-	-	-	-
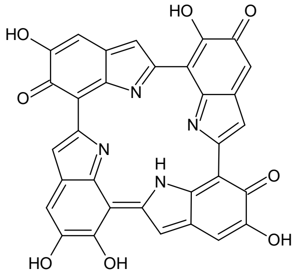 a16_b15_c6_d1	C_1, conf1	−2046.422734 (46.9)	-	-	-	-
	C_1, conf2	Evolves to C_1, conf1	-	-	-	-
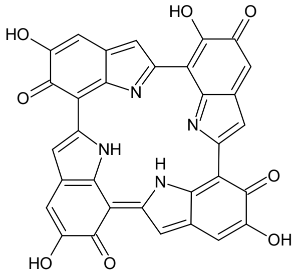 a16_b15_c6_d6	C_1, conf1	Evolves to a different tautomer	-	-	-	-
	C_1, conf2	−2046.460291 (23.3)	-	-	-	-
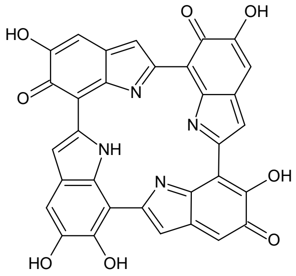 a16_b16_c15_d0	C_1, conf1	−2046.430223 (42.2)	-	-	-	-
	C_1, conf2	Evolves to C_1, conf1	-	-	-	-
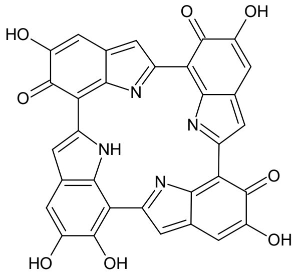 a16_b16_c16_d0	C_1, conf1	−2046.443222 (34.0)	-	-	-	-
	C_1, conf2	Evolves to C_1, conf1	-	-	-	-

Table S13. *Cont.*

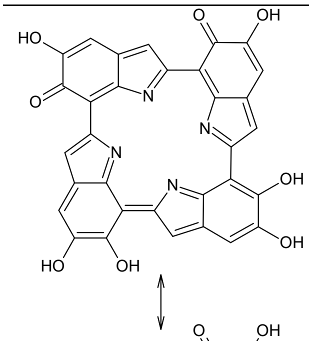	C_1, conf1	−2046.368903 (80.7)	-	-	-	-
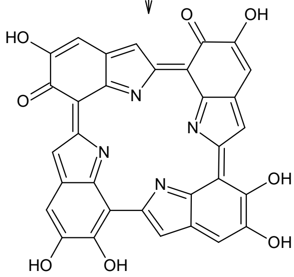 a16_b16_c1_d1	C_1, conf2	−2046.372479 (78.5)	-	-	-	-
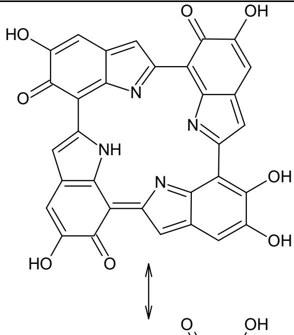	C_1, conf1	−2046.438753 (36.9)	-	-	-	-
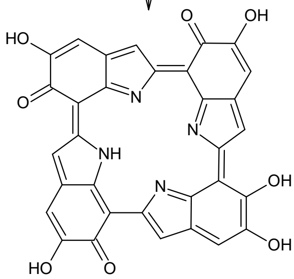 a16_b16_c1_d6	C_1, conf2	Evolves to C_1, conf1	-	-	-	-
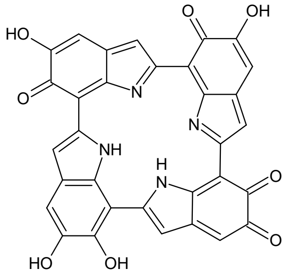	C_1, conf1	−2046.456176 (25.9)	-	-	-	-
a16_b16_c56_d0	C_1, conf2	Evolves to C_1, conf1	-	-	-	-

Table S13. *Cont.*

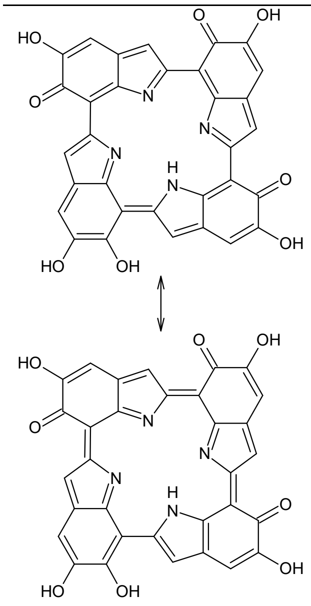	C_1, conf1	−2046.435815 (38.7)	-	-	-	-
a16_b16_c6_d1	C_1, conf2	Evolves to C_1, conf1	-	-	-	-
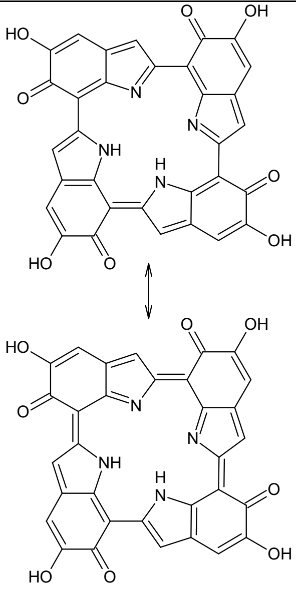	C_1, conf1	−2046.484846 (7.9)	-	-	-	-
a16_b16_c6_d6	C_1, conf2	Evolves to C_1, conf1	-	-	-	-

Table S13. *Cont.*

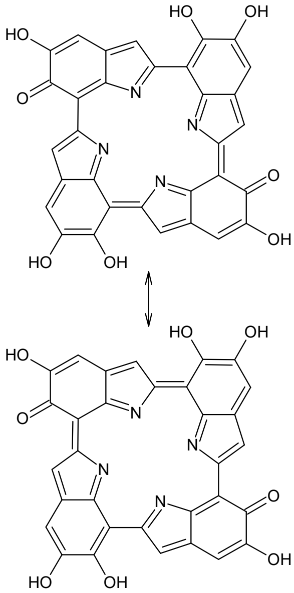 a16_b1_c16_d1	C_1, conf1	−2046.372748 (78.3)	-	-	-	-
	C_1, conf2	−2046.367494 (81.6)	-	-	-	-
	C_2, conf1	−2046.366678 (82.1)	-	-	-	-
	C_2, conf2	Evolves to C_2, conf1	-	-	-	-
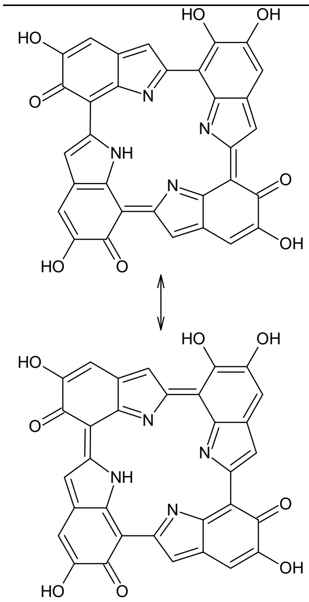 a16_b1_c16_d6	C_1, conf1	−2046.437714 (37.5)	-	-	-	-
	C_1, conf2	Evolves to C_1, conf1	-	-	-	-
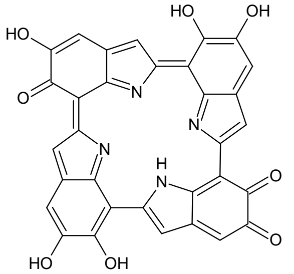 a16_b1_c56_d1	C_1, conf1	−2046.397239 (62.9)	-	-	-	-
	C_1, conf2	−2046.396896 (63.1)	-	-	-	-

Table S13. *Cont.*

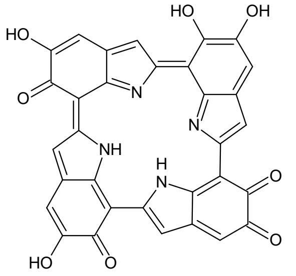 a16_b1_c56_d6	C_1, conf1	−2046.438676 (36.9)	-	-	-	-
	C_1, conf2	Evolves to C_1, conf1	-	-	-	-
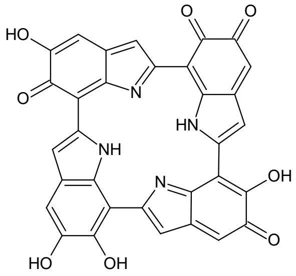 a16_b56_c15_d0	C_1, conf1	−2046.453980 (27.3)	-	-	-	-
	C_1, conf2	Evolves to C_1, conf1	-	-	-	-
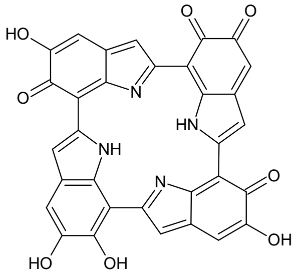 a16_b56_c16_d0	C_1, conf1	−2046.468443 (18.2)	-	-	-	-
	C_1, conf2	Evolves to C_1, conf1	-	-	-	-
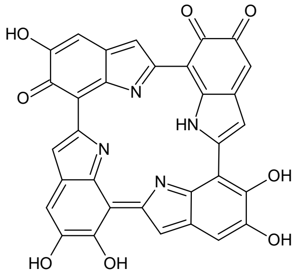 a16_b56_c1_d1	C_1, conf1	−2046.403482 (59.0)	-	-	-	-
	C_1, conf2	Evolves to C_1, conf1	-	-	-	-
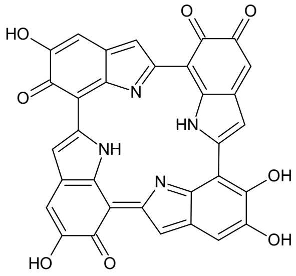 a16_b56_c1_d6	C_1, conf1	−2046.463497 (21.3)	-	-	-	-
	C_1, conf2	Evolves to C_1, conf1	-	-	-	-
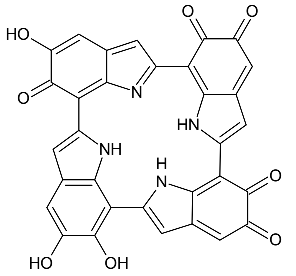 a16_b56_c56_d0	C_1, conf1	−2046.460727 (23.1)	-	-	-	-
	C_1, conf2	Evolves to C_1, conf1	-	-	-	-

Table S13. *Cont.*

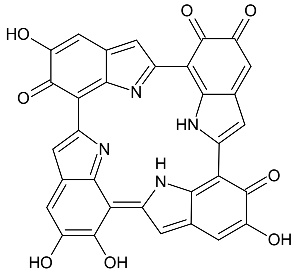 a16_b56_c6_d1	C_1, conf1	−2046.445751 (32.5)	-	-	-	-
	C_1, conf2	Evolves to C_1, conf1	-	-	-	-
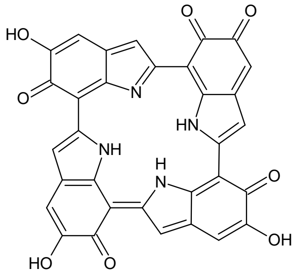 a16_b56_c6_d6	C_1, conf1	−2046.478301 (12.0)	-	-	-	-
	C_1, conf2	Evolves to C_1, conf1	-	-	-	-
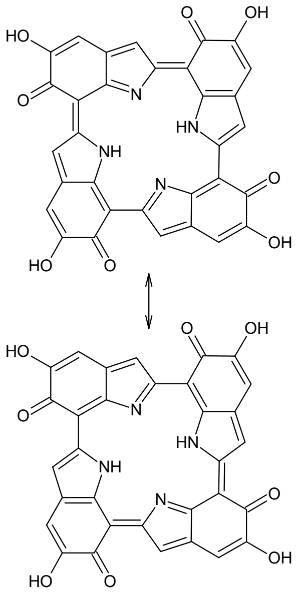 a16_b6_c16_d6	C_1, conf1	−2046.497644 (−0.1)	-	-	-	-
	C_1, conf2	Evolves to C_1, conf1	-	-	-	-
	C_2, conf1	**−2046.497479 (0.0)**	**−2046.059837 (0.0)**	**−2046.154721 (0.0)**	**−2046.509330 (0.0)**	**−2046.166572 (0.0)**
	C_{2h}, conf1	−2046.497228 (0.2)	First-order saddle point	-	−2046.508966 (0.2)	-
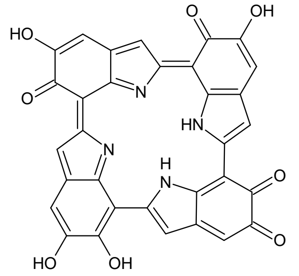 a16_b6_c56_d1	C_1, conf1	−2046.448259 (30.9)	-	-	-	-
	C_1, conf2	Evolves to C_1, conf1	-	-	-	-

Table S13. *Cont.*

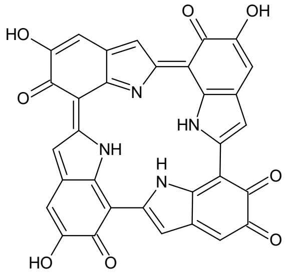 a16_b6_c56_d6	C_1, conf1	−2046.478344 (12.0)	-	-	-	-
	C_1, conf2	Evolves to C_1, conf1	-	-	-	-
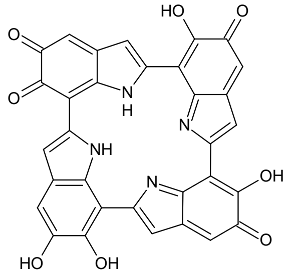 a56_b15_c15_d0	C_1, conf1	−2046.426691 (44.4)	-	-	-	-
	C_1, conf2	Evolves to C_1, conf1	-	-	-	-
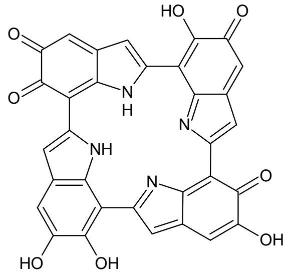 a56_b15_c16_d0	C_1, conf1	−2046.439270 (36.5)	-	-	-	-
	C_1, conf2	Evolves to C_1, conf1	-	-	-	-
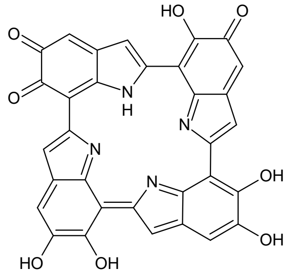 a56_b15_c1_d1	C_1, conf1	−2046.386997 (69.3)	-	-	-	-
	C_1, conf2	Evolves to C_1, conf1	-	-	-	-
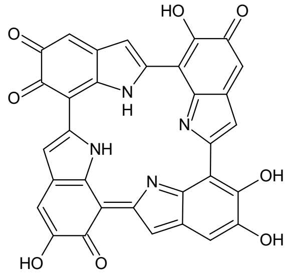 a56_b15_c1_d6	C_1, conf1	−2046.433218 (40.3)	-	-	-	-
	C_1, conf2	Evolves to C_1, conf1	-	-	-	-
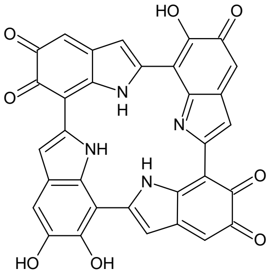 a56_b15_c56_d0	C_1, conf1	−2046.445021 (32.9)	-	-	-	-
	C_1, conf2	Evolves to C_1, conf1	-	-	-	-

Table S13. *Cont.*

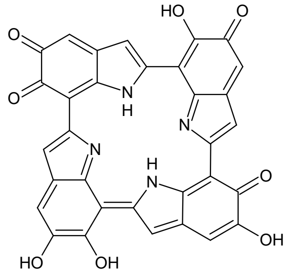 a56_b15_c6_d1	C_1, conf1	−2046.438754 (36.8)	-	-	-	-
	C_1, conf2	Evolves to C_1, conf1	-	-	-	-
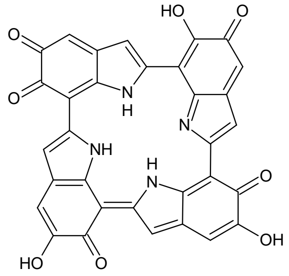 a56_b15_c6_d6	C_1, conf1	−2046.460148 (23.4)	-	-	-	-
	C_1, conf2	Evolves to C_1, conf1	-	-	-	-
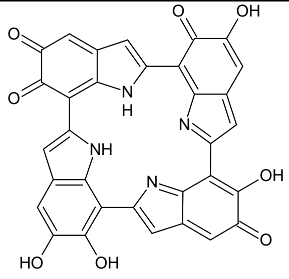 a56_b16_c15_d0	C_1, conf1	−2046.438384 (37.1)	-	-	-	-
	C_1, conf2	Evolves to C_1, conf1	-	-	-	-
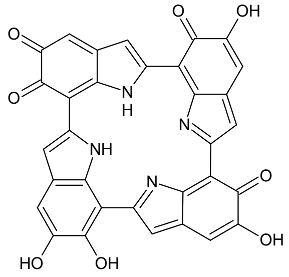 a56_b16_c16_d0	C_1, conf1	−2046.450216 (29.7)	-	-	-	-
	C_1, conf2	Evolves to C_1, conf1	-	-	-	-
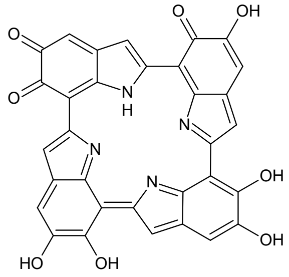 a56_b16_c1_d1	C_1, conf1	−2046.398138 (62.3)	-	-	-	-
	C_1, conf2	Evolves to C_1, conf1	-	-	-	-
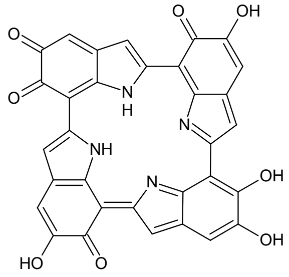 a56_b16_c1_d6	C_1, conf1	−2046.449384 (30.2)	-	-	-	-
	C_1, conf2	Evolves to C_1, conf1	-	-	-	-

Table S13. *Cont.*

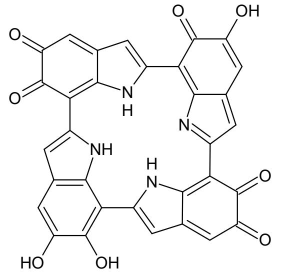 a56_b16_c56_d0	C_1, conf1	−2046.457811 (24.9)	-	-	-	-
	C_1, conf2	Evolves to C_1, conf1	-	-	-	-
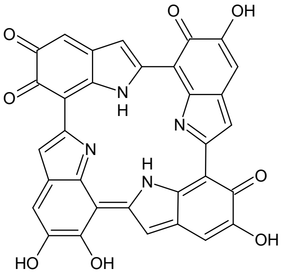 a56_b16_c6_d1	C_1, conf1	−2046.450907 (29.2)	-	-	-	-
	C_1, conf2	Evolves to C_1, conf1	-	-	-	-
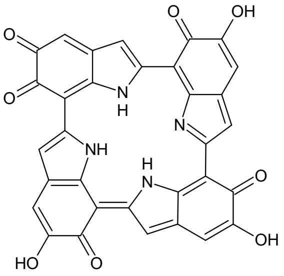 a56_b16_c6_d6	C_1, conf1	−2046.476681 (13.1)	-	-	-	-
	C_1, conf2	Evolves to C_1, conf1	-	-	-	-
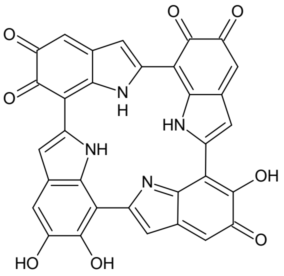 a56_b56_c15_d0	C_1, conf1	−2046.443973 (33.6)	-	-	-	-
	C_1, conf2	Evolves to C_1, conf1	-	-	-	-
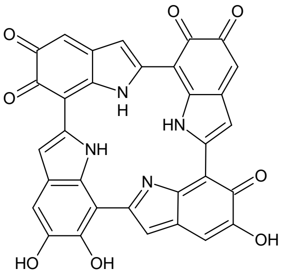 a56_b56_c16_d0	C_1, conf1	−2046.457076 (25.4)	-	-	-	-
	C_1, conf2	Evolves to C_1, conf1	-	-	-	-
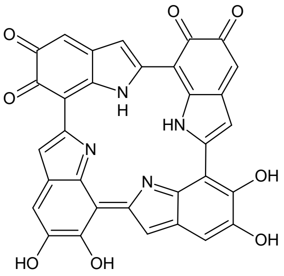 a56_b56_c1_d1	C_1, conf1	−2046.410179 (54.8)	-	-	-	-
	C_1, conf2	Evolves to C_1, conf1	-	-	-	-

Table S13. *Cont.*

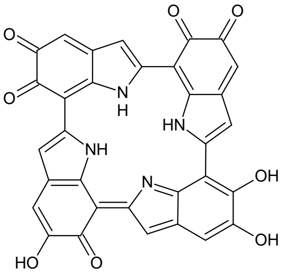 a56_b56_c1_d6	C_1, conf1	−2046.451473 (28.9)	-	-	-	-
	C_1, conf2	Evolves to C_1, conf1	-	-	-	-
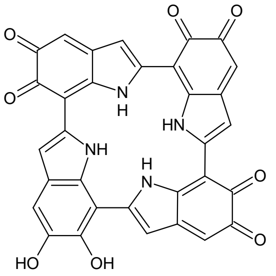 a56_b56_c56_d0	C_1, conf1	−2046.453263 (27.7)	-	-	-	-
	C_1, conf2	Evolves to C_1, conf1	-	-	-	-
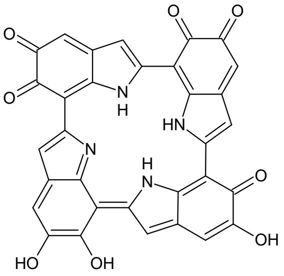 a56_b56_c6_d1	C_1, conf1	−2046.443910 (33.6)	-	-	-	-
	C_1, conf2	Evolves to C_1, conf1	-	-	-	-
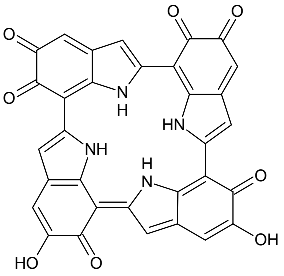 a56_b56_c6_d6	C_1, conf1	−2046.467092 (19.1)	-	-	-	-
	C_1, conf2	Evolves to C_1, conf1	-	-	-	-

In parentheses relative energies (kcal mol^{-1}) refer to the most stable form (in bold) identified at the specified level. [a] For chiral structures, only one enantiomer is listed. [b] Electronic energy including electrostatic contributions at the polarizable continuum model (PCM) level. [c] Enthalpy computed at 298.15 K within the rigid-rotor/harmonic-oscillator (RRHO) approximation. [d] Gibbs free energy computed at 298.15 K within the RRHO approximation. [e] Electronic energy including nonelectrostatic terms according to the SMD solvation model. [f] $G_{SMD,RRHO} = G_{PCM,RRHO} + G_{SMD} − G_{PCM}$.

Table S14. KP-8e, neutral form in vacuo.

Tautomer	Conformer [a]	E (Ha) [b]	H_{RRHO} (Ha) [c]	G_{RRHO} (Ha) [d]
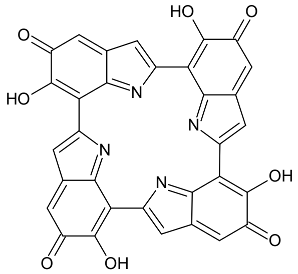 a15_b15_c15_d15	C_1, conf1	−2045.095671 (60.8)	-	-
	C_1, conf2	Evolves to C_1, conf1	-	-
	C_2, conf1	−2045.095507 (60.9)	-	-
	S_4, conf1	−2045.095507 (60.9)	-	-
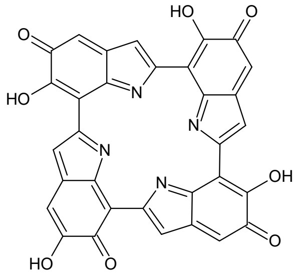 a15_b15_c15_d16	C_1, conf1	−2045.103800 (55.7)	-	-
	C_1, conf2	Evolves to C_1, conf1	-	-
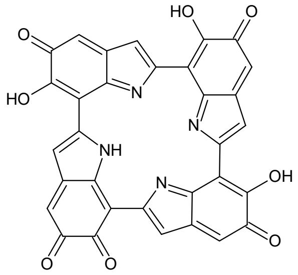 a15_b15_c15_d56	C_1, conf1	−2045.133771 (36.9)	-	-
	C_1, conf2	Evolves to C_1, conf1	-	-
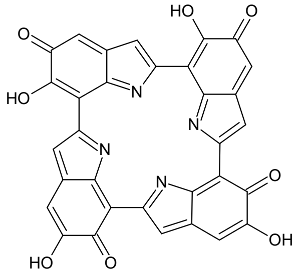 a15_b15_c16_d16	C_1, conf1	−2045.108918 (52.5)	-	-
	C_1, conf2	Evolves to C_1, conf1	-	-
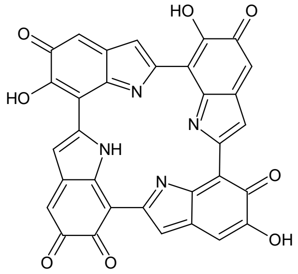 a15_b15_c16_d56	C_1, conf1	−2045.146806 (28.8)	-	-
	C_1, conf2	Evolves to C_1, conf1	-	-

Table S14. *Cont.*

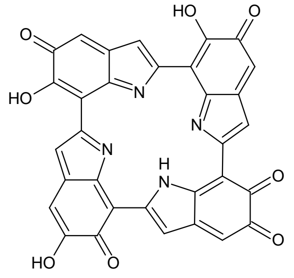 a15_b15_c56_d16	C_1, conf1	−2045.144224 (30.4)	-	-
	C_1, conf2	Evolves to C_1, conf1	-	-
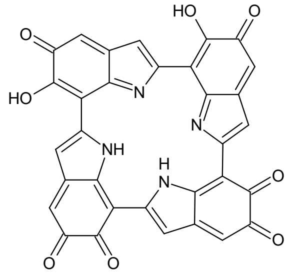 a15_b15_c56_d56	C_1, conf1	−2045.147256 (28.5)	-	-
	C_1, conf2	Evolves to C_1, conf1	-	-
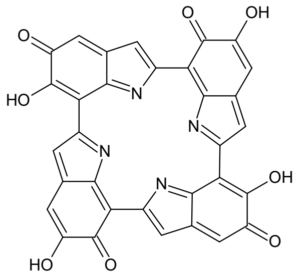 a15_b16_c15_d16	C_1, conf1	−2045.110767 (51.4)	-	-
	C_1, conf2	Evolves to C_1, conf1	-	-
	C_2, conf1	−2045.110691 (51.4)	-	-
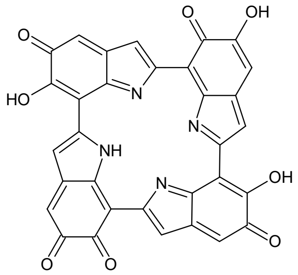 a15_b16_c15_d56	C_1, conf1	−2045.144951 (29.9)	-	-
	C_1, conf2	Evolves to C_1, conf1	-	-
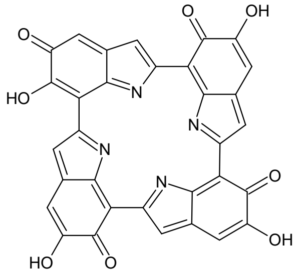 a15_b16_c16_d16	C_1, conf1	−2045.117040 (47.4)	-	-
	C_1, conf2	Evolves to C_1, conf1	-	-

Table S14. *Cont.*

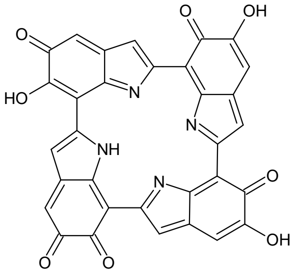 a15_b16_c16_d56	C_1, conf1	−2045.156421 (22.7)	-	-
	C_1, conf2	Evolves to C_1, conf1	-	-
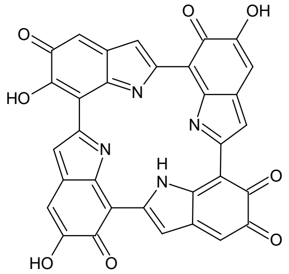 a15_b16_c56_d16	C_1, conf1	−2045.157314 (22.2)	-	-
	C_1, conf2	Evolves to C_1, conf1	-	-
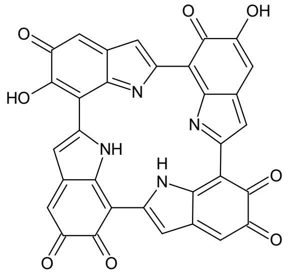 a15_b16_c56_d56	C_1, conf1	−2045.161829 (19.3)	-	-
	C_1, conf2	Evolves to C_1, conf1	-	-
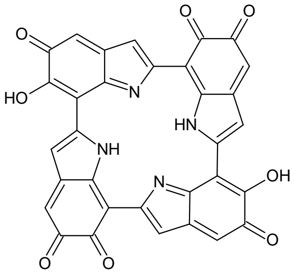 a15_b56_c15_d56	C_1, conf1	−2045.160273 (20.3)	-	-
	C_1, conf2	Evolves to C_1, conf1	-	-
	C_2, conf1	−2045.160264 (20.3)	-	-
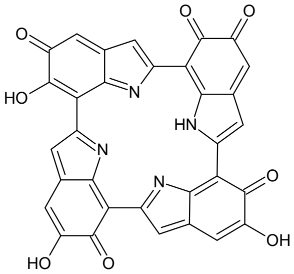 a15_b56_c16_d16	C_1, conf1	−2045.153063 (24.8)	-	-
	C_1, conf2	Evolves to C_1, conf1	-	-
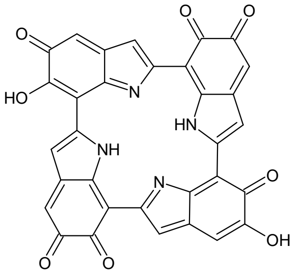 a15_b56_c16_d56	C_1, conf1	−2045.175692 (10.6)	-	-
	C_1, conf2	Evolves to C_1, conf1	-	-

Table S14. *Cont.*

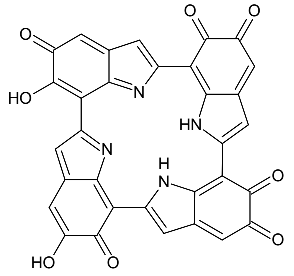<br>a15_b56_c56_d16	C_1, conf1	−2045.160978 (19.9)	-	-
	C_1, conf2	Evolves to C_1, conf1	-	-
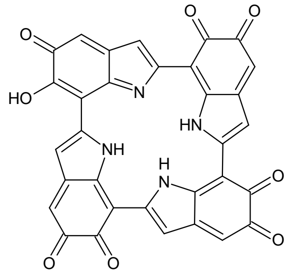<br>a15_b56_c56_d56	C_1, conf1	−2045.152820 (25.0)	-	-
	C_1, conf2	Evolves to C_1, conf1	-	-
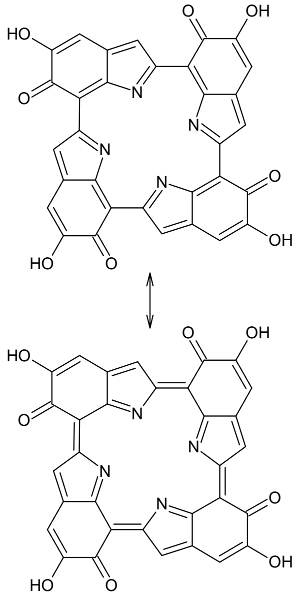<br>a16_b16_c16_d16	C_1, conf1	−2045.135255 (36.0)	-	-
	C_1, conf2	−2045.134761 (36.3)	-	-
	C_2, conf1	−2045.135210 (36.0)	-	-
	S_4, conf1	−2045.135218 (36.0)	-	-
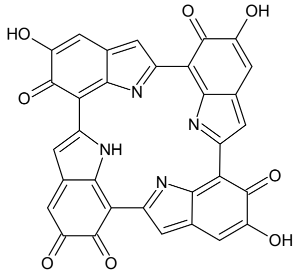<br>a16_b16_c16_d56	C_1, conf1	−2045.168123 (15.4)	-	-
	C_1, conf2	Evolves to C_1, conf1	-	-

Table S14. *Cont.*

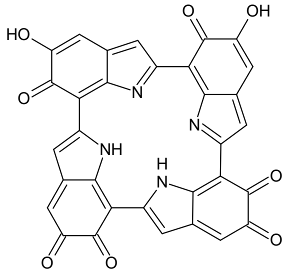 a16_b16_c56_d56	C_1, conf1	−2045.175948 (10.5)	-	-
	C_1, conf2	Evolves to C_1, conf1	-	-
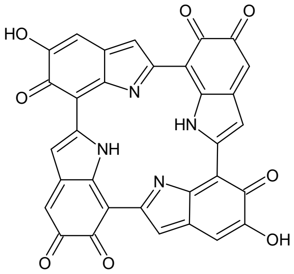 a16_b56_c16_d56	C_1, conf1	−2045.192843 (−0.1)	-	-
	C_1, conf2	−2045.192633 (0.0)	-	-
	C_2, conf1	**−2045.192633 (0.0)**	**−2044.778733 (0.0)**	**−2044.873783 (0.0)**
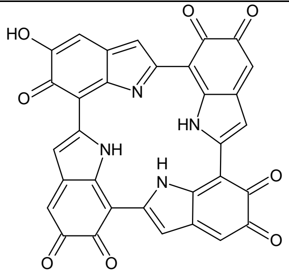 a16_b56_c56_d56	C_1, conf1	−2045.167811 (15.6)	-	-
	C_1, conf2	Evolves to C_1, conf1	-	-
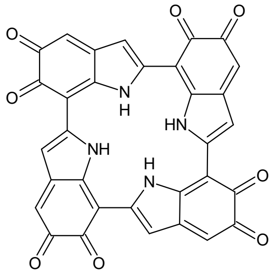 a56_b56_c56_d56	C_1, conf1	−2045.145802 (29.4)	-	-
	C_1, conf2	Evolves to C_1, conf1	-	-
	C_2, conf1	−2045.145651 (29.5)	-	-
	S_4, conf1	−2045.145651 (29.5)	-	-

In parentheses relative energies (kcal mol^{-1}) refer to the most stable form (in bold) identified at the specified level. [a] For chiral structures, only one enantiomer is listed. [b] Electronic energy. [c] Enthalpy computed at 298.15 K within the rigid-rotor/harmonic-oscillator (RRHO) approximation. [d] Gibbs free energy computed at 298.15 K within the RRHO approximation.

Table S15. KP-8e, neutral form in water.

Tautomer	Conformer [a]	G_{PCM} (Ha) [b]	$H_{PCM,RRHO}$ (Ha) [c]	$G_{PCM,RRHO}$ (Ha) [d]	G_{SMD} (Ha) [e]	$G_{SMD,RRHO}$ (Ha) [f]
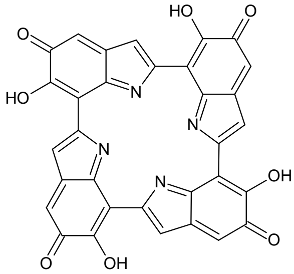 a15_b15_c15_d15	C_1, conf1	−2045.128582 (57.6)	-	-	-	-
	C_1, conf2	Evolves to C_1, conf1	-	-	-	-
	C_2, conf1	−2045.128516 (57.7)	-	-	-	-
	S_4, conf1	−2045.128519 (57.7)	-	-	-	-
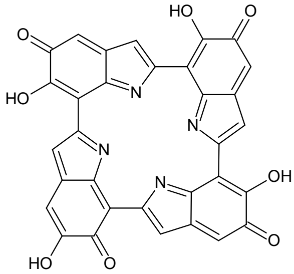 a15_b15_c15_d16	C_1, conf1	−2045.136787 (52.5)	-	-	-	-
	C_1, conf2	Evolves to C_1, conf1	-	-	-	-
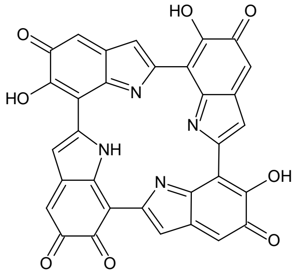 a15_b15_c15_d56	C_1, conf1	−2045.161697 (36.9)	-	-	-	-
	C_1, conf2	Evolves to C_1, conf1	-	-	-	-
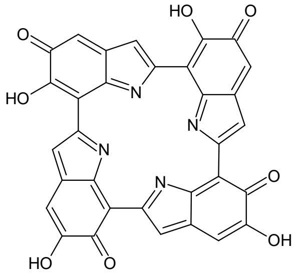 a15_b15_c16_d16	C_1, conf1	−2045.142212 (49.1)	-	-	-	-
	C_1, conf2	Evolves to C_1, conf1	-	-	-	-
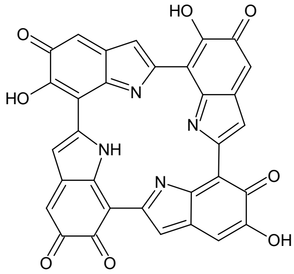 a15_b15_c16_d56	C_1, conf1	−2045.174488 (28.8)	-	-	-	-
	C_1, conf2	Evolves to C_1, conf1	-	-	-	-

Table S15. *Cont.*

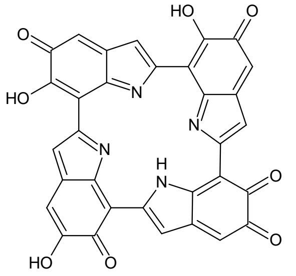 a15_b15_c56_d16	C_1, conf1	−2045.171117 (31.0)	-	-	-	-
	C_1, conf2	Evolves to C_1, conf1	-	-	-	-
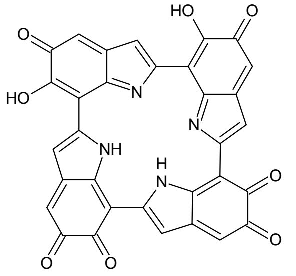 a15_b15_c56_d56	C_1, conf1	−2045.180499 (25.1)	-	-	-	-
	C_1, conf2	Evolves to C_1, conf1	-	-	-	-
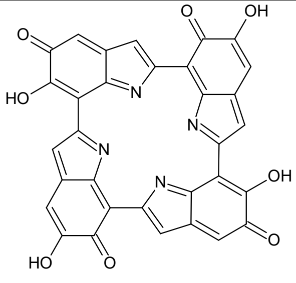 a15_b16_c15_d16	C_1, conf1	−2045.143561 (48.2)	-	-	-	-
	C_1, conf2	Evolves to C_1, conf1	-	-	-	-
	C_2, conf1	−2045.143472 (48.3)	-	-	-	-
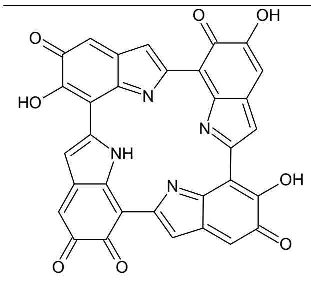 a15_b16_c15_d56	C_1, conf1	−2045.172151 (30.3)	-	-	-	-
	C_1, conf2	Evolves to C_1, conf1	-	-	-	-
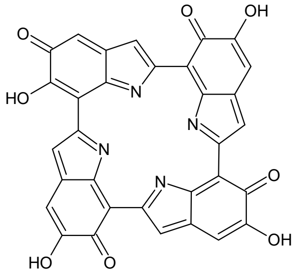 a15_b16_c16_d16	C_1, conf1	−2045.148908 (44.9)	-	-	-	-
	C_1, conf2	Evolves to C_1, conf1	-	-	-	-

Table S15. *Cont.*

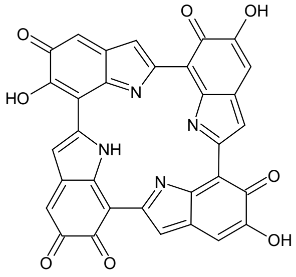 a15_b16_c16_d56	C_1, conf1	−2045.183144 (23.4)	-	-	-	-
	C_1, conf2	Evolves to C_1, conf1	-	-	-	-
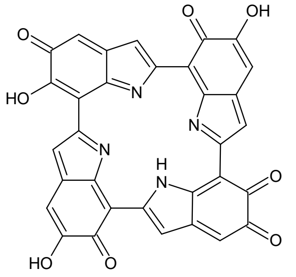 a15_b16_c56_d16	C_1, conf1	−2045.183833 (23.0)	-	-	-	-
	C_1, conf2	Evolves to C_1, conf1	-	-	-	-
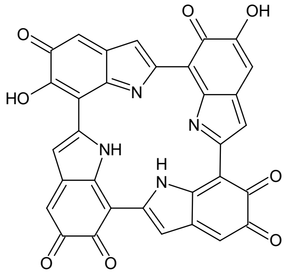 a15_b16_c56_d56	C_1, conf1	−2045.193141 (17.1)	-	-	-	-
	C_1, conf2	Evolves to C_1, conf1	-	-	-	-
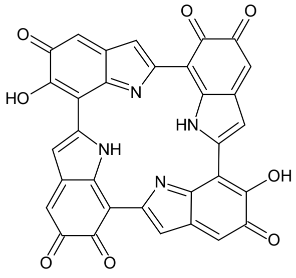 a15_b56_c15_d56	C_1, conf1	−2045.191121 (18.4)	-	-	-	-
	C_1, conf2	Evolves to C_1, conf1	-	-	-	-
	C_2, conf1	−2045.191026 (18.5)	-	-	-	-
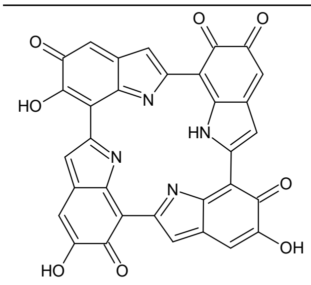 a15_b56_c16_d16	C_1, conf1	−2045.179225 (25.9)	-	-	-	-
	C_1, conf2	Evolves to C_1, conf1	-	-	-	-

Table S15. *Cont.*

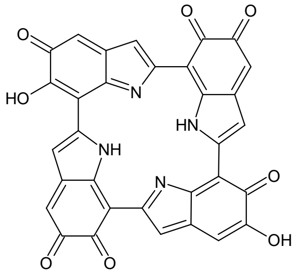 a15_b56_c16_d56	C_1, conf1	−2045.205085 (9.6)	-	-	-	-
	C_1, conf2	Evolves to C_1, conf1	-	-	-	-
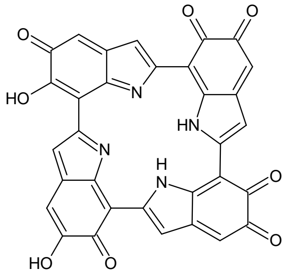 a15_b56_c56_d16	C_1, conf1	−2045.192540 (17.5)	-	-	-	-
	C_1, conf2	Evolves to C_1, conf1	-	-	-	-
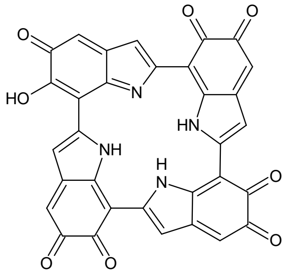 a15_b56_c56_d56	C_1, conf1	−2045.195946 (15.4)	-	-	-	-
	C_1, conf2	Evolves to C_1, conf1	-	-	-	-
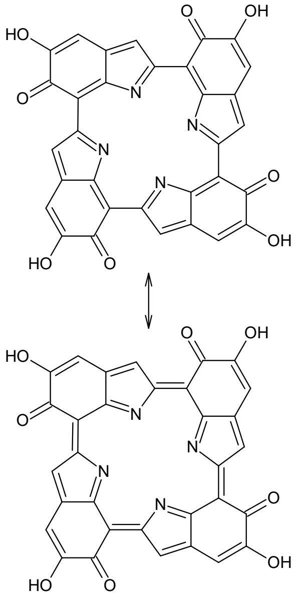 a16_b16_c16_d16	C_1, conf1	−2045.164720 (35.0)	-	-	-	-
	C_1, conf2	−2045.163869 (35.5)	-	-	-	-
	C_2, conf1	−2045.164067 (35.4)	-	-	-	-
	S_4, conf1	−2045.164067 (35.4)	-	-	-	-

Table S15. *Cont.*

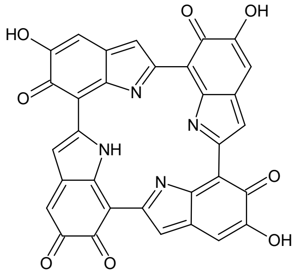 a16_b16_c16_d56	C_1, conf1	−2045.192953 (17.3)	-	-	-	-
	C_1, conf2	Evolves to C_1, conf1	-	-	-	-
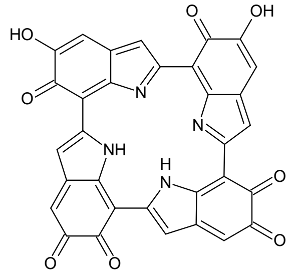 a16_b16_c56_d56	C_1, conf1	−2045.205247 (9.5)	-	-	-	-
	C_1, conf2	Evolves to C_1, conf1	-	-	-	-
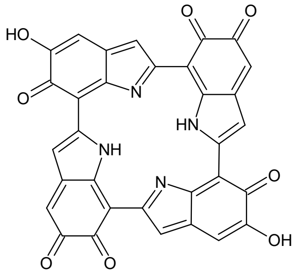 a16_b56_c16_d56	C_1, conf1	−2045.220522 (0.0)	-	-	-	-
	C_1, conf2	Evolves to C_1, conf1	-	-	-	-
	C_2, conf1	**−2045.220445 (0.0)**	**−2044.807661 (0.0)**	**−2044.902116 (0.0)**	**−2045.234643 (0.0)**	**−2044.916314 (0.0)**
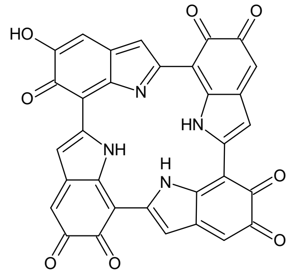 a16_b56_c56_d56	C_1, conf1	−2045.208959 (7.2)	-	-	-	-
	C_1, conf2	Evolves to C_1, conf1	-	-	-	-
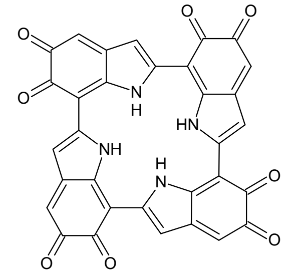 a56_b56_c56_d56	C_1, conf1	−2045.203005 (10.9)	-	-	-	-
	C_1, conf2	−2045.202789 (11.1)	-	-	-	-
	C_2, conf1	−2045.202794 (11.1)	-	-	-	-
	S_4, conf1	−2045.202886 (11.0)	-	-	-	-

In parentheses relative energies (kcal mol^{-1}) refer to the most stable form (in bold) identified at the specified level. [a] For chiral structures, only one enantiomer is listed. [b] Electronic energy including electrostatic contributions at the polarizable continuum model (PCM) level. [c] Enthalpy computed at 298.15 K within the rigid-rotor/harmonic-oscillator (RRHO) approximation. [d] Gibbs free energy computed at 298.15 K within the RRHO approximation. [e] Electronic energy including nonelectrostatic terms according to the SMD solvation model. [f] $G_{SMD,RRHO} = G_{PCM,RRHO} + G_{SMD} - G_{PCM}$.

Table S16. Excitations underlying the UV−Vis spectrum of a16_b6_c16_d6, computed at the C_{2h} geometry in vacuo.

Symmetry	Main CI contributions (coefficients)	λ (nm) (*f*)
^1B_u	HOMO−1 ($12a_u$) → LUMO ($13b_g$) (−0.12); HOMO ($13a_u$) → LUMO ($13b_g$) (0.60); HOMO ($13a_u$) → LUMO+1 ($14b_g$) (−0.32)	1080.6 (0.03)
^1B_u	HOMO−4 ($11a_u$) → LUMO+1 ($14b_g$) (0.14); HOMO−1 ($12a_u$) → LUMO ($13b_g$) (0.16); HOMO−1 ($12a_u$) → LUMO+1 ($14b_g$) (−0.15); HOMO ($13a_u$) → LUMO ($13b_g$) (0.32); HOMO ($13a_u$) → LUMO+1 ($14b_g$) (0.57)	988.5 (0.06)
^1A_g	HOMO−3 ($11b_g$) → LUMO ($13b_g$) (0.12); HOMO−2 ($12b_g$) → LUMO ($13b_g$) (−0.26); HOMO−2 ($12b_g$) → LUMO+1 ($14b_g$) (0.41); HOMO−1 ($12a_u$) → LUMO+2 ($14a_u$) (−0.11); HOMO ($13a_u$) → LUMO+2 ($14a_u$) (0.49)	779.9 (0.00)
^1B_u	HOMO−2 ($12b_g$) → LUMO+2 ($14a_u$) (−0.11); HOMO−1 ($12a_u$) → LUMO ($13b_g$) (0.34); HOMO−1 ($12a_u$) → LUMO+1 ($14b_g$) (0.59)	729.8 (0.02)
^1A_g	HOMO−2 ($12b_g$) → LUMO ($13b_g$) (0.64); HOMO−2 ($12b_g$) → LUMO+1 ($14b_g$) (0.22); HOMO ($13a_u$) → LUMO+2 ($14a_u$) (0.12)	714.7 (0.00)
^1A_g	HOMO−3 ($11b_g$) → LUMO ($13b_g$) (−0.35); HOMO−2 ($12b_g$) → LUMO+1 ($14b_g$) (0.46); HOMO−1 ($12a_u$) → LUMO+2 ($14a_u$) (−0.14); HOMO ($13a_u$) → LUMO+2 ($14a_u$) (−0.35)	679.8 (0.00)
^1B_u	HOMO−4 ($11a_u$) → LUMO ($13b_g$) (0.30); HOMO−1 ($12a_u$) → LUMO ($13b_g$) (0.51); HOMO−1 ($12a_u$) → LUMO+1 ($14b_g$) (−0.31); HOMO ($13a_u$) → LUMO+1 ($14b_g$) (−0.20)	678.2 (0.09)
^1A_g	HOMO−3 ($11b_g$) → LUMO ($13b_g$) (0.57); HOMO−2 ($12b_g$) → LUMO+1 ($14b_g$) (0.20); HOMO ($13a_u$) → LUMO+2 ($14a_u$) (−0.34)	638.0 (0.00)
^1A_g	HOMO−3 ($11b_g$) → LUMO ($13b_g$) (−0.12); HOMO−3 ($11b_g$) → LUMO+1 ($14b_g$) (0.67); HOMO−1 ($12a_u$) → LUMO+2 ($14a_u$) (0.14)	579.1 (0.00)
^1B_u	HOMO−5 ($10a_u$) → LUMO ($13b_g$) (0.20); HOMO−5 ($10a_u$) → LUMO+1 ($14b_g$) (0.17); HOMO−4 ($11a_u$) → LUMO ($13b_g$) (0.54); HOMO−3 ($11b_g$) → LUMO+2 ($14a_u$) (0.12); HOMO−2 ($12b_g$) → LUMO+2 ($14a_u$) (0.24); HOMO−1 ($12a_u$) → LUMO ($13b_g$) (−0.21)	568.8 (0.05)
^1B_u	HOMO−5 ($10a_u$) → LUMO ($13b_g$) (−0.36); HOMO−5 ($10a_u$) → LUMO+1 ($14b_g$) (0.12); HOMO−4 ($11a_u$) → LUMO+1 ($14b_g$) (0.49); HOMO−2 ($12b_g$) → LUMO+2 ($14a_u$) (0.29); HOMO−1 ($12a_u$) → LUMO+1 ($14b_g$) (0.10)	519.9 (0.04)
^1A_g	HOMO−3 ($11b_g$) → LUMO+1 ($14b_g$) (−0.16); HOMO−2 ($12b_g$) → LUMO+1 ($14b_g$) (0.15); HOMO−1 ($12a_u$) → LUMO+2 ($14a_u$) (0.66)	506.4 (0.00)
^1B_u	HOMO−5 ($10a_u$) → LUMO+1 ($14b_g$) (−0.36); HOMO−4 ($11a_u$) → LUMO ($13b_g$) (−0.15); HOMO−4 ($11a_u$) → LUMO+1 ($14b_g$) (−0.19); HOMO−2 ($12b_g$) → LUMO+2 ($14a_u$) (0.54)	487.2 (0.10)
^1B_u	HOMO−5 ($10a_u$) → LUMO ($13b_g$) (−0.42); HOMO−4 ($11a_u$) → LUMO+1 ($14b_g$) (−0.20); HOMO−3 ($11b_g$) → LUMO+2 ($14a_u$) (0.51); HOMO−1 ($12a_u$) → LUMO ($13b_g$) (−0.10)	458.4 (0.06)
^1B_u	HOMO−5 ($10a_u$) → LUMO ($13b_g$) (0.30); HOMO−5 ($10a_u$) → LUMO+1 ($14b_g$) (0.35); HOMO−4 ($11a_u$) → LUMO ($13b_g$) (−0.27); HOMO−4 ($11a_u$) → LUMO+1 ($14b_g$) (0.12); HOMO−3 ($11b_g$) → LUMO+2 ($14a_u$) (0.36); HOMO−2 ($12b_g$) → LUMO+2 ($14a_u$) (0.11); HOMO−1 ($12a_u$) → LUMO ($13b_g$) (0.15); HOMO ($13a_u$) → LUMO+1 ($14b_g$) (−0.13); HOMO ($13a_u$) → LUMO+4 ($15b_g$) (0.13)	430.7 (0.56)
^1A_g	HOMO−5 ($10a_u$) → LUMO+2 ($14a_u$) (0.12); HOMO−4 ($11a_u$) → LUMO+2 ($14a_u$) (0.68); HOMO−3 ($11b_g$) → LUMO ($13b_g$) (−0.11)	418.5 (0.00)
^1B_u	HOMO−5 ($10a_u$) → LUMO ($13b_g$) (−0.19); HOMO−5 ($10a_u$) → LUMO+1 ($14b_g$) (0.43); HOMO−4 ($11a_u$) → LUMO+1 ($14b_g$) (−0.35); HOMO−3 ($11b_g$) → LUMO+2 ($14a_u$) (−0.26); HOMO−2 ($12b_g$) → LUMO+2 ($14a_u$) (0.15); HOMO ($13a_u$) → LUMO ($13b_g$) (0.16); HOMO ($13a_u$) → LUMO+4 ($15b_g$) (0.11); HOMO ($13a_u$) → LUMO+5 ($16b_g$) (−0.12)	418.0 (0.79)
^1B_g	HOMO−10 ($61a_g$) → LUMO ($13b_g$) (0.24); HOMO−10 ($61a_g$) → LUMO+1 ($14b_g$) (0.20); HOMO−9 ($61b_u$) → LUMO+2 ($14a_u$) (−0.25); HOMO−8 ($62b_u$) → LUMO+2 ($14a_u$) (−0.10); HOMO−6 ($62a_g$) → LUMO ($13b_g$) (0.54); HOMO−6 ($62a_g$) → LUMO+1 ($14b_g$) (0.15)	407.1 (0.00)
^1A_u	HOMO−10 ($61a_g$) → LUMO+2 ($14a_u$) (−0.12); HOMO−9 ($61b_u$) → LUMO ($13b_g$) (0.49); HOMO−9 ($61b_u$) → LUMO+1 ($14b_g$) (0.14); HOMO−8 ($62b_u$) → LUMO ($13b_g$) (0.23); HOMO−8 ($62b_u$) → LUMO+1 ($14b_g$) (0.27); HOMO−6 ($62a_g$) → LUMO+2 ($14a_u$) (−0.28)	404.4 (0.00)

Only transitions with λ > 400 nm are listed.

Table S17. Excitations underlying the UV−Vis spectrum of the a16_b6_c16_d6 tautomer of KP-6e, computed in vacuo at the C_2 geometry.

Symmetry	Main CI contribtions (coefficients)	λ (nm) (*f*)
^{1}B	HOMO−1 (74a) → LUMO (75b) (−0.12); HOMO (75a) → LUMO (75b) (0.60); HOMO (75a) → LUMO+1 (76b) (−0.32)	1080.3 (0.03)
^{1}B	HOMO−4 (73a) → LUMO+1 (76b) (0.14); HOMO−1 (74a) → LUMO (75b) (0.16); HOMO−1 (74a) → LUMO+1 (76b) (−0.15); HOMO (75a) → LUMO (75b) (0.32); HOMO (75a) → LUMO+1 (76b) (0.57)	988.1 (0.06)
^{1}A	HOMO−3 (73b) → LUMO (75b) (0.12); HOMO−2 (74b) → LUMO (75b) (−0.26); HOMO−2 (74b) → LUMO+1 (76b) (0.41); HOMO−1 (74a) → LUMO+2 (76a) (−0.11); HOMO (75a) → LUMO+2 (76a) (0.49)	779.6 (0.00)
^{1}B	HOMO−2 (74b) → LUMO+2 (76a) (−0.11); HOMO−1 (74a) → LUMO (75b) (0.35); HOMO−1 (74a) → LUMO+1 (76b) (0.59)	729.4 (0.02)
^{1}A	HOMO−2 (74b) → LUMO (75b) (0.64); HOMO−2 (74b) → LUMO+1 (76b) (0.22); HOMO (75a) → LUMO+2 (76a) (0.12)	715.0 (0.00)
^{1}A	HOMO−3 (73b) → LUMO (75b) (−0.36); HOMO−2 (74b) → LUMO+1 (76b) (0.46); HOMO−1 (74a) → LUMO+2 (76a) (−0.14); HOMO (75a) → LUMO+2 (76a) (−0.35)	679.7 (0.00)
^{1}B	HOMO−4 (73a) → LUMO (75b) (0.30); HOMO−1 (74a) → LUMO (75b) (0.50); HOMO−1 (74a) → LUMO+1 (76b) (−0.32); HOMO (75a) → LUMO+1 (76b) (−0.20)	678.2 (0.09)
^{1}A	HOMO−3 (73b) → LUMO (75b) (0.57); HOMO−2 (74b) → LUMO+1 (76b) (0.20); HOMO (75a) → LUMO+2 (76a) (−0.34)	638.0 (0.00)
^{1}A	HOMO−3 (73b) → LUMO (75b) (−0.12); HOMO−3 (73b) → LUMO+1 (76b) (0.67); HOMO−1 (74a) → LUMO+2 (76a) (0.14)	579.0 (0.00)
^{1}B	HOMO−5 (72a) → LUMO (75b) (0.20); HOMO−5 (72a) → LUMO+1 (76b) (0.17); HOMO−4 (73a) → LUMO (75b) (0.54); HOMO−3 (73b) → LUMO+2 (76a) (0.12); HOMO−2 (74b) → LUMO+2 (76a) (0.24); HOMO−1 (74a) → LUMO (75b) (−0.21)	568.9 (0.05)
^{1}B	HOMO−5 (72a) → LUMO (75b) (−0.36); HOMO−5 (72a) → LUMO+1 (76b) (0.13); HOMO−4 (73a) → LUMO+1 (76b) (0.49); HOMO−2 (74b) → LUMO+2 (76a) (0.29); HOMO−1 (74a) → LUMO+1 (76b) (0.10)	520.0 (0.04)
^{1}A	HOMO−3 (73b) → LUMO+1 (76b) (−0.16); HOMO−2 (74b) → LUMO+1 (76b) (0.15); HOMO−1 (74a) → LUMO+2 (76a) (0.66)	506.2 (0.00)
^{1}B	HOMO−5 (72a) → LUMO+1 (76b) (−0.36); HOMO−4 (73a) → LUMO (75b) (−0.15); HOMO−4 (73a) → LUMO+1 (76b) (−0.18); HOMO−2 (74b) → LUMO+2 (76a) (0.54)	487.3 (0.10)
^{1}B	HOMO−5 (72a) → LUMO (75b) (−0.42); HOMO−4 (73a) → LUMO+1 (76b) (−0.20); HOMO−3 (73b) → LUMO+2 (76a) (0.51); HOMO−1 (74a) → LUMO (75b) (−0.10)	458.4 (0.06)
^{1}B	HOMO−5 (72a) → LUMO (75b) (0.30); HOMO−5 (72a) → LUMO+1 (76b) (0.35); HOMO−4 (73a) → LUMO (75b) (−0.27); HOMO−4 (73a) → LUMO+1 (76b) (0.12); HOMO−3 (73b) → LUMO+2 (76a) (0.36); HOMO−2 (74b) → LUMO+2 (76a) (0.11); HOMO−1 (74a) → LUMO (75b) (0.15); HOMO (75a) → LUMO+1 (76b) (−0.13); HOMO (75a) → LUMO+4 (77b) (0.13)	430.8 (0.56)
^{1}A	HOMO−5 (72a) → LUMO+2 (76a) (0.12); HOMO−4 (73a) → LUMO+2 (76a) (0.68); HOMO−3 (73b) → LUMO (75b) (−0.11)	418.5 (0.00)
^{1}B	HOMO−5 (72a) → LUMO (75b) (−0.19); HOMO−5 (72a) → LUMO+1 (76b) (0.42); HOMO−4 (73a) → LUMO+1 (76b) (−0.35); HOMO−3 (73b) → LUMO+2 (76a) (−0.26); HOMO−2 (74b) → LUMO+2 (76a) (0.15); HOMO (75a) → LUMO (75b) (0.16); HOMO (75a) → LUMO+4 (77b) (0.11); HOMO (75a) → LUMO+5 (78b) (−0.12)	418.2 (0.79)
^{1}B	HOMO−10 (70a) → LUMO (75b) (−0.24); HOMO−10 (70a) → LUMO+1 (76b) (−0.20); HOMO−9 (70b) → LUMO+2 (76a) (−0.25); HOMO−6 (71a) → LUMO (75b) (0.54); HOMO−6 (71a) → LUMO+1 (76b) (0.15)	406.9 (0.00)
^{1}A	HOMO−10 (70a) → LUMO+2 (76a) (0.12); HOMO−9 (70b) → LUMO (75b) (0.50); HOMO−9 (70b) → LUMO+1 (76b) (0.15); HOMO−8 (71b) → LUMO (75b) (−0.19); HOMO−8 (71b) → LUMO+1 (76b) (−0.24); HOMO−7 (72b) → LUMO (75b) (−0.12); HOMO−7 (72b) → LUMO+1 (76b) (−0.12); HOMO−6 (71a) → LUMO+2 (76a) (−0.27)	404.4 (0.00)

Only transitions with λ > 400 nm are listed.

Table S18. Comparison of the electronic energies (Ha) of the main tautomers/conformers of the neutral form of KP-4e, computed in vacuo at different theory levels at the PBE0/6-31+G(d,p) geometry (see also Table S10).

Tautomer/Conformer	PBE0/ 6-31+G(d,p)	PBE0/ 6-311+G(d,p)	PBE0/ 6-311++G(2d,2p)	B3LYP/ 6-31+G(d,p)	B3LYP/ 6-311++G(2d,2p)
a16_b6_c6_d0, C_1, conf1	−2047.700139 (1.3)	−2048.109478 (1.2)	−2048.177153 (1.2)	−2049.940498 (1.4)	−2050.446880 (1.4)
a6_b16_c6_d0, C_1, conf1	**−2047.702153 (0.0)**	**−2048.111448 (0.0)**	**−2048.179136 (0.0)**	**−2049.942664 (0.0)**	**−2050.449034 (0.0)**
a6_b6_c16_d0, C_1, conf1	−2047.699471 (1.7)	−2048.108829 (1.6)	−2048.176600 (1.6)	−2049.940014 (1.7)	−2050.446511 (1.6)
a6_b6_c6_d6, C_2, conf1	−2047.696144 (3.8)	−2048.105714 (3.6)	−2048.174495 (2.9)	−2049.937247 (3.4)	−2050.444927 (2.6)

In parentheses relative energies (kcal mol^{-1}) referred to the most stable form (in bold) identified at the specified level.

Table S19. Electronic energies (Ha) of main neutral species of KP computed in vacuo at different theory levels, at the PBE0/6-31+G(d,p) geometry.

Species	PBE0/ 6-31+G(d,p)	PBE0/ 6-311+G(d,p)	PBE0/ 6-311++G(2d,2p)	B3LYP/ 6-31+G(d,p)	B3LYP/ 6-311++G(2d,2p)
KP-Red, a0_b0_c0_d0, S_4	−2050.155247	−2050.565918	−2050.633365	−2052.392122	−2052.900872
KP-1e, a6_b0_c0_d0, C_1, conf1	−2049.541050	−2049.951113	−2050.018443	−2051.778022	−2052.285681
KP-2e, a6_b6_c0_d0, C_1, conf1	−2048.929568	−2049.339650	−2049.407493	−2051.168584	−2051.676482
KP-4e, a6_b16_c6_d0, C_1, conf1	−2047.702153	−2048.111448	−2048.179136	−2049.942664	−2050.449034
KP-6e, a16_b6_c16_d6, C_2, conf1	−2046.482242	−2046.890520	−2046.958280	−2048.724088	−2049.228878
KP-8e, a16_b56_c16_d56, C_2, conf1	−2045.192633	−2045.601346	−2045.666471	−2047.437272	−2047.939954

Table S20. Electronic energy changes (kcal mol^{-1}) for disproportionation processes involving KP, computed with the data of Table S19.

Reaction	PBE0/ 6-31+G(d,p)	PBE0/ 6-311+G(d,p)	PBE0/ 6-311++G(2d,2p)	B3LYP/ 6-31+G(d,p)	B3LYP/ 6-311++G(2d,2p)
2 KP-1e ⇌ KP-Red + KP-2e	−1.7	−2.1	−2.5	−2.9	−3.8
2 KP-2e ⇌ KP-Red + KP-4e	1.1	1.2	1.6	1.5	1.9
2 KP-4e ⇌ KP−2e + KP-6e	−4.7	−4.6	−4.7	−4.6	−4.6
2 KP-6e ⇌ KP-4e + KP-8e	43.7	42.8	44.5	42.8	43.2

Table S21. Comparison of the electronic transitions (eV) of porphin free base (D_2 symmetry), computed in vacuo at different levels, using the PBE0/6-31+G(d,p) geometries.

**TD-PBE0/6-311++G(2d,2p)**		**TD-B3LYP/6-311++G(2d,2p)**	
Symmetry	λ (nm) (*f*)	Symmetry	λ (nm) (*f*)
B_{1U}	537.9 (0.000)	B_{1U}	545.1 (0.000)
B_{2U}	504.8 (0.000)	B_{2U}	512.8 (0.000)
B_{1U}	365.9 (0.590)	B_{1U}	373.8 (0.499)
B_{2U}	351.9 (0.911)	B_{2U}	358.8 (0.754)
B_{2U}	320.7 (0.283)	B_{2U}	332.3 (0.414)
B_{1U}	317.3 (0.662)	B_{1U}	326.0 (0.706)
B_{3U}	287.5 (0.001)	B_{3U}	297.9 (0.001)
B_{2U}	276.3 (0.102)	B_{2U}	284.8 (0.096)
B_{1U}	268.2 (0.106)	B_{1U}	278.3 (0.101)
B_{3U}	248.2 (0.006)	B_{3U}	260.9 (0.005)

Only transition of $^1B_{1u}$, $^1B_{2u}$ and $^1B_{3u}$ symmetry are listed.

Table S22. Comparison of the electronic transitions (eV) of magnesium–porphin (D_{4h} symmetry), computed in vacuo at different levels, using the PBE0/6-31+G(d,p) geometries.

**TD-PBE0/6-311++G(2d,2p)**		**TD-B3LYP/6-311++G(2d,2p)**	
Symmetry	λ (nm) (*f*)	Symmetry	λ (nm) (*f*)
E_U	518.3 (0.001)	E_U	525.6 (0.001)
E_U	518.3 (0.001)	E_U	525.6 (0.001)
E_U	352.6 (1.020)	E_U	357.9 (0.973)
E_U	352.6 (1.020)	E_U	357.9 (0.973)
E_U	318.4 (0.039)	E_U	329.7 (0.042)
E_U	318.4 (0.039)	E_U	329.7 (0.042)
E_U	284.3 (0.213)	E_U	294.5 (0.214)
E_U	284.3 (0.213)	E_U	294.5 (0.214)
A_{2U}	259.3 (0.001)	A_{2U}	271.7 (0.001)

Only transitions of A_{2u} and E_u symmetry are listed.

References

1. Nakamura, S.; Hiroto, S.; Shinokubo, H. Synthesis and oxidation of cyclic tetraindole. *Chem. Sci.* **2012**, *3*, 524–527.
